# Supplementary material for: A framework for integrating directed and undirected annotations to build explanatory models of cis-eQTL data
Source: PLoS Comput Biol. 2020 Jun 9;16(6):e1007770. doi: 10.1371/journal.pcbi.1007770 (PMC7332077; doi:10.1371/journal.pcbi.1007770)
Supplement: S1 Appendix — (PDF) [file pcbi.1007770.s001.pdf]

# 1 Supporting Methods

## Contents


**1 Supporting Methods** **1**

1.1 Outline of Variational Message Passing . . . . . . . . . . . . . . . . . . . . . . . . . . . 2

1.1.1 Conjugate-Exponential Models . . . . . . . . . . . . . . . . . . . . . . . . . . 2

1.1.2 Form of Messages . . . . . . . . . . . . . . . . . . . . . . . . . . . . . . . . . 4

1.2 Implementing the *BAGEA* Model . . . . . . . . . . . . . . . . . . . . . . . . . . . . . 5

1.2.1 Log-Probability Densities . . . . . . . . . . . . . . . . . . . . . . . . . . . . . 5

1.2.2 Expectation of Natural Parameter Vector $\boldsymbol{\phi}$: w.r.t $\boldsymbol{Q}$ . . . . . . . . . . . . 6

1.2.3 Expectation of Sufficient Statistic Vector $E[\boldsymbol{u_x}]$ as a Function of $\boldsymbol{\phi}^*_{\boldsymbol{x}}$ (Messages to Children) . . . . . . . . . . . . . . . . . . . . . . . . . . . . . . . . . 6

1.2.4 Messages from Children to Parents . . . . . . . . . . . . . . . . . . . . . . . 7

1.2.5 Approximating -$\boldsymbol{\phi}^*_{\boldsymbol{b}}(2)$ for Fast Inversion . . . . . . . . . . . . . . . . . . . . 8

1.2.6 Working with Summary Statistics. . . . . . . . . . . . . . . . . . . . . . . . . 9

1.2.7 Computing the Lower Bound of the Data Log-Evidence . . . . . . . . . . 10

1.3 Default Parameter Settings . . . . . . . . . . . . . . . . . . . . . . . . . . . . . . . . 12

1.4 ExPecto Comparison . . . . . . . . . . . . . . . . . . . . . . . . . . . . . . . . . . . . 12

1.5 Simulation . . . . . . . . . . . . . . . . . . . . . . . . . . . . . . . . . . . . . . . . . . 13

1.6 Estimating $E[b^2_{ij}]$ Conditional on Global *BAEGA* Parameters. . . . . . . . . . . . 14

1.7 Torus Comparison . . . . . . . . . . . . . . . . . . . . . . . . . . . . . . . . . . . . . 15

## 1.1 Outline of Variational Message Passing

To fit the *BAGEA* model, we use variational message passing**?**. We will very briefly outline the algorithm but encourage the reader to consult the literature for a fuller understanding. The model prescribes a distribution $P[\boldsymbol{H}, \boldsymbol{V}]$ over the hidden nodes $\boldsymbol{H}$ and visible nodes $\boldsymbol{V}$. The goal is to be able to marginalize over this model such that we can compute, say, $E[\boldsymbol{H_i}|\boldsymbol{V}]$ for a particular node $\boldsymbol{H_i}$ of interest. Variational message passing approximates $P[\boldsymbol{H}|\boldsymbol{V}]$ with a more tractable distribution $Q(\boldsymbol{H})$ over the hidden nodes with the property that each hidden node in the network is independent of all others (i.e. $Q(\boldsymbol{H}) = \prod_i Q_{H_i}(\boldsymbol{H_i})$). This distribution is chosen such that it minimizes the Kullback-Leibler (KL) divergence to $P[\boldsymbol{H}|\boldsymbol{V}]$. Variational message passing is designed to iteratively update $Q$ to minimize the KL divergence (convergence to the optimum is not guaranteed however, as the procedure can get trapped in local optima). It can be regarded as coordinate descent, where the distribution $Q_{H_i}$ gets updated separately while keeping the other distributions $Q_{H_j}$ $j \neq i$ constant. It can be shown that the optimal distribution $Q^*_{H_i}$ in terms of the KL divergence has the following property: $\log(Q^*_{H_i}(\boldsymbol{H_i})) =< \log(P[\boldsymbol{V}, \boldsymbol{H}]) >_{\sim Q_{H_i}(\boldsymbol{H_i})}$, where $< . >_{\sim Q_{H_i}(\boldsymbol{H_i})}$ refers to the expectation w.r.t to all $Q_{H_j}(\boldsymbol{H_j})$ with $i \neq j$.

### 1.1.1 Conjugate-Exponential Models

To be able to run variational message passing, the model has to be conjugate-exponential. To satisfy the exponentiality condition, all random variable nodes, conditional on their parent nodes, have to be members of the exponential family and should be writable in the following form:

$$\log(P[\boldsymbol{x}]) = \boldsymbol{\phi_x}^T \boldsymbol{u_x}(\boldsymbol{x}) + g(\boldsymbol{\phi_x}) + f(\boldsymbol{x}), \tag{1}$$

where $\boldsymbol{u_x}(\boldsymbol{x})$ is the sufficient statistic vector and $\boldsymbol{\phi_x}$ is the natural parameter vector. To make the conditional character explicit, we can write

$$\log(P[\boldsymbol{x}|\boldsymbol{pa_x}]) = \boldsymbol{\phi_x}(\boldsymbol{pa_x})^T \boldsymbol{u_x}(\boldsymbol{x}) + g(\boldsymbol{\phi_x}(\boldsymbol{pa_x})) + f(\boldsymbol{x}), \tag{2}$$

where $\boldsymbol{pa_x}$ denotes all parents of $\boldsymbol{x}$. To satisfy the conjugacy condtion, the conditional log probability of $\boldsymbol{x}$ needs to follow the same functional form as the log probability of its parent $\boldsymbol{y}$:

$$\log(P[\boldsymbol{x}|\boldsymbol{y}, \boldsymbol{cp_y(x)}]) = \boldsymbol{\phi_{xy}(x, cp_y(x))}^T \boldsymbol{u_y}(\boldsymbol{y}) + \lambda(\boldsymbol{x}, \boldsymbol{cp_y(x)}), \tag{3}$$

where $\boldsymbol{cp_y(x)}$ denotes all other parents of $\boldsymbol{x}$ except $\boldsymbol{y}$, i.e. the coparents of $\boldsymbol{y}$. Put another way, one needs to be able to express the dependence of $\log(P[\boldsymbol{x}|\boldsymbol{y}, \boldsymbol{cp_y(x)}])$ on $\boldsymbol{y}$ as a linear function of the sufficient statistic vector $\boldsymbol{u_y}(\boldsymbol{y})$. Analogously, this condition has to hold for all other parents of $\boldsymbol{x}$ too. As a consequence of this, we can write the entire distribution $\log(P[\boldsymbol{V}, \boldsymbol{H}])$ as depending on $\boldsymbol{y}$ via its sufficient statistic vector $\boldsymbol{u_y}(\boldsymbol{y})$ as follows:

$$\log(P[\boldsymbol{V}, \boldsymbol{H}]) = \Big(\boldsymbol{\phi_y(pa_y)} + \sum_{x_i \in \boldsymbol{ch_y}} \boldsymbol{\phi_{x_i y}(x_i, cp_y(x_i))}\Big)\boldsymbol{u_y}(\boldsymbol{y}) + f(\boldsymbol{y}) + \boldsymbol{g}, \tag{4}$$

where $\boldsymbol{g}$ is independent of $\boldsymbol{y}$, $\boldsymbol{ch_y}$ denotes the set of children of $\boldsymbol{y}$ and $\boldsymbol{cp_y(x_i)})$ denotes the set of parents of $\boldsymbol{x_i}$ except $\boldsymbol{y}$. As we can see, if we fix all variables except $\boldsymbol{y}$, the resulting distribution is in the same exponential family as $\boldsymbol{x}$ conditional on its parents, i.e. $\boldsymbol{x}|\boldsymbol{pa_x}$. Moreover, if we take expectations w.r.t. to $< . >_{\sim Q_{\boldsymbol{y}}(\boldsymbol{y})}$, we see that the resulting distribution still is in the same exponential family. To put it another way: for any $\boldsymbol{H_i}$, $Q_{H_i}(\boldsymbol{H_i})$ has the same functional form as $P[\boldsymbol{H_i}|\boldsymbol{pa_{H_i}}]$, where $\boldsymbol{pa_{H_i}}$ is the parents of $\boldsymbol{H_i}$. For instance, if $P[\boldsymbol{H_i}|\boldsymbol{pa_{H_i}}]$ has a normal density, then so does $Q_i(\boldsymbol{H_i})$.

To update $Q_{\boldsymbol{y}}(\boldsymbol{y})$, we need to be able to calculate $< \boldsymbol{\phi_y}(\boldsymbol{pa_y}) >_{\sim Q_{\boldsymbol{y}}}$ and $\boldsymbol{\phi_{x_i y}(x_i, cp_y(x_i))}_{\sim Q_{\boldsymbol{y}}}$ for all children $\boldsymbol{x_i}$ of $\boldsymbol{y}$. This will allow us to calculate the natural parameter vector for the updated $Q_{\boldsymbol{y}}$. Note that, because of conjugacy, and all $Q_{H_i}$ being independent from each other, $< \boldsymbol{\phi_{xy}(x, cp_y)} >$ can be expressed as a function of the expectation of the sufficient statistic vector $E_{Q_{\boldsymbol{x}}}[\boldsymbol{u_x}(x)]$ as well as the expectation of the sufficient statistic vector of all coparents of $\boldsymbol{y}$.

### 1.1.2 Form of Messages

The message passing algorithm works by sending messages to neighboring nodes and local updates at the nodes once the messages are gathered. Messages sent from child $\boldsymbol{x}$ to parent $\boldsymbol{y}$ has the form

$$< \boldsymbol{\phi_{xy}(x, cp_y(x))} >, \tag{5}$$

where $< \cdot >$ denotes the expectation with respect $Q(\boldsymbol{H})$ (in practice we do not know $Q(\boldsymbol{H})$ as this is the quantity we are trying to find. We plug in our current estimate instead). Again, because of conjugacy and all $Q_{H_i}$ being independent from each other, $< \boldsymbol{\phi_{xy}(x, cp_y)} >$ can be expressed as a function of the expectation of the sufficient statistic vector $E[\boldsymbol{u_x}]$ as well as the expectation of the sufficient statistic vector of all coparents of $\boldsymbol{y}$. Messages passed from a parent $\boldsymbol{z}$ of $\boldsymbol{y}$ to $\boldsymbol{y}$ is just the expectation of the sufficient statistic vector $E[\boldsymbol{u_z}]$ itself. These messages are then used to compute

$$< \boldsymbol{\phi_y(pa_y)} > \tag{6}$$

Once the messages are received, we want to update $Q_{\boldsymbol{y}}(\boldsymbol{y})$ itself. As we noted before, $Q_{\boldsymbol{y}}(\boldsymbol{y})$ has the same functional form as $P[\boldsymbol{y}|\boldsymbol{pa_y}]$, i.e. it is in the same exponential family. We therefore only need to find its natural parameter vector $\boldsymbol{\phi^*_y}$. This can be computed from the sent messages:

$$\boldsymbol{\phi^*_y} =< \boldsymbol{\phi_y(pa_z)} > + \sum_{x_i \in \boldsymbol{ch_y}} < \boldsymbol{\phi_{x_i y}(x_i, cp_y(x_i))} >, \tag{7}$$

where $\boldsymbol{ch_y}$ denotes the set of children of $\boldsymbol{y}$ and $\boldsymbol{cp_y(x_i)})$ denotes the set of parents of $\boldsymbol{x_i}$ except $\boldsymbol{y}$. Once we know $\boldsymbol{\phi^*_y}$, we have implicitly determined an updated distribution $Q_{\boldsymbol{y}}(\boldsymbol{y})$ and can find the coresponding expectations of its sufficient statistic vector $E[\boldsymbol{u_y}]$. This serves again as the message to all children of $\boldsymbol{y}$ and is used in the computation of messages to its parents.

## 1.2 Implementing the *BAGEA* Model

### 1.2.1 Log-Probability Densities

In the following, we list all log probability densities of the *BAGEA* model described in the methods section:

$$\log(P[\boldsymbol{y}_j|\lambda_j, \boldsymbol{b}_j]) = -\frac{n}{2}\log(2\pi) + \frac{n}{2}\log(\lambda_j) - \frac{\lambda_j}{2}(\boldsymbol{y}_j^T\boldsymbol{y}_j - 2\boldsymbol{y}_j^T\boldsymbol{X}_j\boldsymbol{b}_j + \boldsymbol{b}_j^T\boldsymbol{X}_j^T\boldsymbol{X}_j\boldsymbol{b}_j),$$

$$\log(P[\lambda_j|\lambda_1, \lambda_2]) = \lambda_1\log(\lambda_2) - \log(\Gamma(\lambda_1)) + (\lambda_1 - 1)\log(\lambda_j) - \lambda_2\lambda_j,$$

$$\log(P[\lambda_2|\rho_1, \rho_2]) = \rho_1\log(\rho_2) - \log(\Gamma(\rho_1)) + (\rho_1 - 1)\log(\lambda_2) - \rho_2\lambda_2,$$

$$\log(P[\alpha_{ij}|\gamma_1, \gamma_{ij}, \kappa_j]) = \gamma_1\Big(\log(\kappa_j) + \log(\gamma_{ij})\Big) - \log(\Gamma(\gamma_1)) + (\gamma_1 - 1)\log(\alpha_{ij}) - \kappa_j\gamma_{ij}\alpha_{ij},$$

i.e.:

$$\log(P[\alpha_{ij}|a_1, .., a_t]) = \gamma_1\Big(\log(\kappa_j) + \sum_{k:\boldsymbol{C}^{\boldsymbol{j}}_{ik}=1}\log(a_k)\Big) - \log(\Gamma(\gamma_1)) + (\gamma_1 - 1)\log(\alpha_{ij}) - \alpha_{ij}\kappa_j\prod_{k:\boldsymbol{C}^{\boldsymbol{j}}_{ik}=1}a_k,$$

$$\log(P[a_k|\phi_1, \phi_2]) = \phi_1\log(\phi_2) - \log(\Gamma(\phi_1)) + (\phi_1 - 1)\log(a_k) - \phi_2 a_k,$$

$$\log(P[\kappa_j|\tau_1, \tau_2]) = \tau_1\log(\tau_2) - \log(\Gamma(\tau_1)) + (\tau_1 - 1)\log(\kappa_j) - \tau_2\kappa_j,$$

$$\log(P[\tau_2|\xi_1, \xi_2]) = \xi_1\log(\xi_2) - \log(\Gamma(\xi_1)) + (\xi_1 - 1)\log(\tau_2) - \xi_2\tau_2,$$

$$\begin{aligned}\log(P[b_{ij}|\boldsymbol{\nu}, \boldsymbol{\omega}, \alpha_{ij}]) = & - \frac{1}{2}\log(2\pi) + \frac{1}{2}\log(\alpha_{ij}) - \frac{\alpha_{ij}}{2}(b_{ij}^2 + ((\boldsymbol{v}^{\boldsymbol{j}}_{\boldsymbol{i}})^T\boldsymbol{\omega})^2((\boldsymbol{f}^{\boldsymbol{j}}_{\boldsymbol{i}})^T\boldsymbol{\nu})^2 \\ & - 2b_{ij}((\boldsymbol{v}^{\boldsymbol{j}}_{\boldsymbol{i}})^T\boldsymbol{\omega})((\boldsymbol{f}^{\boldsymbol{j}}_{\boldsymbol{i}})^T\boldsymbol{\nu})),\end{aligned}$$

where $\boldsymbol{v}^{\boldsymbol{j}}_{\boldsymbol{i}}$ and $\boldsymbol{f}^{\boldsymbol{j}}_{\boldsymbol{i}}$ are the $i$th row vector of $\boldsymbol{V}^{\boldsymbol{j}}$ and $\boldsymbol{F}^{\boldsymbol{j}}$ respectively. Further,

$$\log(P[\boldsymbol{\nu}]) = -\frac{q}{2}\log(2\pi) + \frac{1}{2}\sum_{i=1}^{q}\log(p_i) - \sum_{i=1}^{q}\frac{p_i}{2}(\nu_i^2 + c_i^2 - 2c_i\nu_i),$$

$$\log(P[\omega_i|\boldsymbol{v}^{\boldsymbol{1}}, .., \boldsymbol{v}^{\boldsymbol{q}}]) = \frac{-1}{2}\log(2\pi) + \frac{1}{2}(\sum_{j=1}^{q}\log(v^j_{\boldsymbol{d}^j_i})) - \prod_{j=1}^{q}v^j_{\boldsymbol{d}^j_i}\frac{\omega_i^2}{2},$$

$$\log(P[\upsilon_k^j|\chi_{1j},\chi_{2j}]) = \chi_{1j}\log(\chi_{2j}) - \log(\Gamma(\chi_{1j})) + (\chi_{1j}-1)\log(\upsilon_k^j) - \chi_{2j}\upsilon_k^j,$$

$$\log(P[\chi_{2j}|\zeta_1,\zeta_2]) = \zeta_1\log(\zeta_2) - \log(\Gamma(\zeta_1)) + (\zeta_1-1)\log(\chi_{2j}) - \zeta_2\chi_{2j}.$$

### 1.2.2 Expectation of Natural Parameter Vector $\phi$: w.r.t $Q$

$$< \phi_{b_{ij}} >= \Big(E_u[\boldsymbol{u}_{\alpha_{ij}}(2)](\boldsymbol{f_i^j})^T E_u[\boldsymbol{u_\nu}(1)](\boldsymbol{v_i^j})^T E_u[\boldsymbol{u_\omega}(1)], -\frac{E_u[\boldsymbol{u}_{\alpha_{ij}}(2)]}{2}\Big),$$

$$< \phi_{\boldsymbol{\omega}} >= (\boldsymbol{0}^T, vec(-\frac{1}{2}diag(< \boldsymbol{\delta} >))^T)^T,$$

$$< \delta_i >= \prod_{j=1}^{q} E[\boldsymbol{u}_{\upsilon^j_{\boldsymbol{d}_i^j}}(2)],$$

$$< \boldsymbol{\phi}_{\upsilon_k^j} >= ((\chi_{1j}-1), -E_u[\boldsymbol{u}_{\chi_{2j}}(2)]),$$

$$< \boldsymbol{\phi}_{\chi_{2j}} >= ((\zeta_1-1), -\zeta_2)^T,$$

$$< \phi_{\boldsymbol{\nu}} >= \Big((\boldsymbol{c}\cdot\boldsymbol{p})^T, -\frac{1}{2}diag(\boldsymbol{p})\Big),$$

$$< \boldsymbol{\phi}_{\alpha_{ij}} >= (\gamma_1 - 1, -E[\boldsymbol{u}_{\kappa_j}(2)] \prod_{k:\boldsymbol{C}_{ik}^{\boldsymbol{j}}=1} E_u[\boldsymbol{u}_{a_k}(2)]),$$

$$< \boldsymbol{\phi}_{a_k} >= ((\phi_1-1), -\phi_2),$$

$$< \boldsymbol{\phi}_{\lambda_j} >= ((\lambda_1-1), -E_u[\boldsymbol{u}_{\lambda_2}(2)]),$$

$$< \boldsymbol{\phi}_{\lambda_2} >= ((\rho_1-1), -\rho_2),$$

$$< \boldsymbol{\phi}_{\kappa_j} >= ((\tau_1-1), -E_u[\boldsymbol{u}_{\tau_2}(2)]),$$

$$< \boldsymbol{\phi}_{\tau_2} >= ((\xi_1-1), -\xi_2).$$

### 1.2.3 Expectation of Sufficient Statistic Vector $E[\boldsymbol{u_x}]$ as a Function of $\boldsymbol{\phi_x^*}$ (Messages to Children)

$$E_u[\boldsymbol{u}_{a_k}] = \Big(\psi(\phi^*_{a_k}(1)+1) - \log(-\phi^*_{a_k}(2)), \frac{-\phi^*_{a_k}(1)-1}{\phi^*_{a_k}(2)}\Big),$$

$$E_u[\boldsymbol{u}_{\alpha_{ij}}] = \Big(\psi(\phi^*_{\alpha_{ij}}(1)+1) - \log(-\phi^*_{\alpha_{ij}}(2)), \frac{-\phi^*_{\alpha_{ij}}(1)-1}{\phi^*_{\alpha_{ij}}(2)}\Big),$$

$$E[\boldsymbol{u}_{\lambda_j}] = \Big(\psi(\phi^*_{\lambda_j}(1)+1) - \log(-\phi^*_{\lambda_j}(2)), \frac{-\phi^*_{\lambda_j}(1)-1}{\phi^*_{\lambda_j}(2)}\Big),$$

$$E[\boldsymbol{u}_{\lambda_2}] = \Big(\psi(\phi^*_{\lambda_2}(1)+1) - \log(-\phi^*_{\lambda_2}(2)), \frac{-\phi^*_{\lambda_2}(1)-1}{\phi^*_{\lambda_2}(2)}\Big),$$

$$E[\boldsymbol{u}_{\kappa_j}] = \Big(\psi(\phi^*_{\kappa_j}(1)+1) - \log(-\phi^*_{\kappa_j}(2)), \frac{-\phi^*_{\kappa_j}(1)-1}{\phi^*_{\kappa_j}(2)}\Big),$$

$$E[\boldsymbol{u}_{\tau_2}] = \Big(\psi(\phi^*_{\tau_2}(1)+1) - \log(-\phi^*_{\tau_2}(2)), \frac{-\phi^*_{\tau_2}(1)-1}{\phi^*_{\tau_2}(2)}\Big),$$

$$E[\boldsymbol{u}_{\boldsymbol{b}_j}] = (E[\boldsymbol{u}_{\boldsymbol{b}_j}(1)], E[\boldsymbol{u}_{\boldsymbol{b}_j}(2)]),$$

$$E[\boldsymbol{u}_{\boldsymbol{b}_j}] = (-\boldsymbol{\phi}^*_{\boldsymbol{b_j}}(2)^{-1}\boldsymbol{\phi}^*_{\boldsymbol{b_j}}(1)/2, vec(-\boldsymbol{\phi}^*_{\boldsymbol{b_j}}(2)^{-1}/2 + E[\boldsymbol{u}_{\boldsymbol{b_j}}(1)]E[\boldsymbol{u}_{\boldsymbol{b_j}}(1)]^T)),$$

$$E[\boldsymbol{u}_{\omega}] = (-\boldsymbol{\phi}^*_{\boldsymbol{\omega}}(2)^{-1}\boldsymbol{\phi}^*_{\boldsymbol{\omega}}(1)/2, vec(-\boldsymbol{\phi}^*_{\boldsymbol{\omega}}(2)^{-1}/2 + E[\boldsymbol{u}_{\boldsymbol{\omega}}(1)]E[\boldsymbol{u}_{\boldsymbol{\omega}}(1)]^T)),$$

$$E_u[\boldsymbol{u}_{\chi_{2j}}] = \Big(\psi(\phi^*_{\chi_{2j}}(1)+1) - \log(-\phi^*_{\chi_{2j}}(2)), \frac{-\phi^*_{\chi_{2j}}(1)-1}{\phi^*_{\chi_{2j}}(2)}\Big),$$

$$E_u[\boldsymbol{u}_{\upsilon^j_k}] = \Big(\psi(\phi^*_{\upsilon^j_k}(1)+1) - \log(-\phi^*_{\upsilon^j_k}(2)), \frac{-\phi^*_{\upsilon^j_k}(1)-1}{\phi^*_{\upsilon^j_k}(2)}\Big),$$

$$E[\boldsymbol{u}_{\nu}] = (-\boldsymbol{\phi}^*_{\boldsymbol{\nu}}(2)^{-1}\boldsymbol{\phi}^*_{\boldsymbol{\nu}}(1)/2, vec(-\boldsymbol{\phi}^*_{\boldsymbol{\nu}}(2)^{-1}/2 + E[\boldsymbol{u}_{\boldsymbol{\nu}}(1)]E[\boldsymbol{u}_{\boldsymbol{\nu}}(1)]^T)).$$

### 1.2.4 Messages from Children to Parents

$$< \boldsymbol{\phi}_{\alpha_{ij}a'_k} >= \Big(\gamma_1 \mathbf{1}_{\{k' \in C_{ij}\}}, -E[\boldsymbol{u}_{\kappa_j}(2)]\Big(\prod_{k \neq k' \in C_{ij}} E_u[\boldsymbol{u}_{a_k}(2)]\Big)E_u[\boldsymbol{u}_{\alpha_{ij}}(2)]\Big),$$

$$< \boldsymbol{\phi}_{\alpha_{ij}\kappa_j} >= \Big(\gamma_1, -(\prod_{k \in C_{ij}} E_u[\boldsymbol{u}_{a_k}(2)])E_u[\boldsymbol{u}_{\alpha_{ij}}(2)]\Big),$$

$$< \boldsymbol{\phi}_{\boldsymbol{y}_j\lambda_j} > \;=\; (\frac{n}{2}, \frac{-1}{2}(\boldsymbol{y}_j^T\boldsymbol{y}_j - 2\boldsymbol{y}_j^T\boldsymbol{X}_j E_u[\boldsymbol{u}_{\boldsymbol{b_j}}(1)] + Tr(\boldsymbol{X}_j^T\boldsymbol{X}_j E_u[\boldsymbol{u}_{\boldsymbol{b_j}}(2)])))^T,$$

$$< \boldsymbol{\phi}_{\boldsymbol{y}_j\boldsymbol{b}_j} > \;=\; ((E[\boldsymbol{u}_{\lambda_j}]\boldsymbol{y}_j^T\boldsymbol{X}_j)^T, -(E[\boldsymbol{u}_{\lambda_j}]vec(\boldsymbol{X}_j^T\boldsymbol{X}_j)/2)^T)^T,$$

$$< \boldsymbol{\phi}_{\lambda_j\lambda_2} > \quad = \quad (\lambda_1, -E_u[\boldsymbol{u}_{\lambda_j}(2)]),$$

$$< \boldsymbol{\phi}_{\kappa_j\tau_2} > \quad = \quad (\tau_1, -E_u[\boldsymbol{u}_{\kappa_j}(2)]),$$

$$< \phi_{b_{ij}\boldsymbol{\alpha_{ij}}} >= \Big(\frac{1}{2}, -\frac{1}{2}(E_u[\boldsymbol{u}_{\boldsymbol{b_j}}(2)]_{ii} + (\boldsymbol{f_{ij}}^T E_u[\boldsymbol{u}_\nu(2)]\boldsymbol{f_{ij}}\boldsymbol{v_{ij}}^T E_u[\boldsymbol{u}_\omega(2)]\boldsymbol{v_{ij}}, \\ - 2E_u[\boldsymbol{u}_{\boldsymbol{b_j}}(1)]_i \boldsymbol{v_{ij}}^T E_u[\boldsymbol{u}_\omega(1)]\boldsymbol{f_{ij}}^T E_u[\boldsymbol{u}_\nu(1)]))\Big),$$

$$< \phi_{b_{ij}\boldsymbol{\omega}} >= \Big(E_u[\boldsymbol{u}_{\alpha_{ij}}(2)]E_u[\boldsymbol{u}_{\boldsymbol{b_j}}(1)]_i \boldsymbol{f_{ij}}^T E_u[\boldsymbol{u}_\nu(1)]\boldsymbol{v_{ij}}^T, \\ (-\frac{E_u[\boldsymbol{u}_{\alpha_{ij}}(2)]\boldsymbol{f_{ij}}^T E_u[\boldsymbol{u}_\nu(2)]\boldsymbol{f_{ij}}}{2} vec(\boldsymbol{v_{ij}}\boldsymbol{v_{ij}}^T))^T\Big),$$

$$< \phi_{b_{ij}\boldsymbol{\nu}} >= \Big(E_u[\boldsymbol{u}_{\alpha_{ij}}(2)]E_u[\boldsymbol{u}_{\boldsymbol{b_j}}(1)]_i \boldsymbol{v_{ij}}^T E_u[\boldsymbol{u}_\omega(1)]\boldsymbol{f_{ij}}^T, \\ (-\frac{E_u[\boldsymbol{u}_{\alpha_{ij}}(2)]\boldsymbol{v_{ij}}^T E_u[\boldsymbol{u}_\omega(2)]\boldsymbol{v_{ij}}}{2} vec(\boldsymbol{f_{ij}}\boldsymbol{f_{ij}}^T))^T\Big),$$

$$< \boldsymbol{\phi}_{\omega_i \upsilon_k^j} >= \mathbf{1}_{\{k=\boldsymbol{d}_i^j\}}\Big(\frac{1}{2}, -\Big(\prod_{j'\neq j} E[\boldsymbol{u}_{\upsilon^{j'}_{\boldsymbol{d}_i^{j'}}}(2)]\Big)\frac{E[\boldsymbol{u}_\omega(2)]_{ii}}{2}\Big),$$

$$< \boldsymbol{\phi}_{\upsilon_k^j \chi_{2j}} > \quad = \quad (\chi_{1j}, -E_u[\upsilon_k^j(2)]).$$

### 1.2.5 Approximating -$\phi_{\boldsymbol{b}}^*(2)$ for Fast Inversion

One time critical step when performing the updates is the inversion of $\boldsymbol{\phi}_{\boldsymbol{b}}^*(2)$ which has to be done for each round and for each gene.

$$\begin{aligned} -\boldsymbol{\phi}_{\boldsymbol{b}}^*(2) &= E[\boldsymbol{u}_\lambda(2)](\boldsymbol{X}^T\boldsymbol{X})/2) + \frac{1}{2}diag(E[\boldsymbol{u}_{\alpha_i}(2)]), \\ &= c\boldsymbol{X}^T\boldsymbol{X} + \boldsymbol{D}_{\boldsymbol{\alpha}}, \end{aligned}$$

where $c$ is a scalar and $\boldsymbol{D}_{\boldsymbol{\alpha}}$ is a diagonal matrix (gene-wise indices were dropped). Because of LD and data on fewer samples than SNPs in the gene region, $\boldsymbol{X}^T\boldsymbol{X}$ is degenerate or close to degeneracy and we can approximate it with a low rank matrix. Set $\boldsymbol{X} = \boldsymbol{U}\boldsymbol{D}\boldsymbol{V}^T$ as the singular value decomposition. Then, set $\boldsymbol{U_t}$ to consist of the first $t$ columns and $\boldsymbol{D_t}$ to consist of the

upper left $t \times t$ submatrix of $\boldsymbol{D}$. $t$ is set such that the sum of squares of $\boldsymbol{D_t}$ is close to the sum of squares of $\boldsymbol{D}$ (say 99%). Set $\boldsymbol{A_t} = \sqrt{c}\boldsymbol{U_t D_t}$ so that

$$c\boldsymbol{X^T X} + \boldsymbol{D_\alpha} \quad \approx \quad \boldsymbol{A}_t \boldsymbol{A}_t^{\boldsymbol{T}} + \boldsymbol{D_\alpha}.$$

Using the Woodbury matrix identity, we have

$$(\boldsymbol{A}_t \boldsymbol{A}_t^{\boldsymbol{T}} + \boldsymbol{D_\alpha})^{-1} \quad = \quad \boldsymbol{D_\alpha}^{-1} - \boldsymbol{D_\alpha}^{-1} \boldsymbol{A}_t (\boldsymbol{I_t} + \boldsymbol{A}_t^{\boldsymbol{T}} \boldsymbol{D_\alpha}^{-1} \boldsymbol{A}_t)^{-1} \boldsymbol{A}_t^{\boldsymbol{T}} \boldsymbol{D_\alpha}^{-1}.$$

Since $\boldsymbol{D_\alpha}$ is diagonal it is easy to invert and $(\boldsymbol{I_t} + \boldsymbol{A}_t^{\boldsymbol{T}} \boldsymbol{D_\alpha}^{-1} \boldsymbol{A}_t)$ is of dimension $t$ and can be inverted much faster than the full matrix. This approach builds on the fact that the matrix to invert is well conditioned if $\boldsymbol{D_\alpha}$ is large enough (relative to the diagonal of $c\boldsymbol{X}^T\boldsymbol{X}$), i.e.: if the regression is regularized sufficiently via the priors on $\boldsymbol{b}$ and $\lambda$. We can also add the discarded variance onto the diagonal. Set $t^c$ as the indices larger than $t$ and $\boldsymbol{A_{t^c}} = \sqrt{c}\boldsymbol{U_{t^c} D_{t^c}}$. Then $w = \sum_{ij}([\boldsymbol{A}_{t^c}]_{ij})^2$ is the the total variance discarded. We set $\boldsymbol{D'_\alpha} = \boldsymbol{D_\alpha} + \boldsymbol{I}_n \frac{w}{n}$, where $n$ is the dimension of $\boldsymbol{D_\alpha}$

$$(c\boldsymbol{X}^T\boldsymbol{X} + \boldsymbol{D_\alpha})^{-1} \quad \approx \quad \boldsymbol{D'_\alpha}^{-1} - \boldsymbol{D'_\alpha}^{-1} \boldsymbol{A}_t (\boldsymbol{I_t} + \boldsymbol{A}_t^{\boldsymbol{T}} \boldsymbol{D'_\alpha}^{-1} \boldsymbol{A}_t)^{-1} \boldsymbol{A}_t^{\boldsymbol{T}} \boldsymbol{D'}_{\boldsymbol{\alpha}}^{\boldsymbol{-1}}.$$

Additionally to the inversion, we would like to speed up the determinant calculation step used to calculate the lower bound. We can make use of the matrix determinant lemma i.e.

$$\det(\boldsymbol{D'}_\alpha + \boldsymbol{A}_t \boldsymbol{A}_t^{\boldsymbol{T}}) = \det(\boldsymbol{I_t} + \boldsymbol{A}_t^{\boldsymbol{T}} \boldsymbol{D'}_{\boldsymbol{\alpha}}^{\boldsymbol{-1}} \boldsymbol{A}_t) \det(\boldsymbol{D'}_\alpha).$$

### 1.2.6 Working with Summary Statistics.

As we mentioned above, we can approximate $\boldsymbol{X}^T\boldsymbol{X}$. We can also estimate it externally from 1000 Genomes, with $\boldsymbol{\Sigma}$ say, and approximate $\boldsymbol{X^T y}$ with summary statistics $\sqrt{n}\boldsymbol{z}$. However, this can lead to convergence problems as the modeling assumptions do not hold anymore. For limited $n$ and arbitrary but fixed $\boldsymbol{X}^T\boldsymbol{X}$ and $\boldsymbol{X^T y}$, there is not always an adequate $\boldsymbol{X}$ and $\boldsymbol{y}$ fullfilling

the equations. We therefore reformulate the model in terms of summary statistics such that for each SNP-gene pair the statistics $\boldsymbol{z}_j$ are observed: Since we have

$$\frac{1}{\sqrt{n}}\boldsymbol{X}_j^T\boldsymbol{y}_j = \frac{1}{\sqrt{n}}\boldsymbol{X}_j^T\boldsymbol{X}_j\boldsymbol{b}_j + \frac{1}{\sqrt{n}}\boldsymbol{X}_j^T\boldsymbol{\epsilon}_j.$$

We can naturally model the vector of summary statistics as

$$\boldsymbol{z}_j = \boldsymbol{\Sigma}_j\sqrt{n}\boldsymbol{b}_j + \boldsymbol{\epsilon}_j',$$

where $\boldsymbol{\epsilon}_j' \sim N_m(\boldsymbol{0}, \lambda_j^{-1}\boldsymbol{\Sigma}_j)$.

We then have

$$\log(P[\boldsymbol{z}_j|\boldsymbol{b}_j]) = -\frac{m_j}{2}\log(2\pi) - \frac{1}{2}\log(\det(\frac{1}{\lambda_j}\boldsymbol{\Sigma}_j)) - \frac{\lambda_j}{2}(\boldsymbol{z}_j^T\boldsymbol{\Sigma}_j^{-1}\boldsymbol{z}_j - 2\sqrt{n}\boldsymbol{z}_j^T\boldsymbol{b}_j + n\boldsymbol{b}_j'^T\boldsymbol{\Sigma}_j\boldsymbol{b}_j).$$

If $\boldsymbol{\Sigma}_j$ is not full rank we could use the pseudo-inverse. Note however, that in practice, regularization is needed as otherwise $E[\boldsymbol{z}]$ will most likely not lie in the vector space spanned by the eigenvectors of $\boldsymbol{\Sigma}_j$.If $\boldsymbol{\Sigma_j}$ has rank $n$, we have,

$$\boldsymbol{\phi}_{\boldsymbol{z}_j\boldsymbol{b}_j} \quad = \quad ((\sqrt{n}\lambda_j\boldsymbol{z}_j), -(n\lambda_j vec(\boldsymbol{\Sigma}_j)/2)^T)^T.$$

Next, we have

$$\boldsymbol{\phi}_{\boldsymbol{z}_j\lambda_j} \quad = \quad (\frac{m_j}{2}), -\frac{1}{2}(\boldsymbol{z}_j^T\boldsymbol{\Sigma}_j^{-1}\boldsymbol{z}_j - 2\sqrt{n}\boldsymbol{z}_j^T\boldsymbol{b}_j + n\boldsymbol{b}_j{}^T\boldsymbol{\Sigma}_j\boldsymbol{b}_j).$$

Importantly, if $\boldsymbol{\Sigma}_j$ is full rank, the right hand term is now guaranteed to be non-positive which could not be guaranteed before the reformulation.

### 1.2.7 Computing the Lower Bound of the Data Log-Evidence

Variational message passing can additionally provide a lower bound $L(Q)$ on the data log-evidence $\log(P[V])$, where the gap is exactly the KL divergence. To compute the $L(Q)$ the algorithm needs to additionally compute $< g(\boldsymbol{\phi}_{\boldsymbol{x}}(\boldsymbol{pa}_{\boldsymbol{x}})) >$, $g(\boldsymbol{\phi^*}_{\boldsymbol{x}})$ and $\log(P[\boldsymbol{V}|\boldsymbol{H}])$. For the *BAGEA*

model, the required quantities of the form $< g(\boldsymbol{\phi_x}(\boldsymbol{pa_x})) >$ are:

$$< g_{b_{ij}}(pa) >= \frac{1}{2}E[\boldsymbol{u}_{\alpha_{ij}}(1)] - \frac{E[\boldsymbol{u}_{\alpha_{ij}}(2)]}{2}Tr(\boldsymbol{v_{ij}}\boldsymbol{v_{ij}}^T E[\boldsymbol{u_\omega}(2)])Tr(\boldsymbol{f_{ij}}\boldsymbol{f_{ij}}^T E[\boldsymbol{u_\nu}(2)]),$$

$$< g_{\lambda_j}(pa) >= \lambda_1 E[\boldsymbol{u}_{\lambda_2}(1)] - \log(\Gamma(\lambda_1)),$$

$$< g_{\lambda_2}(pa) >= \rho_1 \log(\rho_2) - \log(\Gamma(\rho_1)),$$

$$< g_{\alpha_i}(pa) >= \gamma_1\Big(E[\boldsymbol{u}_{\kappa_j}(1)] + \sum_{k:\boldsymbol{C}^{\boldsymbol{j}}_{ik}=1} E[\boldsymbol{u}_{a_k}(1)]\Big) - \log(\Gamma(\gamma_1)),$$

$$< g_{\kappa_j}(pa) >= \tau_1 E[\boldsymbol{u}_{\tau_2}(1)] - \log(\Gamma(\tau_1)),$$

$$< g_{\tau_2}(pa) >= \xi_1 \log(\xi_2) - \log(\Gamma(\xi_1)),$$

$$< g_{a_k}(pa) >= \phi_1 \log(\phi_2) - \log(\Gamma(\phi_1)),$$

$$< g_{\omega_i}(pa) >= \frac{-1}{2}\log(2\pi) + \frac{1}{2}(\sum_{j=1}^{q} E[\boldsymbol{u}_{\upsilon^j_{\boldsymbol{d}^j_i}}(1)]),$$

$$< g_{\upsilon^j_k}(pa) >= \chi_{1j} E_u[\boldsymbol{u}_{\chi_{2j}}(1)] - \log(\Gamma(\chi_{1j})),$$

$$< g_{\boldsymbol{\nu}}(pa) >= \frac{-q}{2}\log(2\pi) + \frac{1}{2}\sum_{i=1}^{q}\log(p_i) - \sum_{i=1}^{q}\frac{p_i c_i^2}{2},$$

$$< g_{\chi_{wj}}(pa) >= \zeta_1 \log(\zeta_2) - \log(\Gamma(\zeta_1)).$$

the required quantities of the form $g(\boldsymbol{\phi^*_x})$ are:

$$g(\boldsymbol{\phi}^*_{\boldsymbol{b}_j}) = -\frac{m_j}{2}\log(2\pi) + \frac{1}{2}\log(\det(-2(\boldsymbol{\phi}^*_{\boldsymbol{b}_j}(2)))) + \frac{1}{4}(\boldsymbol{\phi}^*_{\boldsymbol{b}_j}(1))(\boldsymbol{\phi}^*_{\boldsymbol{b}_j}(2))^{-1}(\boldsymbol{\phi}^*_{\boldsymbol{b}_j}(1))^T,$$

$$g(\boldsymbol{\phi}^*_{\boldsymbol{\omega}}) = -\frac{s}{2}\log(2\pi) + \frac{1}{2}\log(\det(-2(\boldsymbol{\phi}^*_{\boldsymbol{\omega}}(2)))) + \frac{1}{4}(\boldsymbol{\phi}^*_{\boldsymbol{\omega}}(1))(\boldsymbol{\phi}^*_{\boldsymbol{\omega}}(2))^{-1}(\boldsymbol{\phi}^*_{\boldsymbol{\omega}}(1))^T,$$

$$g(\boldsymbol{\phi}^*_{\boldsymbol{\nu}}) = -\frac{q}{2}\log(2\pi) + \frac{1}{2}\log(\det(-2(\boldsymbol{\phi}^*_{\boldsymbol{\nu}}(2)))) + \frac{1}{4}(\boldsymbol{\phi}^*_{\boldsymbol{\nu}}(1))(\boldsymbol{\phi}^*_{\boldsymbol{\nu}}(2))^{-1}(\boldsymbol{\phi}^*_{\boldsymbol{\nu}}(1))^T,$$

$$g(\boldsymbol{\phi}^*_{\upsilon^j_k}) = (\phi^*_{\upsilon^j_k}(1)+1)\log(-\phi^*_{\upsilon^j_k}(2)) - \log(\Gamma(\phi^*_{\upsilon^j_k}(1)+1)),$$

$$g(\boldsymbol{\phi}^*_{\tau_2}) = (\phi^*_{\tau_2}(1)+1)\log(-\phi^*_{\tau_2}(2)) - \log(\Gamma(\phi^*_{\tau_2}(1)+1)),$$

$$g_{a_k}(\boldsymbol{\phi}^*_{a_k}) = (\boldsymbol{\phi}^*_{a_k}(1)+1)\log(-\boldsymbol{\phi}^*_{a_k}(2)) - \log(\Gamma(\boldsymbol{\phi}^*_{a_k}(1)+1)),$$

$$g_{\alpha_i}(\boldsymbol{\phi}^*_{\alpha_i}) = (\boldsymbol{\phi}^*_{\alpha_i}(1)+1)(\log(-\boldsymbol{\phi}^*_{\alpha_i}(2))) - \log(\Gamma(\boldsymbol{\phi}^*_{\alpha_i}(1)+1)),$$

$$g(\boldsymbol{\phi}^*_{\lambda_j}) = (\phi^*_{\lambda_j}(1)+1)\log(-\phi^*_{\lambda_j}(2)) - \log(\Gamma(\phi^*_{\lambda_j}(1)+1)),$$

$$g(\boldsymbol{\phi}^*_{\lambda_2}) = (\phi^*_{\lambda_2}(1)+1)\log(-\phi^*_{\lambda_2}(2)) - \log(\Gamma(\phi^*_{\lambda_2}(1)+1)),$$

$$g(\boldsymbol{\phi}^*_{\kappa_j}) = (\phi^*_{\kappa_j}(1)+1)\log(-\phi^*_{\kappa_j}(2)) - \log(\Gamma(\phi^*_{\kappa_j}(1)+1)),$$

$$g(\boldsymbol{\phi}^*_{\chi_{2j}}) = (\phi^*_{\chi_{2j}}(1)+1)\log(-\phi^*_{\chi_{2j}}(2)) - \log(\Gamma(\phi^*_{\chi_{2j}}(1)+1)).$$

We further need the terms of the form $< \log(P[\boldsymbol{V}|\boldsymbol{H}]) >$. For the individiual level data, we have

$$L_{\boldsymbol{y}_j} = -\frac{n_j}{2}\log(2\pi) + \frac{n_j}{2}E[\boldsymbol{u}_{\lambda_j}(1)] - \frac{E[\boldsymbol{u}_{\lambda_j}(2)]}{2}(\boldsymbol{y}_j^T\boldsymbol{y}_j - 2\boldsymbol{y}_j^T\boldsymbol{X}_jE[\boldsymbol{u}_{b_j}(1)] + Tr(\boldsymbol{X}_j^T\boldsymbol{X}_j \cdot E[\boldsymbol{u}_{b_j}(2)])).$$

For the summary statistics observables we have

$$\begin{aligned} L_{\boldsymbol{z}_j} &= -\frac{m_j}{2}\log(2\pi) + \frac{m_j}{2}E[\boldsymbol{u}_{\lambda_j}(1)] - \frac{1}{2}\log(\det(\boldsymbol{\Sigma}_j)) \\ &- \frac{E[\boldsymbol{u}_{\lambda_j}(2)]}{2}(\boldsymbol{z}_j^T\boldsymbol{\Sigma}_j^{-1}\boldsymbol{z}_j - 2\sqrt{n_j}\boldsymbol{z}_j^TE[\boldsymbol{u}_{\boldsymbol{b}_j}(1)] + n_jTr(\boldsymbol{\Sigma}_jE[\boldsymbol{u}_{\boldsymbol{b}_j}(2)])). \end{aligned}$$

## 1.3 Default Parameter Settings

Default parameter settings were set to $\gamma_1 = 3$, $\tau_1 = 2$, $\phi_1 = 3$, $\phi_2 = 3$, $\xi_1 = 100$, $\xi_2 = 0.03$, $\lambda_1 = \sqrt{10^5}$, $\rho_1 = 10^5$, $\rho_2 = \sqrt{10^5}$, $p = 5$,$\chi_1 = 1$, $\chi_2 = 3 \cdot 10^{-4}$, $\zeta_1 = 1$, $\zeta_2 = 100$, $\boldsymbol{p} = \mathbf{0.2}$ and $\boldsymbol{c} = \mathbf{0}$.

## 1.4 ExPecto Comparison

To derive expression effects of genetic variants using the ExPecto approach directly, we used already trained models that predict tissue-specific expression effects from the epigenetic marks. These models were trained using an $l_2$-boosting approach for the GTEx datasets as part of the release of ExPecto. We used 13 GTEx datasets, as LCL results were excluded due to the combination with GEAUVADIS dataset. As a further input to ExPecto, we used the predicted

functional impacts on epigenetic marks for all common SNPs, that were also used as input for *BAGEA*. We mapped SNPs uniquely to its closest gene as recommended for ExPecto (See ExPecto github page). ExPecto then yields for each variant its predicted impact on gene expression in *cis*. For each SNP, its effect was scaled based the variant's allele frequency as estimated from the 1KG European samples, i.e. Each effect was multiplied by this variant's standard deviation transforming the effect sizes to the values in a model, where the genotype matrix was normalized. This yielded $\hat{\boldsymbol{b}}_{\boldsymbol{j}}^{\boldsymbol{ExP}}$, the effect size vector for gene $j$. We want to calculate the scalar product between the phenotype vector $\boldsymbol{y}_j$ for gene $j$ and the estimated directed predictor $\hat{\boldsymbol{\mu}}_j = \boldsymbol{X}_j \hat{\boldsymbol{b}}_{\boldsymbol{j}}^{\boldsymbol{ExP}}$. Using $\boldsymbol{y}_j^T \boldsymbol{X}_j / \sqrt{n} = \boldsymbol{z}_j$, we get $\boldsymbol{y}_j^T \hat{\boldsymbol{\mu}}_j = \boldsymbol{z}_j^T \hat{\boldsymbol{b}}_{\boldsymbol{j}}^{\boldsymbol{ExP}} \sqrt{n}$. To estimate the on predictor size $S_j^{ExP}$, we use external LD matrix $\boldsymbol{\Sigma}_j$ derived from 1KG European samples because $S_j^{ExP} = (\hat{\boldsymbol{b}}_{\boldsymbol{j}}^{\boldsymbol{ExP}})^T \boldsymbol{X}_j^T \boldsymbol{X}_j \hat{\boldsymbol{b}}_{\boldsymbol{j}}^{\boldsymbol{ExP}} = (\hat{\boldsymbol{b}}_{\boldsymbol{j}}^{\boldsymbol{ExP}})^T \boldsymbol{\Sigma}_j \hat{\boldsymbol{b}}_{\boldsymbol{j}}^{\boldsymbol{ExP}} n$. We calculated the corresponding values for *BAGEA* in an analogous fashion.

## 1.5 Simulation

We simulated SNP-wise z-scores according to the *BAGEA* model using the genoypes in the european 1KG sample. As directed annotation, we used the *Blood* annotation subset, comprising 253 annotations. For each simulation setting, we set a certain number of effect sizes $\omega_i$ to non-zero (see below for how these effect sizes were chosen). We set the undirected weight vector $\boldsymbol{\nu}$ and $\boldsymbol{a}$, as well as the hyperparameters $\lambda_2$ and $\tau_2$ to the same value as was observed when fitting the monocyte data using the *Blood* annotation subset (See e.g. Figure 2D). Hidden variables other than $\boldsymbol{\nu}$, $\boldsymbol{\omega}$, $\boldsymbol{a}$ $\lambda_2$ and $\tau_2$ were simulated according to the default hyper-parameter settings. For the different simulations, we varied how to set $\boldsymbol{\omega}$. We had two strategies to choose annotations with non-zero effects: In the *unstructured* setting, effects were chosen at random independently from each other. The number of non-zero effects were varied from 3 to 12. In the *structured* setting, we chose a certain number of cell types (1 to 4 cell lines) and set all effects for the annotations *H3K27ac*, *H3K4me1* and *DNase.all.peaks* to non-zero. (cell types for which not all 3

annotations were availabe, were excluded). Each non-zero annotation effect was set to $\sqrt{0.014/n}$ were $n$ was the number of non-zero effects for a given setting. We chose this value because fitting the monocyte data to the *Blood* annotation subset yielded $\hat{\boldsymbol{\omega}}^T\hat{\boldsymbol{\omega}} = 0.014$. The training set was comprised of genes on chromosomes 1 and 2 and test set was comprised of genes on chromosome 3.

### 1.6 Estimating $E[b_{ij}^2]$ Conditional on Global *BAEGA* Parameters.

While *BAGEA* is designed to build predictors of gene expression based on directed and undirected annotations, it can also be used to predict which SNPs likely have an effect irrespective of directionality. A natural strategy to do so is to estimate the expected squared effect of SNP $i$ on gene $j$ conditional on global parameter estimates, i.e. estimate $E[b_{ij}^2|\boldsymbol{G}]$, where $\boldsymbol{G}$, represents all global *BAGEA* parameter estimates (the term global parameters refer to parameters that are not specific to a gene or SNP such as, for instance, $\boldsymbol{\omega}$ or $\boldsymbol{\nu}$).

$$E[b_{ij}^2|\boldsymbol{G}] = E[E[b_{ij}^2|\alpha_i, \boldsymbol{G}]|\boldsymbol{G}], \qquad (-22)$$

where the law of total expectation was used. We then have

$$E[b_{ij}^2|\alpha_{ij}, \boldsymbol{G}] = Var(b_{ji}|\alpha_{ij}, \boldsymbol{G}) + E[b_{ij}|\boldsymbol{G}]^2. \qquad (-22)$$

With

$$Var(b_{ji}|\alpha_{ij}, \boldsymbol{G}) = \alpha_{ij}^{-1}, \qquad (-22)$$

and

$$E[\alpha_{ij}^{-1}|\boldsymbol{G}] = E[E[\alpha_{ij}^{-1}|\kappa_j, \boldsymbol{G}]|\boldsymbol{G}], \qquad (-22)$$

then

$$E[\alpha_{ij}^{-1}|\kappa_j, \boldsymbol{G}] = \frac{\kappa_j\gamma_{ij}}{\gamma_1 - 1}, \qquad (-22)$$

and

$$E[\kappa_j|\boldsymbol{G}] = \frac{\tau_1}{\tau_2}. \tag{-22}$$

Putting it all together yields

$$E[\hat{b^2_{ij}}|\boldsymbol{G}] = \prod_{k:\boldsymbol{C}^{\boldsymbol{j}}_{ik}=1} \hat{a}_k \frac{\tau_1}{\hat{\tau}_2(\gamma_1 - 1)} + [(\boldsymbol{F^j}\hat{\boldsymbol{\nu}}) \cdot (\boldsymbol{V^j}\hat{\boldsymbol{\omega}})]^2_i. \tag{-22}$$

## 1.7 Torus Comparison

We prepared undirected annotation matrices from the same annotation matrices as were used for *BAGEA*. We used the *TF*-subset of annotation using the absolute value of the directed annotation values as input annotation value, i.e. $|\boldsymbol{V^j}|$. We concatenated the resulting matrix with the undirected annotation matrix $|\boldsymbol{C^j}|$ related to distance to *TSS*. GTEx z-scores, for 13 GTEx experiments (excluding LCL) were selected s.t. all SNP gene pairs were used that were also used when fitting *BAGEA*. We fitted *Torus* using data for chromosomes 1 to 15 for various values of the penalty parameters $l_2$ (0.05, 0.1, 0.5, 1, 5, 10, 20, 50, 100, 500), ignoring results when there were convergence issues (probably due to insufficient regularization as it tended to happen primarily for the lowest values for $l_2$. of 130 separate settings, 113 did converge). We evaluated performance on chromosomes 16 to 22 for fixed settings of $l_2$ as well as via the *overfit* strategy, where for each GTEx dataset, the $l_2$ setting was chosen that performed best on the test chromosomes 16 to 22. This was done to yield an upper bound on what could be potentially expected with a more refined $l_2$ parameter selection strategy.
