# Supplementary figures and images for: A framework for integrating directed and undirected annotations to build explanatory models of cis-eQTL data
Source: PLoS Comput Biol. 2020 Jun 9;16(6):e1007770. doi: 10.1371/journal.pcbi.1007770 (PMC7332077; doi:10.1371/journal.pcbi.1007770)

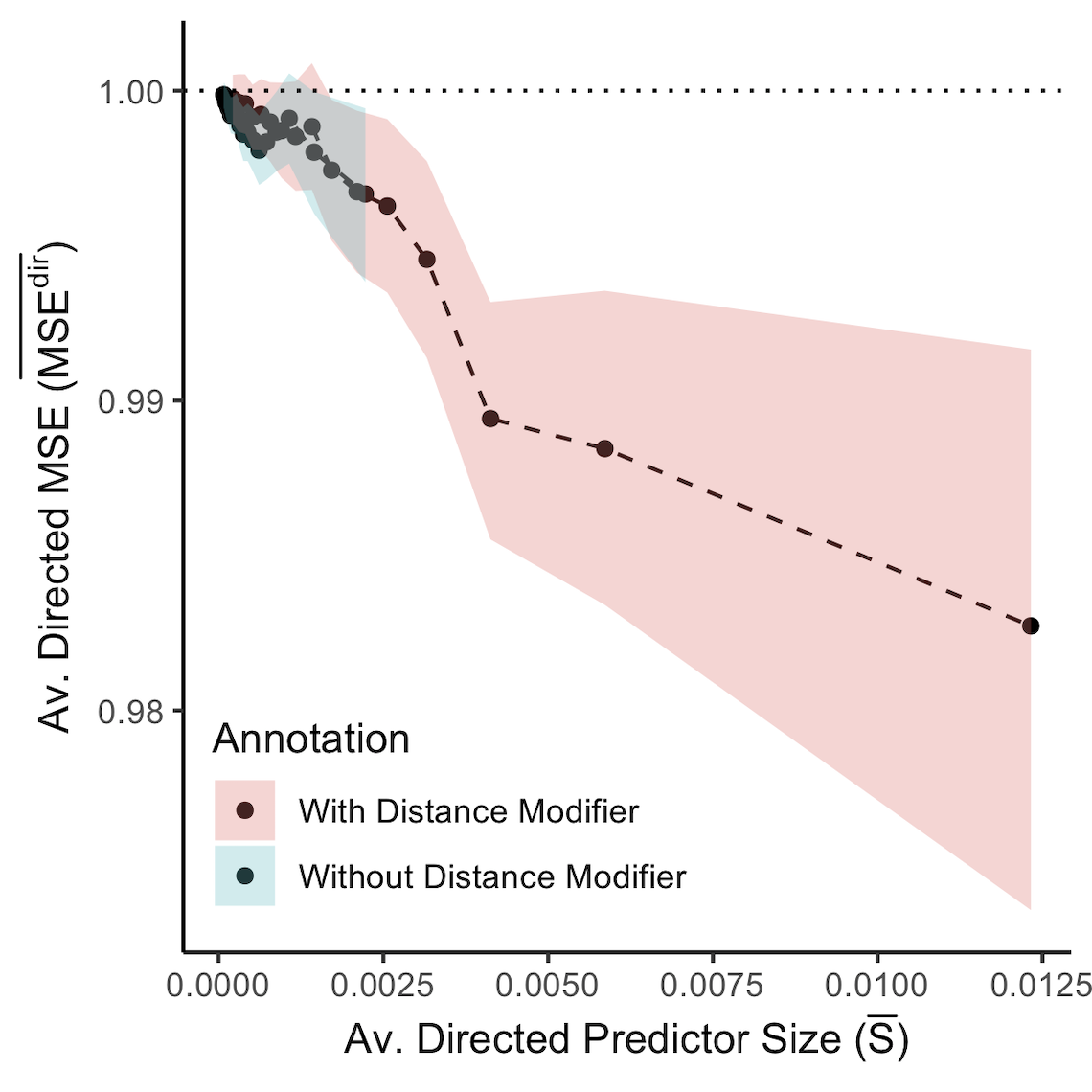

Supplement: S1 Fig — We used BAGEA to predict gene expresion for CD14 positive monocytes using the Blood annotation set, analogously to Fig 2 but removing the distance modifier by constraining all elements of ν^ except the intercept element to zero (Without Distance Modifier). For comparison, we additionally plotted results achieved with the same data and settings except using the default prior for ν (With Distance Modifier). We see substantial decrease in power when the distance dependence of the effect sizes is not modeled. (TIFF) [file pcbi.1007770.s002.tiff]

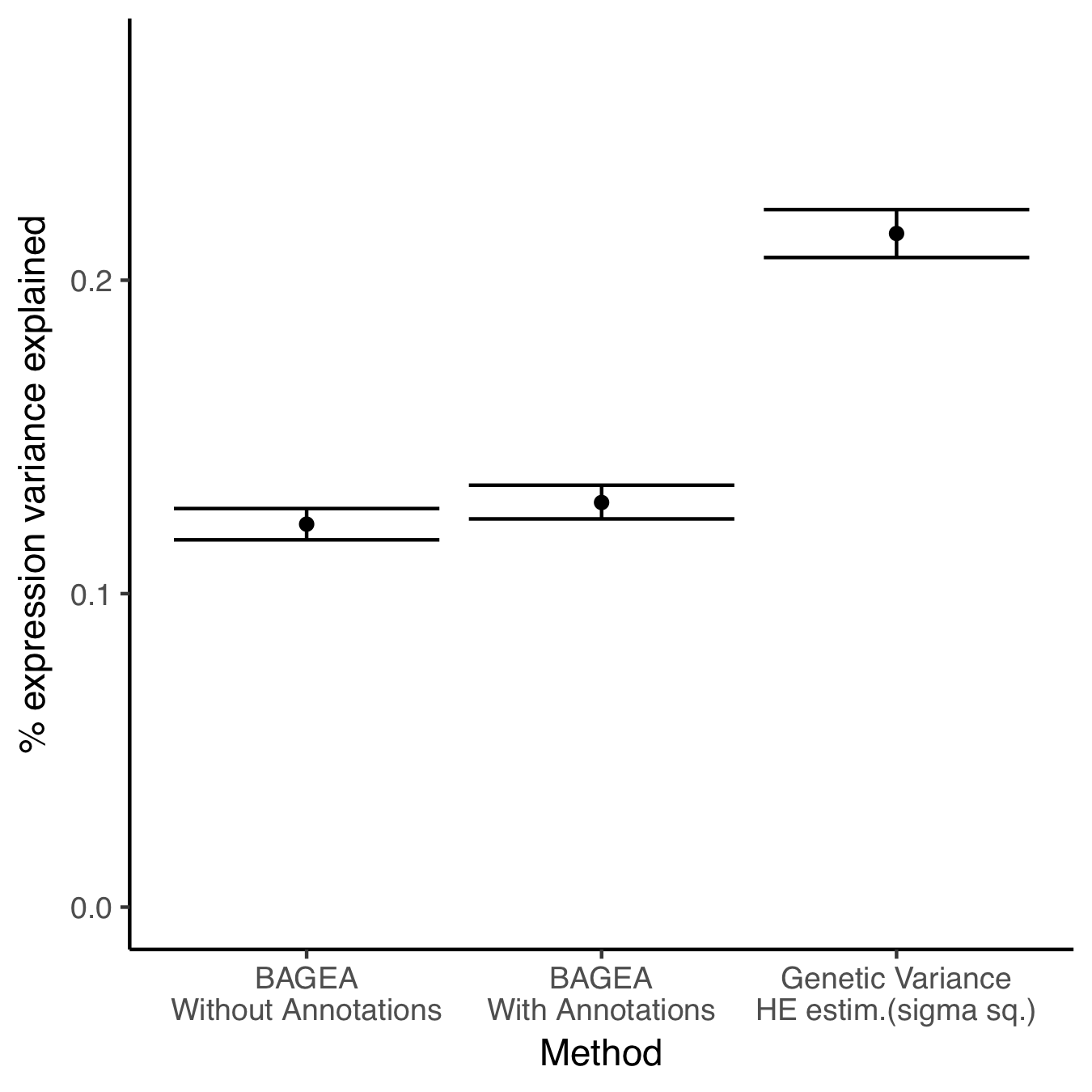

Supplement: S2 Fig — SNP effect size estimates b^j were derived from a subsample (134 individuals) of one dataset [20]. These estimates were used to predict gene expression in the other available monocyte samples [20] [21]. Fits were performed for genes on chromosomes 1 to 22 that had at least a marginal eQTL p-value of 10−10 or below in the combined data. Shown is the average estimated expression variance explained in the test data using the Blood annotation subset and default distance annotations. BAGEA was run either with the default parameter setting (BAGEA With Annotations), or with annotation uniformed setting where a was constrained close to 1 and ω was constrained close to 0 (BAGEA Without Annotations). Additionally, we compared those estimates to estimates of average genetic variance explained in cis as estimated by Haseman-Elston regression on the test data. 95% confidence intervals were derived by bootstrap sampling genes. We see that 60% of estimated genetic variance in cis is explained by BAGEA out-of-sample estimates of bj^. Further, running BAGEA in annotation uninformed mode dropped this fraction to 0.567%. Overall, we saw that 62.5% of assayed genes had a lower MSE in the annotation informed model than in the annotation uninformed model. (TIFF) [file pcbi.1007770.s003.tiff]

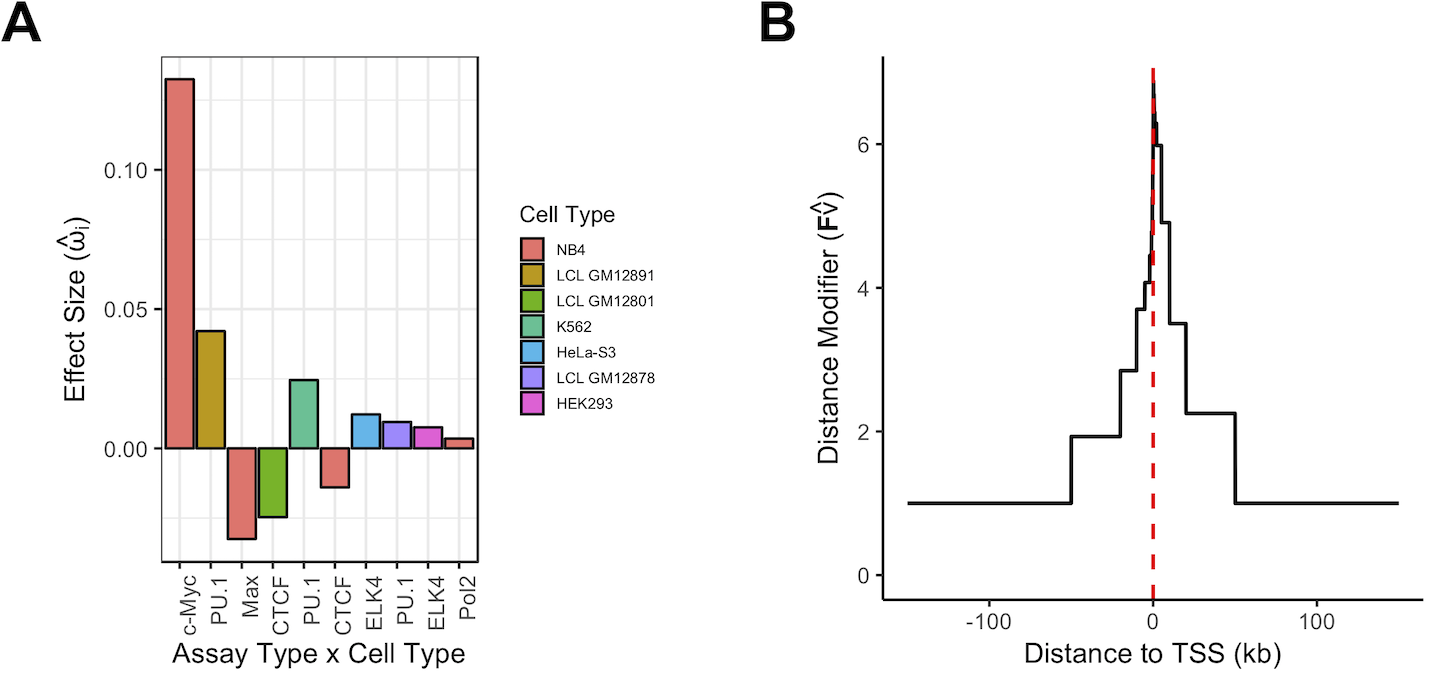

Supplement: S3 Fig — Shown are parameter estimates from fitting monocyte eQTL data using TF ExPecto predictions in all cell types. (A) BAGEA reveals the experiments underlying the directed annotations that are most predictive of gene expression. Assay Type x Cell Type: Each experiment is a particular assay type performed in a particular cell type. Effect Size (ω^i, for experiment i): The BAGEA-estimated effect on gene expression. Shown here the ten largest directed annotation effect sizes. We see c-Myc annotation in NB4 dominates. (B) Shown is the estimated distance modifier of the directed component, Fν^. We see a characteristic peak around the TSS, implying that the directed annotations are upweighted close to the TSS. (TIFF) [file pcbi.1007770.s004.tiff]

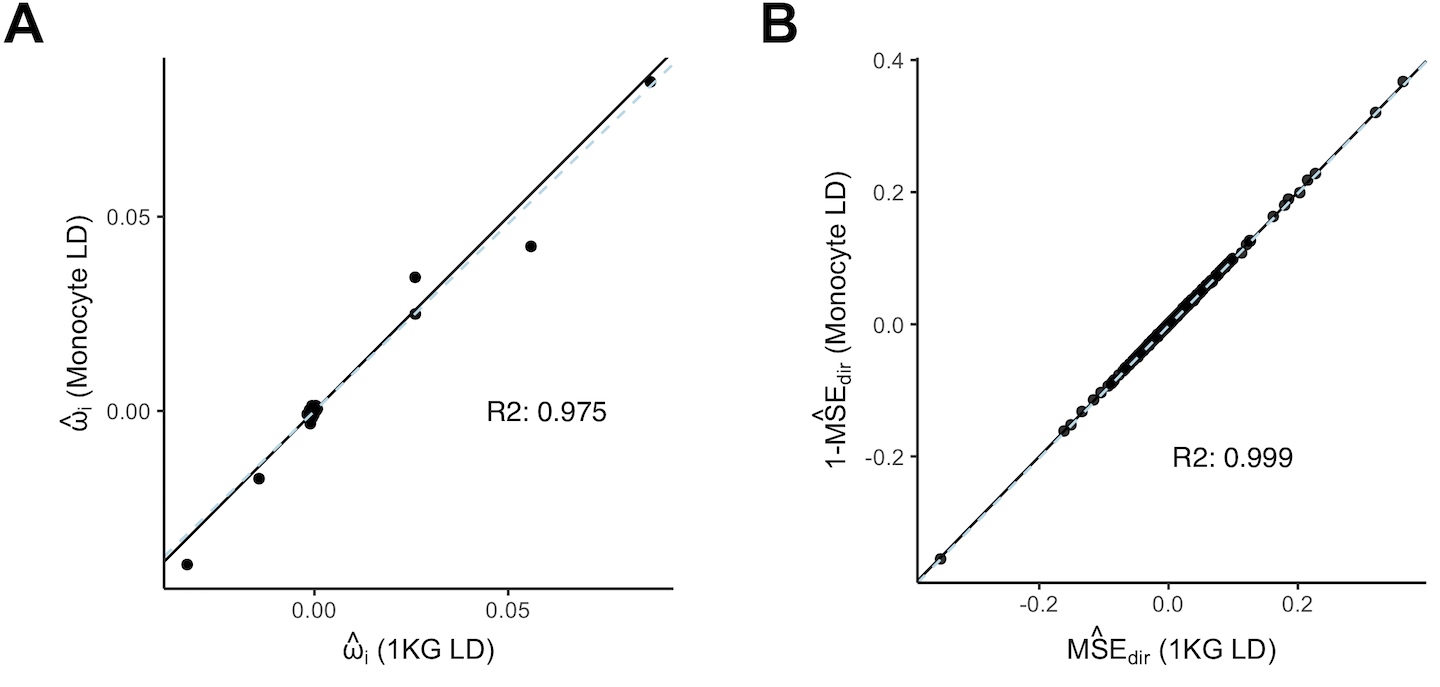

Supplement: S4 Fig — (A) Shown is a comparison of estimates of the directed annotation effect vector ω when using external reference LD information or individual level genotypes. We retrained BAGEA with the blood monocyte summary statistics using reference LD matrices from the 1000 Genomes Project (1KG). ω^i (1KG): Directed annotation effect, measured as ω estimates from BAGEA using 1KG reference LD information. ω^i (Monocyte LD): Directed annotation effect, measured as ω estimates from BAGEA using individual-level genotypes from the monocyte data itself (i.e. using the same genotypes as for the deriving the summary statistics). (B) To investigate the extent to which MSEjdir can be approximated using summary statistics and reference 1KG LD matrices, we calculated MSEjdir on chromosomes 16 to 22 from summary statistics of monocyte cis-eQTLs (see formula in main text). We then compared these to the original MSEjdir values that were computed using genotypes of the monocyte datasets. The same SNPs were used in both calculations. R2: The coefficient of determination, measuring goodness-of-fit, from a linear regression of the data shown. (TIFF) [file pcbi.1007770.s005.tiff]

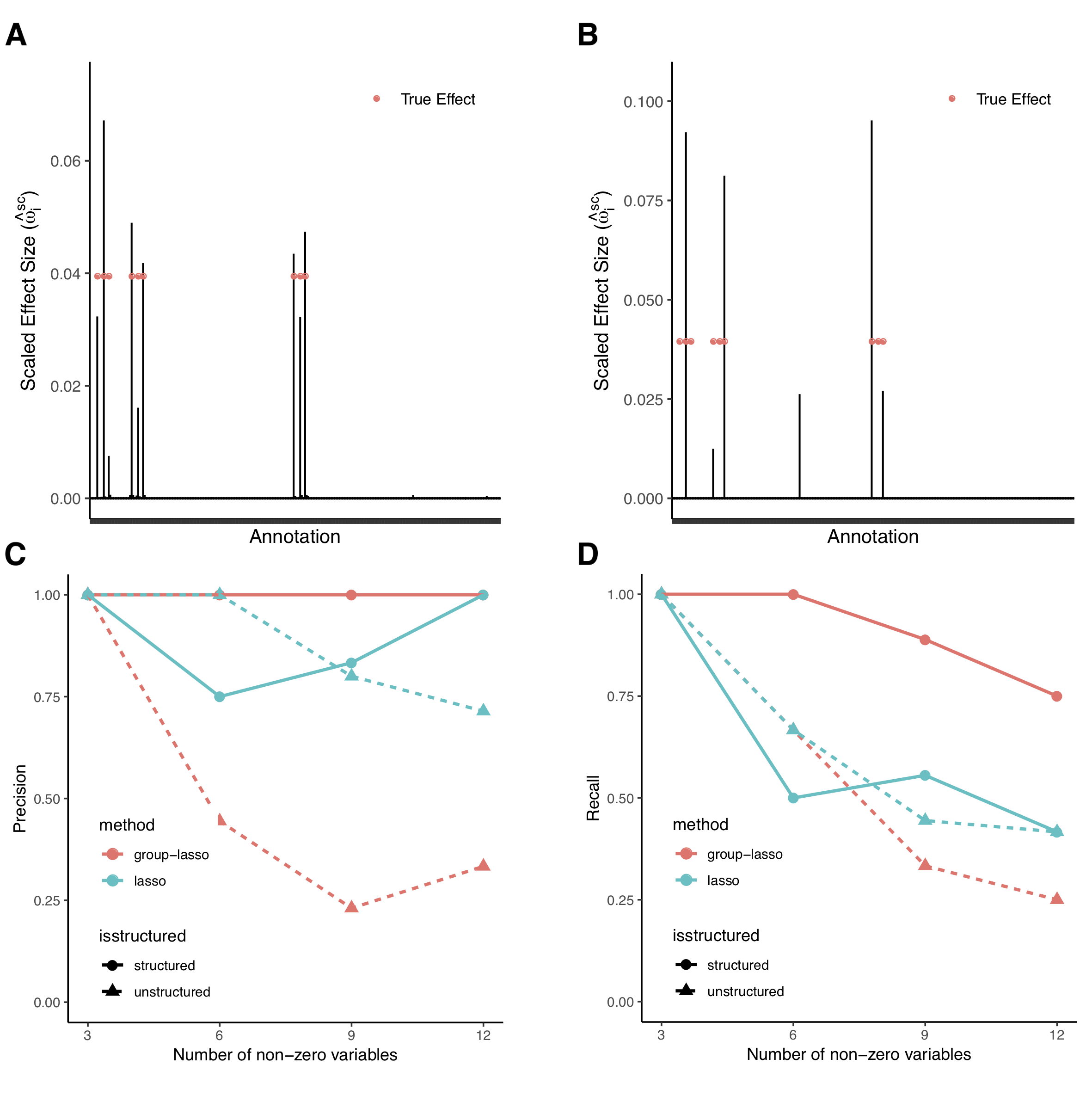

Supplement: S5 Fig — Shown are precision and recall for various simulation settings (see S1 Appendix) and two parameter settings. For each simulation setting we fitted BAGEA either making use of the meta-annotations available for cell type and assay type, (group-lasso) or ignoring the meta-annotation information and letting each ωi parameter be controlled individual υi parameter (lasso). Upper panels: shown are example results when fitting BAGEA either in group-lasso setting (A) or lasso setting (B), in the structured simulation setting with 9 variables (see S1 Appendix for simulation details). True effect sizes for ω are indicated via red dots. Scaled BAGEA estimates of ω are given as black lines (We scaled ω to account for differences in estimates of ν^ versus ν. We multiplied each element of ν by the coverage of its associated annotations and summed the resulting vector. We treated the estimate ν analogously and divided the two to get the scaling factor for ω. These scaling factors where 0.83 and 0.90 for the group-lasso (A) and lasso (B) setting respectively). When defining all scaled effect size estimates above 0.01 as positives and below 0.01 as negatives, we see that both settings yield a precision of one, whereas group-lasso also had a precision of 1.0 and recall of 0.88 and lasso had a precision of 0.83 and recall of 0.55 (five out of nine annotations recovered, one false positive). When looking at precision (C) and recall (D) across all simulation settings, we see that precision and recall drop as more variable are added. As expected, in an unstructured simulation setting, it is disadvantageous to enforce a structure on the estimates via the group-lasso setting. On the other hand, group-lasso maintains good precision and recall in a structured setting with up to 12 variables. (TIFF) [file pcbi.1007770.s006.tiff]

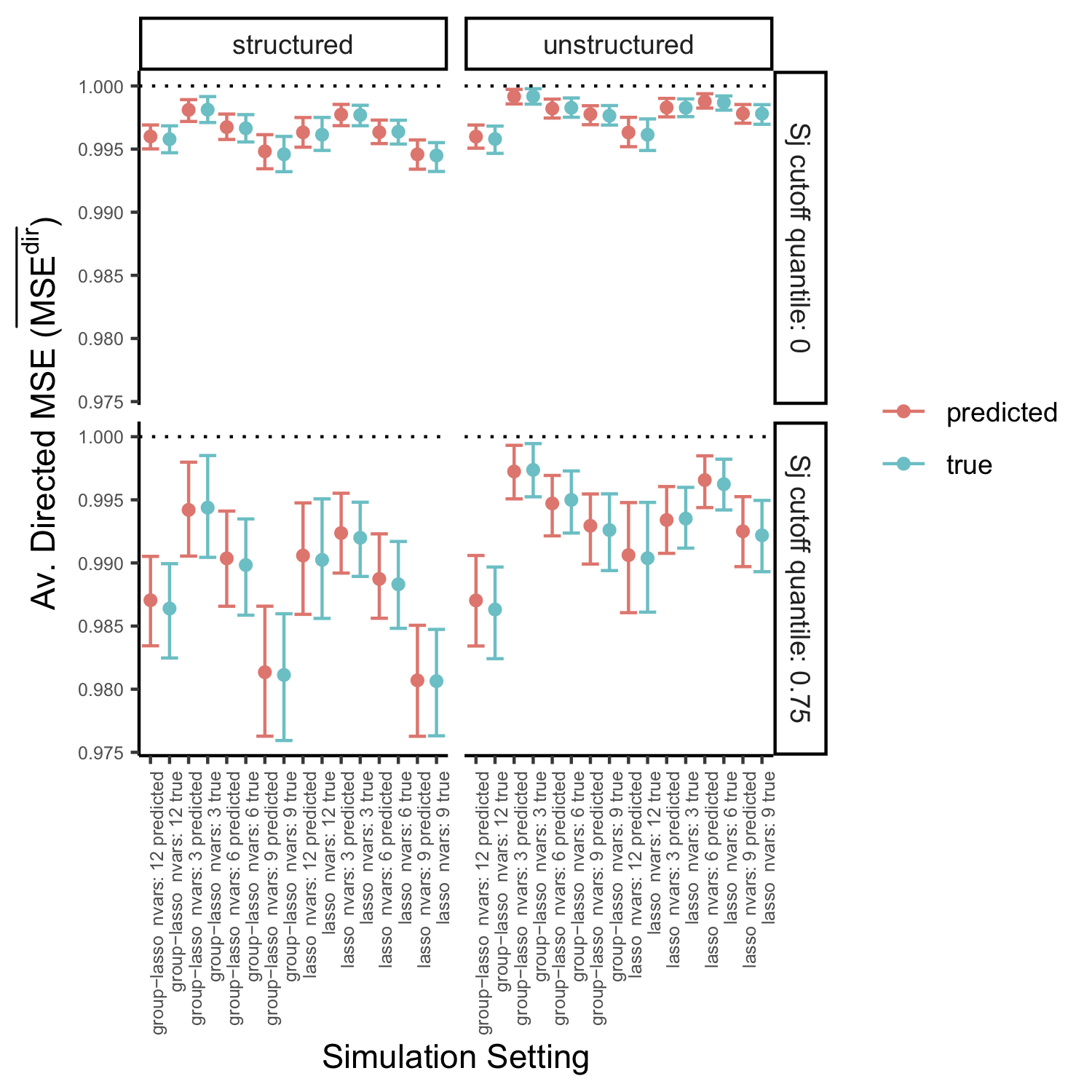

Supplement: S6 Fig — Shown are average MSEjdir values for all genes in the test set (chromosomes 3). The Upper panel shows average MSEjdir across all test genes, whereas the lower panel shows average MSEjdir for genes in the top quartile in terms of Sj. We see that the performance is very close to optimal even for settings where BAGEA did not select the correct variables, suggesting that the selected variables were highly correlated to the correct variables. (TIFF) [file pcbi.1007770.s007.tiff]

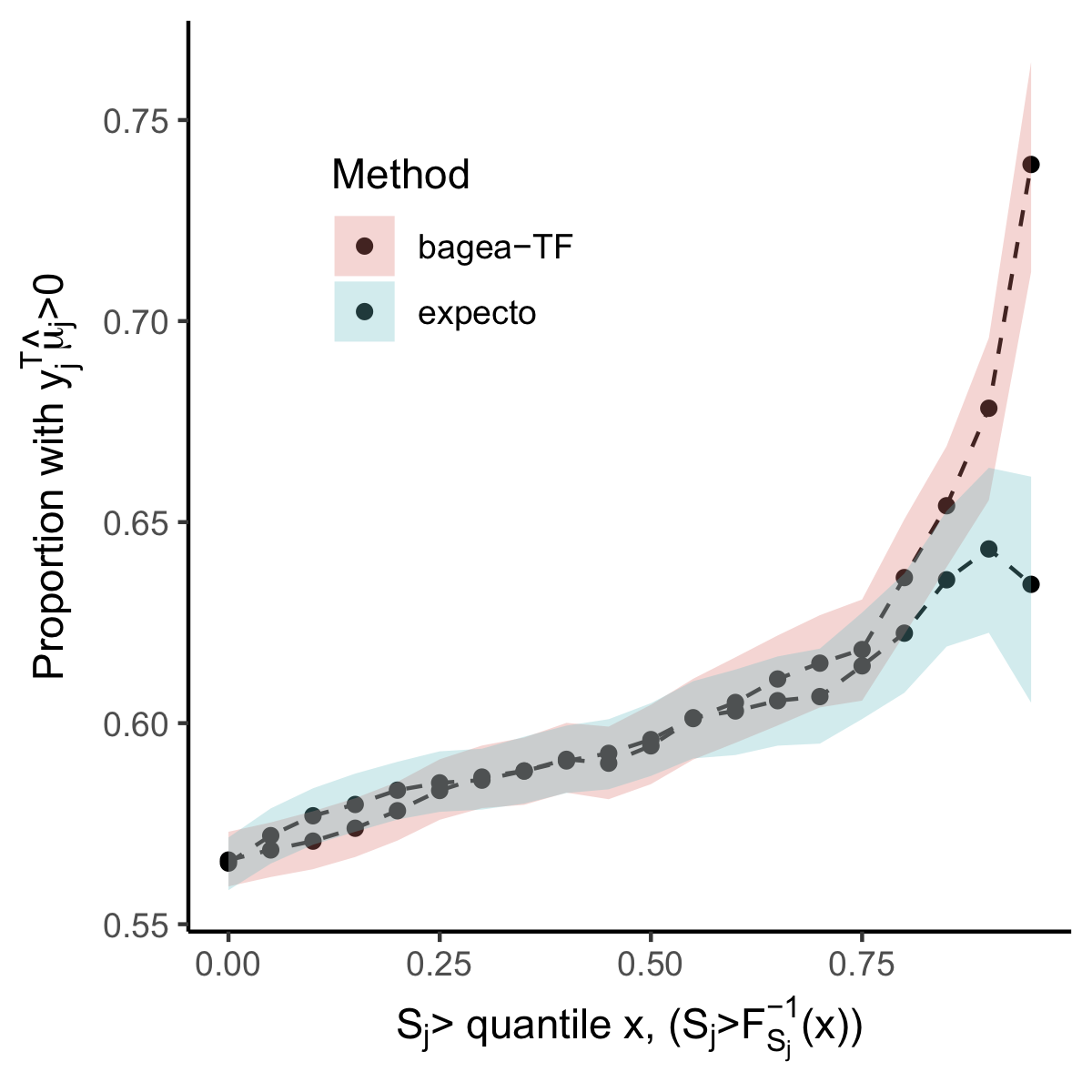

Supplement: S7 Fig — Shown is the comparison between BAGEA and ExPecto for 13 the GTEx experiments w.r.t. agreement between gene expression and the estimated directed predictor. (Sj > quantile x ((Sj>FSj-1(x))): Per GTEx experiments, genes were sorted by the squared magnitude Sj (Sj were computed for both BAGEA and ExPecto separately, i.e. for ExPecto SjExP was used). For each GTEx experiment, the top n-th percent of genes w.r.t Sj were then used to calculate the proportion of genes with positive yjTμ^j. (Proportion with yjTμ^j): The proportion of genes for which the scalar product between the gene expression vector yj and the directed predictor μ^j was larger than 0. We see that for genes with large relative effect sizes, BAGEA leads to higher concordance between yj and μ^j. 95% confidence band is derived by bootstrap sampling. (TIFF) [file pcbi.1007770.s008.tiff]

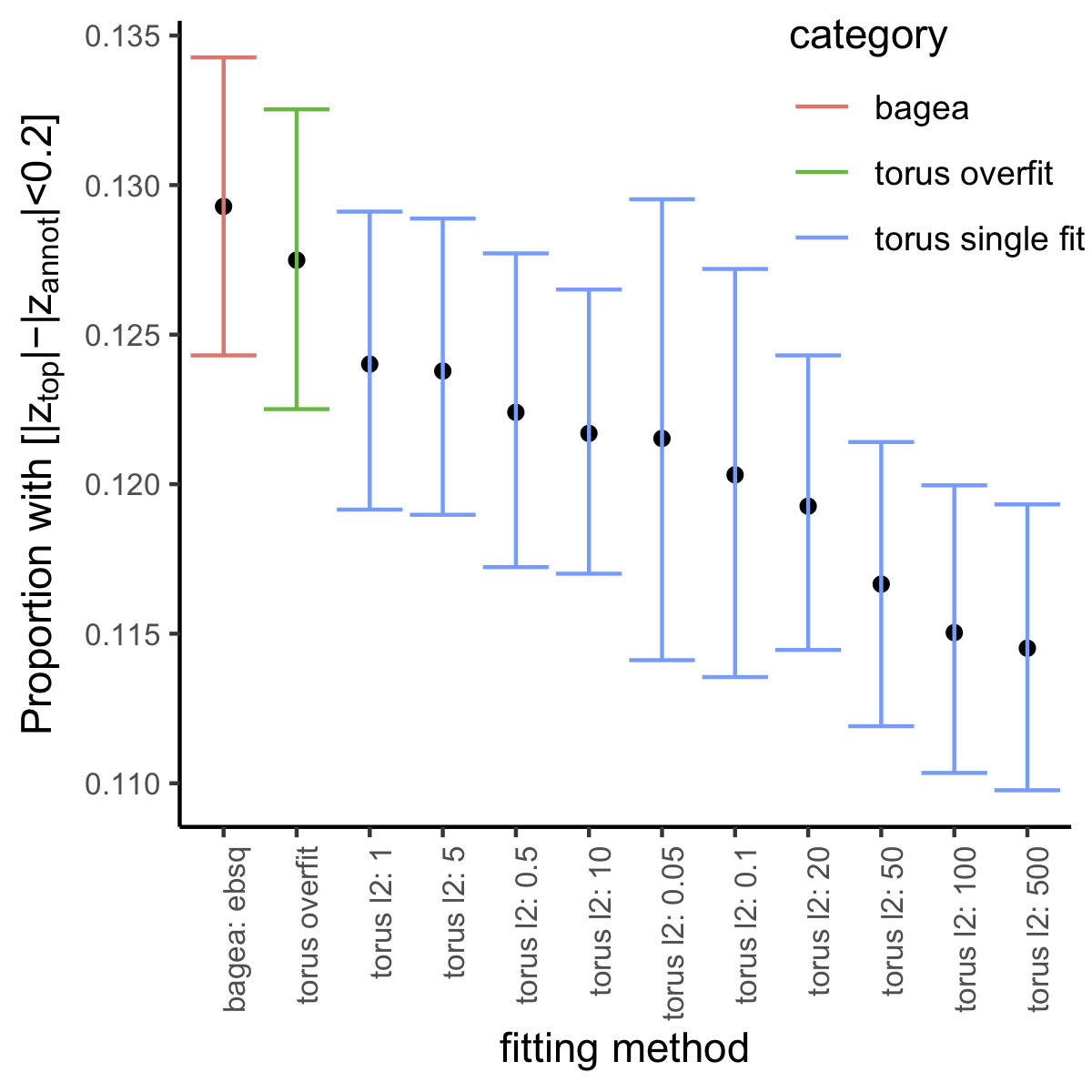

Supplement: S8 Fig — Shown is the comparison between BAGEA and Torus for 13 the GTEx experiments. To evaluate a method, we determined for each gene in the test set the SNP with the highest prior of being causal. For Torus, this amounted to ranking SNPs based on the scalar product between the SNP’s annotations and their estimated effect sizes. For BAGEA, we ranked SNPs based on E[bij2|G], where G refers to all global BAGEA parameter estimates (see S1 Appendix). (fitting method): The various methods used in the fitting and evaluation. For Torus we used various l2 parameter settings as well as an overfitting strategy as upper bound (see S1 Appendix for details) [14, 15]. (Proportion with |ztop| − |zannot| < 0.2): To evaluate a given method, we picked the SNP for each gene in the test set for which the method predicted the largest absolute effect sizes based on the annotations alone and recorded its z-score (denoted |zannot|). We then compared this value to the overall largest absolute z-score for this gene (denoted |ztop|). We evaluated the power by the proportion of genes for which |ztop| − |zannot| was below 0.2. 95% confidence interval is derived by bootstrap sampling. (TIFF) [file pcbi.1007770.s009.tiff]

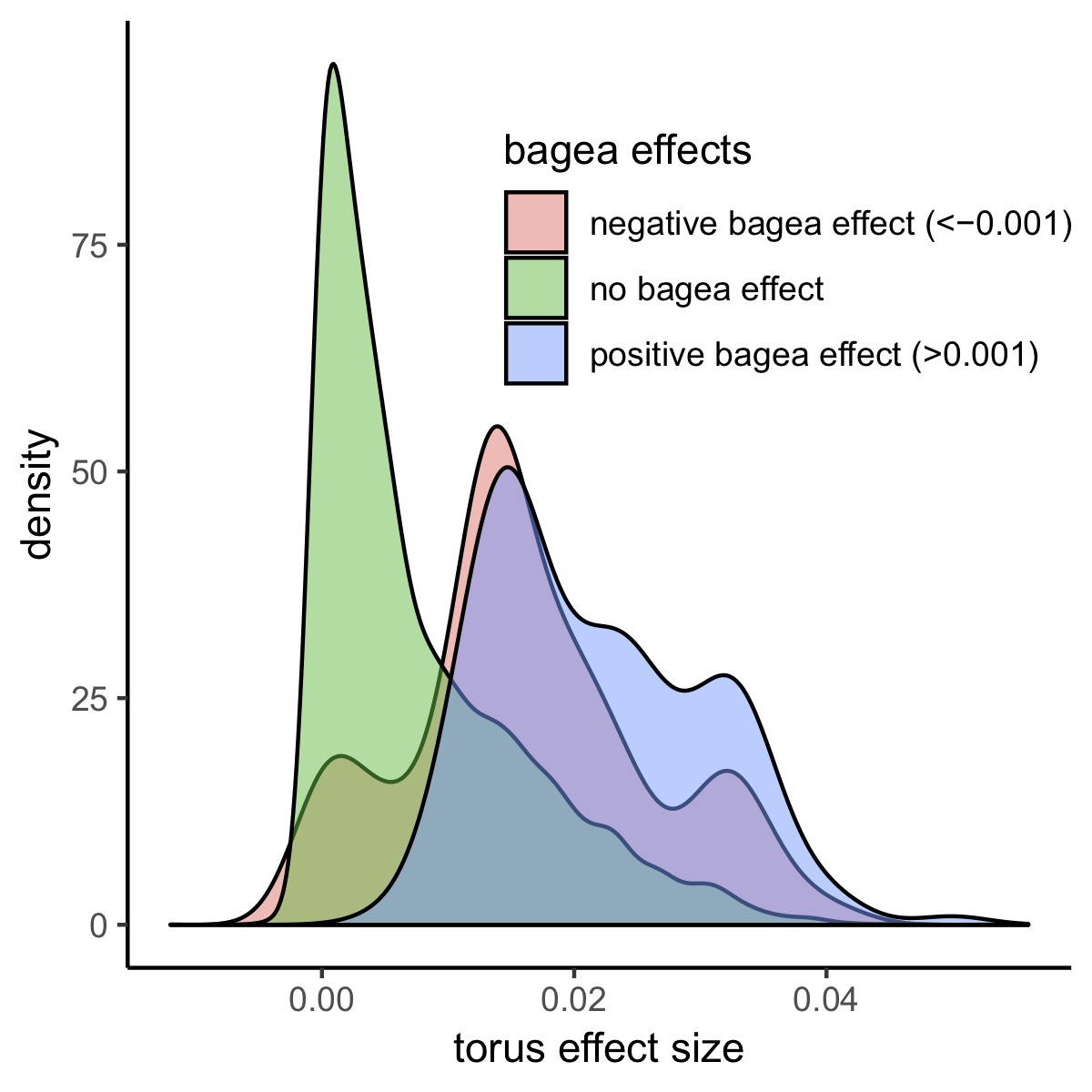

Supplement: S9 Fig — Shown are the distribution of effect size estimates of Torus when fitted on 13 GTEx datasets for the TF annotation subset (l2 = 100). As Torus was run in ridge mode, few effects were very close to zero. BAGEA in its default parameter resembles lasso, in that it only selects a limited number of effect sizes substantially different from zero. When fitting BAGEA using the same datasets in lasso mode, we saw 257 annotations overall larger than 0.001 (of which 192 where also larger than 0.01). When color-coding those 257 effect sizes based on direction and comparing them against the rest, we saw that the Torus effect sizes were markedly shifted away from zero for both the positive and the negative effect size BAGEA group. (TIFF) [file pcbi.1007770.s010.tiff]

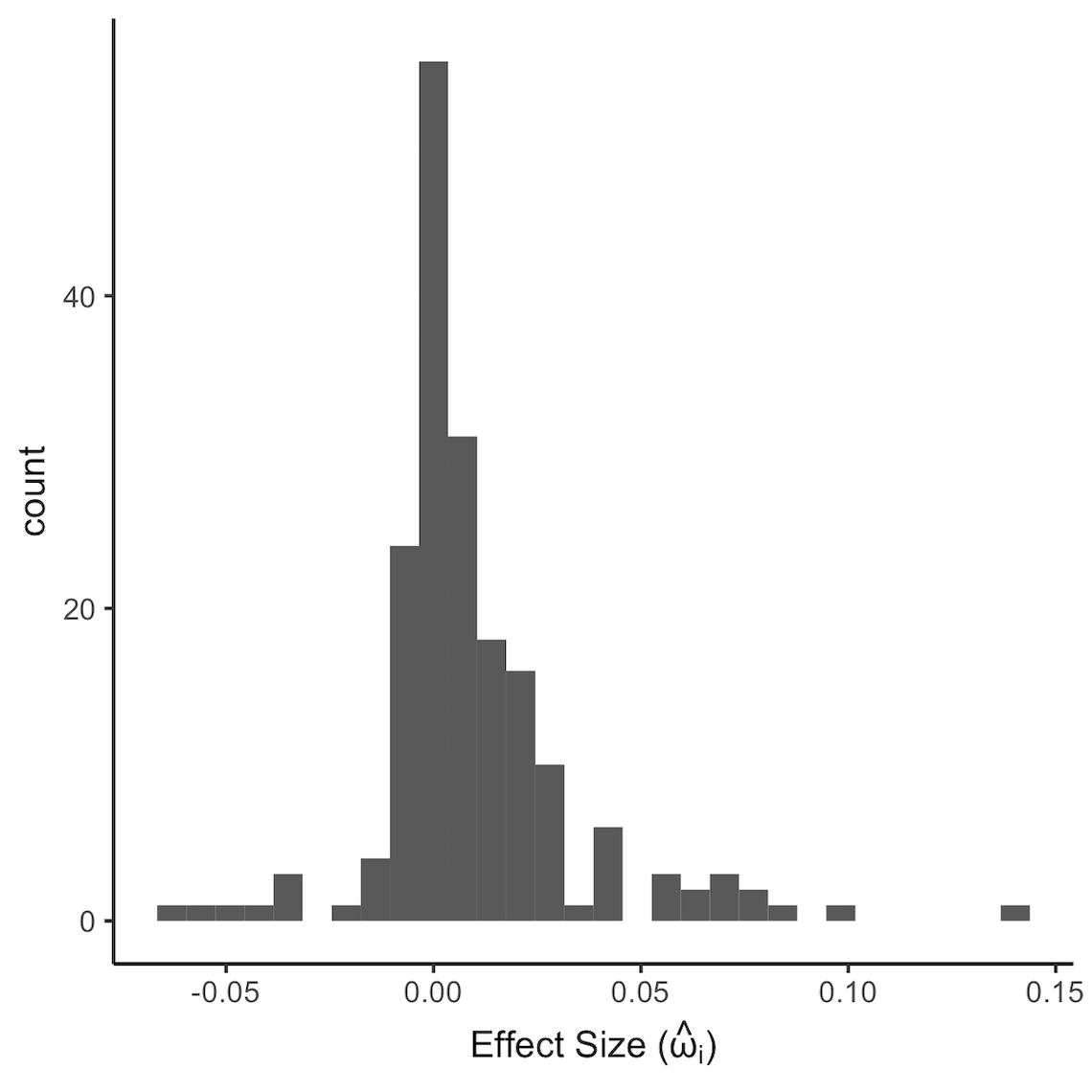

Supplement: S10 Fig — Displayed are estimated directed annotation effect sizes ω^ for all GTEx (and GEAUVADIS) datasets, with values with absolute value below 10−3 removed. Shown are results when fitting on data from all autosomes. (TIFF) [file pcbi.1007770.s011.tiff]

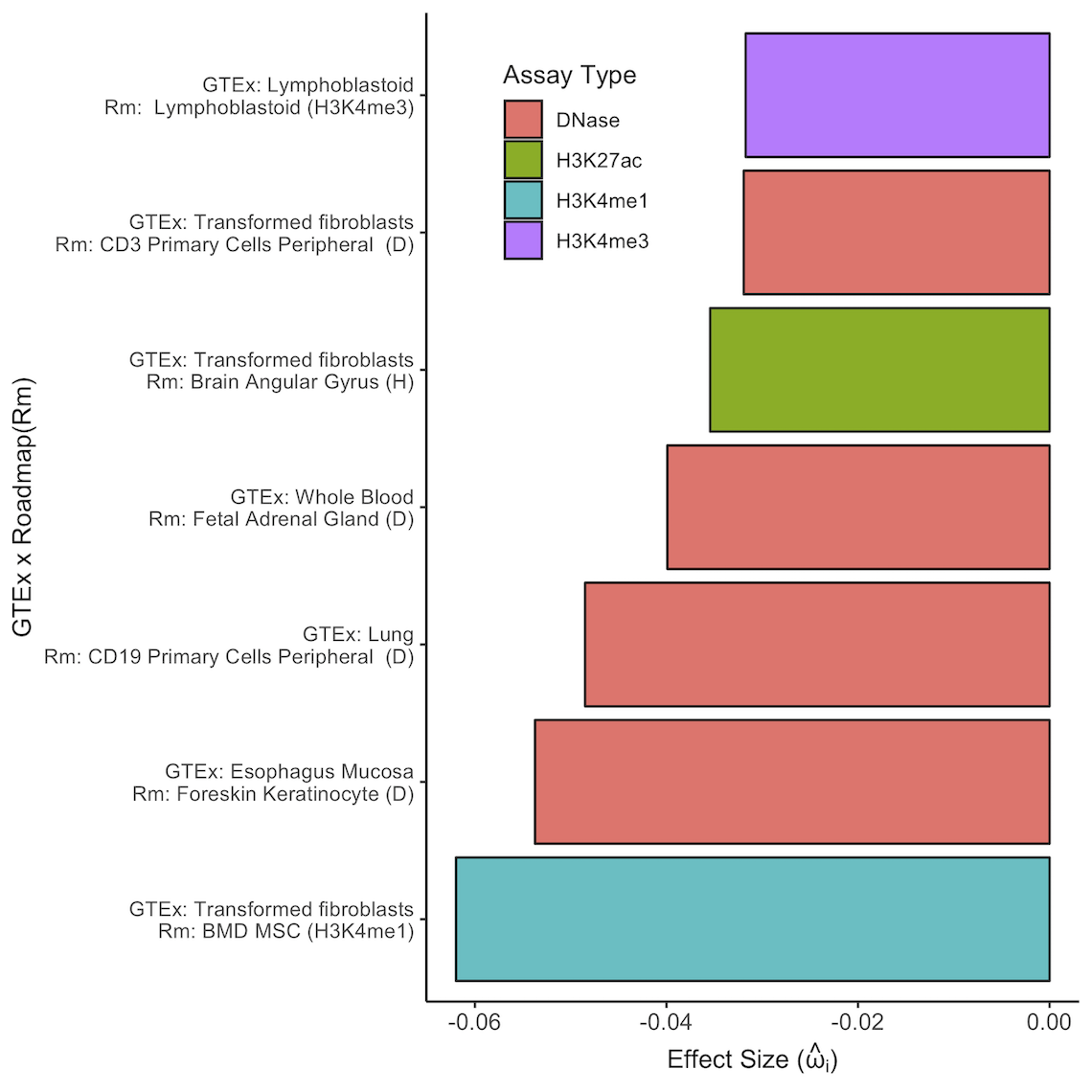

Supplement: S11 Fig — Shown are the largest negative directed annotation effect from fitting 14 different GTEx (and GEAUVADIS) eQTL summary statistics datasets using Histone and DHS ExPecto predictions derived from Roadmap. (TIFF) [file pcbi.1007770.s012.tiff]

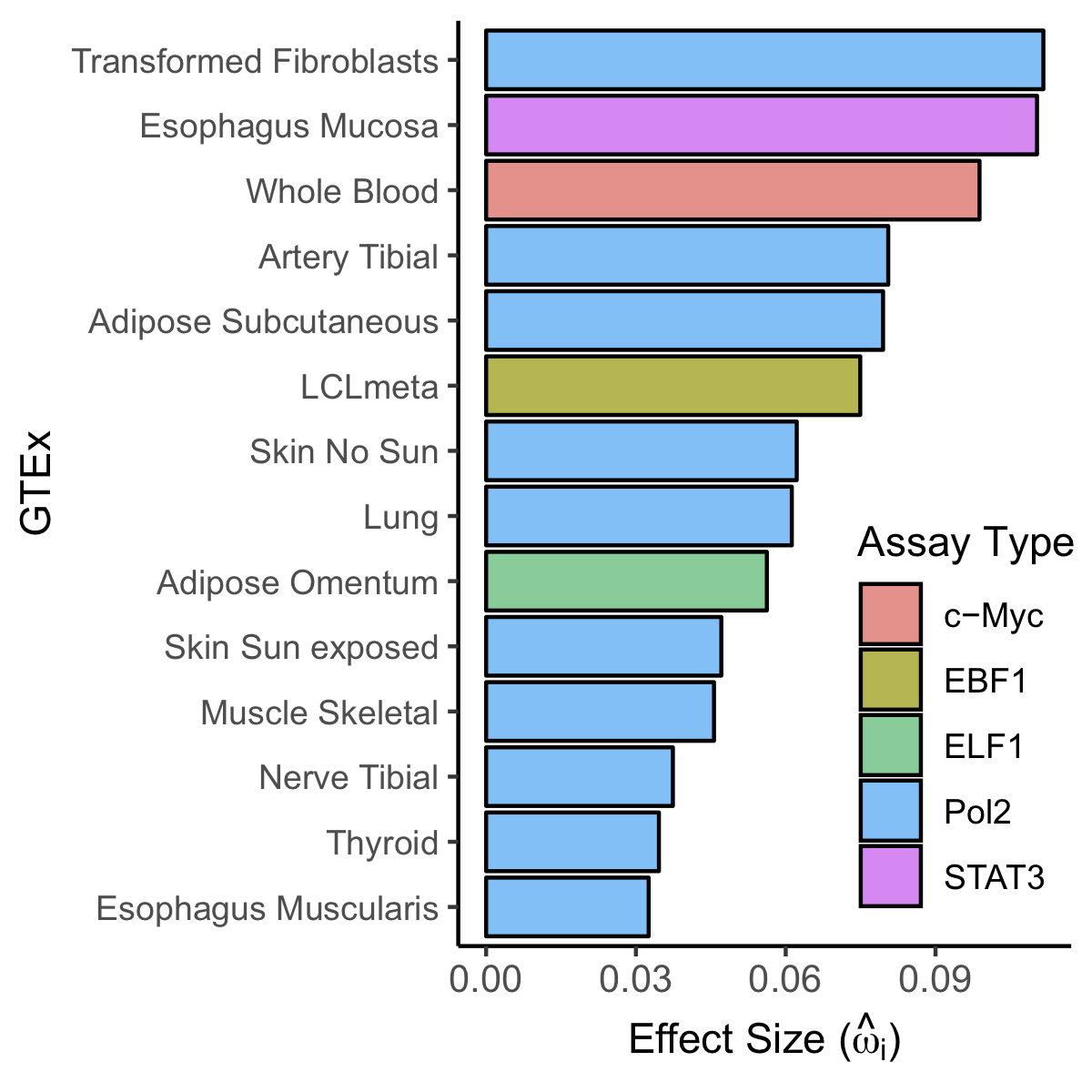

Supplement: S12 Fig — Shown is the largest directed annotation effect for each of the fitted 14 different GTEx (and GEAUVADIS) eQTL summary statistics datasets using ExPecto predictions derived from ENCODE non-histone ChIP-seq experiments [33]. (TIFF) [file pcbi.1007770.s013.tiff]

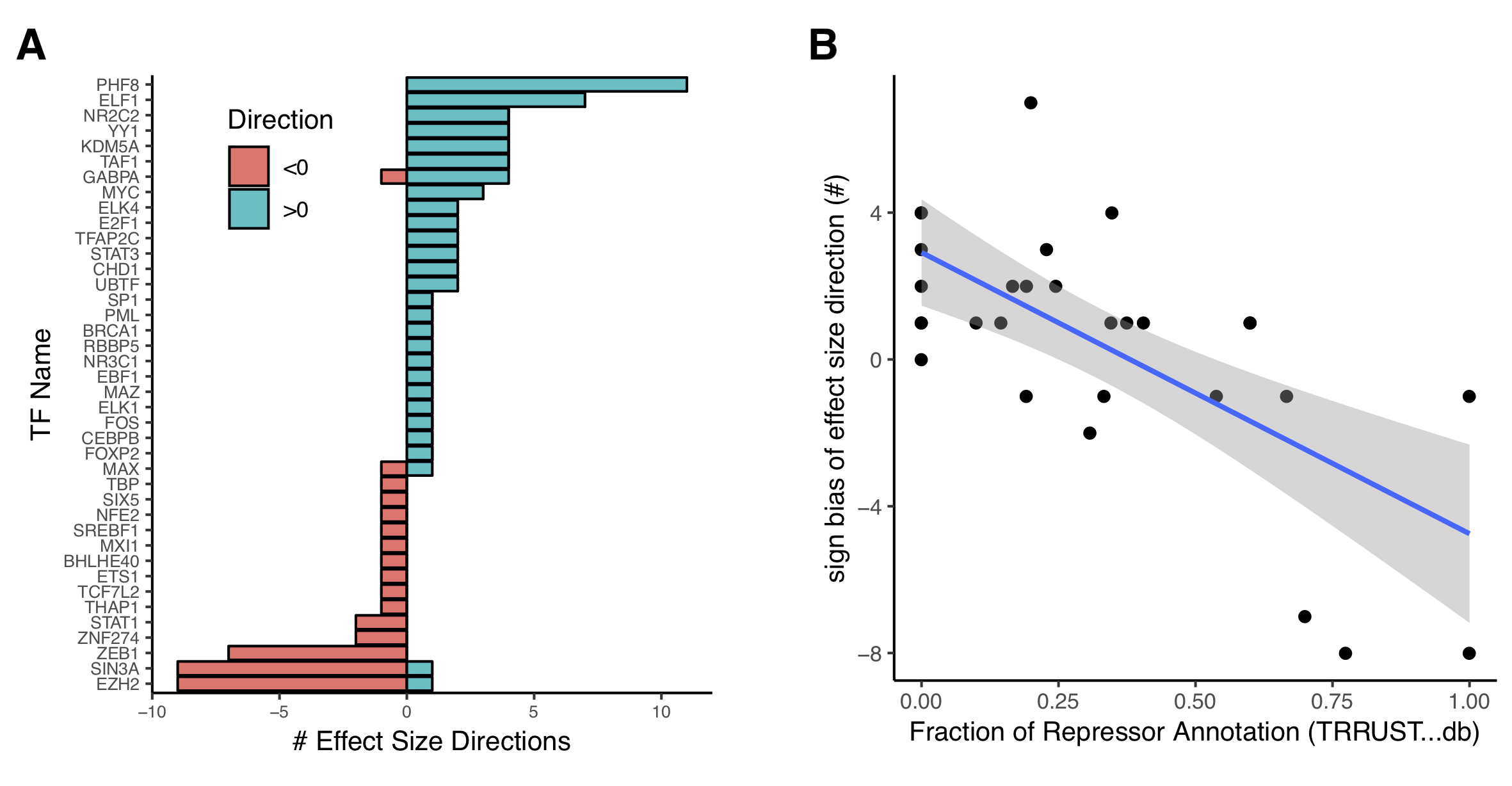

Supplement: S13 Fig — After removal of Pol2 from the TF annotations subset, we fit BAGEA to the GTEx summary statistics using lasso mode. (A) Shown are the number of GTEx experiments for which a given TF-ChIP-Seq assay shows a postively or negatively signed effect with absolute value above 0.01 (If multiple annotations mapped to the same TF we summed the effects, this step only affected a few TFs because of the regularization strategy employed). (B) Shown is the comparison between BAGEA’s prediction of repressor/activator activity of a TF’s with predictions derived from the trrust-db v2 [46]. (sign bias of effect size direction (#)): For each TF we take the difference between the number of postive effect directions (blue bar in panel (A)) and the number of negative effect directions (red bar in panel (A)) to get a prediction of whether a TF acts as activator (>0) or repressor (<0). (Fraction of Repressor Annotation (TRRUST-db)): The fraction of annotations in the human TTRUST db (human) for a given TF which claimed repressor activity among all annotations with a clear assigned direction (i.e. after removal of all annotations with unknown direction from TRRUST-db). We see a clear dependence between results from TRRUST (unidirectional p-value lower than 0.0001, R2 = 0.43), suggesting that results from BAGEA are in broad agreement with the literature in terms of determining activator and repressor TFs. (TIFF) [file pcbi.1007770.s014.tiff]

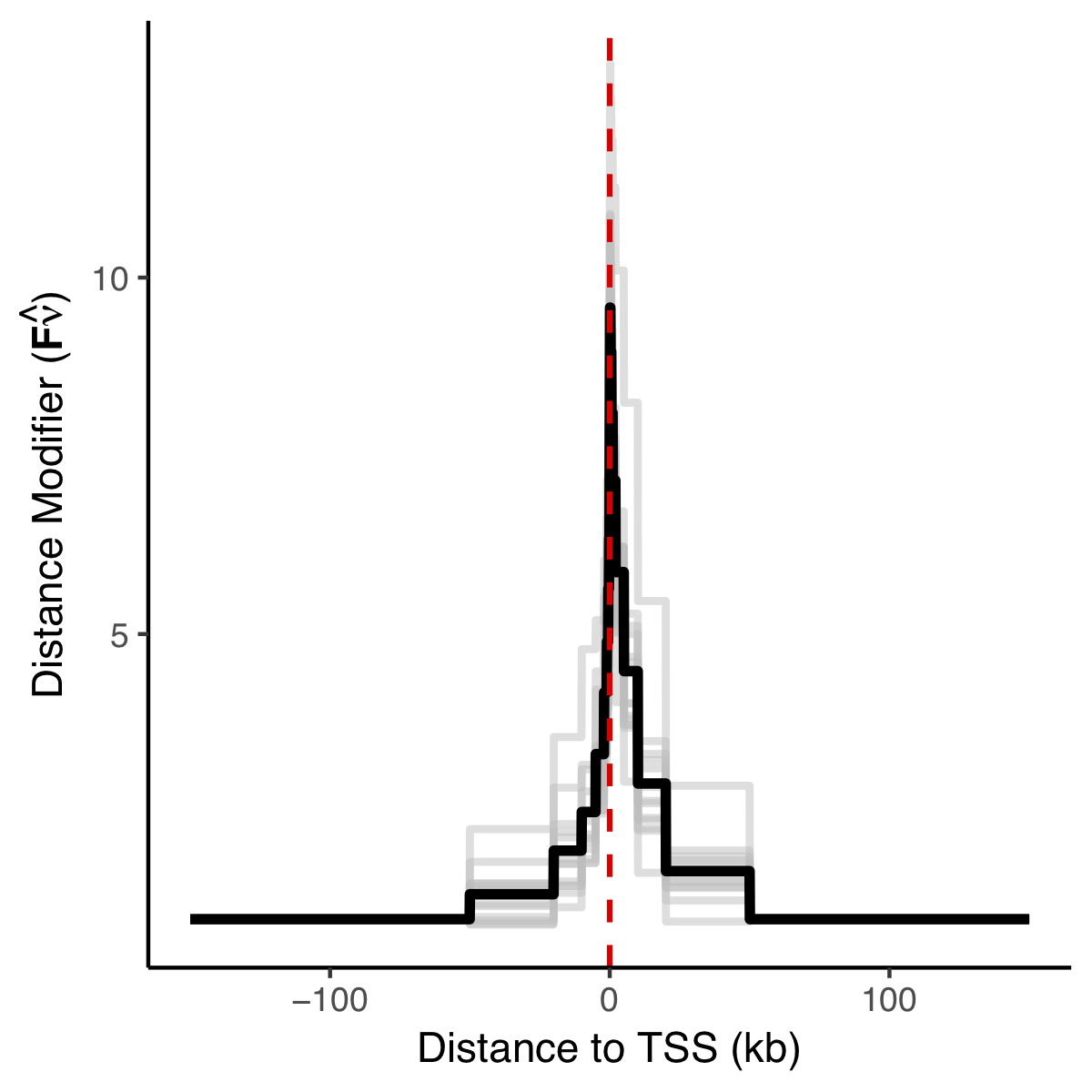

Supplement: S14 Fig — Shown is the estimated distance modifier of the directed component, Fν^ for all GTEx experiments when fit with the TF annotation subset in lasso mode. Individual results are plotted in grey and averages are plotted in black. While there is some fluctuation for individual results around the mean, the general peak shape is respected in all cases. (TIFF) [file pcbi.1007770.s015.tiff]

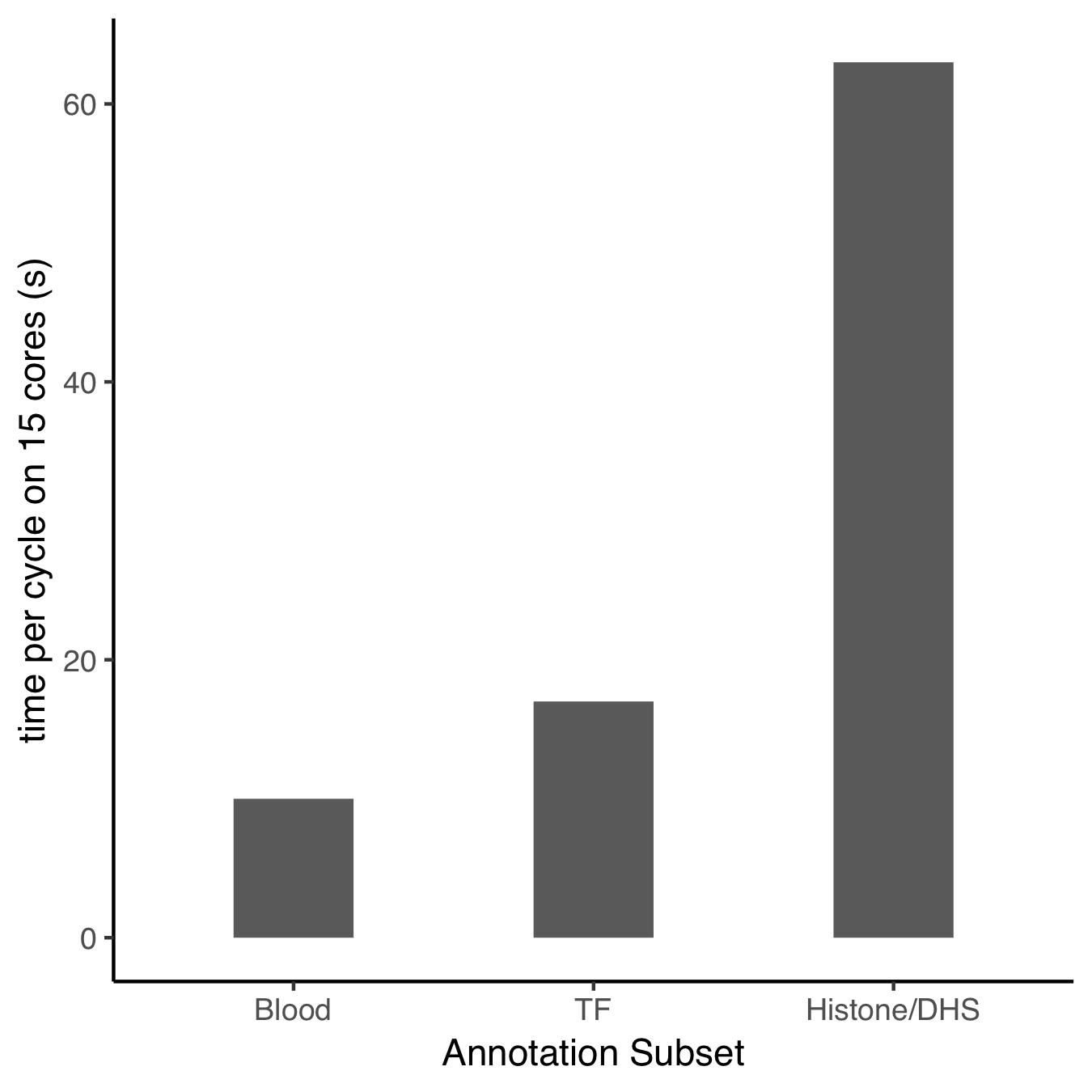

Supplement: S15 Fig — Shown is the speed with wich each updating iteration of the variational algorithm takes for the main analyses performed. For Blood we used the setting of the monocyte analysis (see for instance Fig 2). For TF and Histone/DHS we used the settings used in the respective GTEx analyses (see for instance Fig 4). All analyses were performed on an AWS r4 × 4 instance using 15 cores. As we ran the algorithm for 300 iterations, we see that in this setting, the algorithm took between 50 minutes and 5 and a half hours to complete. (TIFF) [file pcbi.1007770.s016.tiff]
