# Supplementary material for: A framework for integrating directed and undirected annotations to build explanatory models of cis-eQTL data
Source: PLoS Comput Biol. 2020 Jun 9;16(6):e1007770. doi: 10.1371/journal.pcbi.1007770 (PMC7332077; doi:10.1371/journal.pcbi.1007770)
Supplement: Attachment — Submitted filename: PCOMPBIOL-D-19-01078_annotated.pdf [file pcbi.1007770.s022.pdf]

PLOS Computational Biology

A framework for integrating directed and undirected annotations to build explanatory models of cis-eQTL data

--Manuscript Draft--

**Manuscript Number:**	PCOMPBIOL-D-19-01078
**Full Title:**	A framework for integrating directed and undirected annotations to build explanatory models of cis-eQTL data
**Short Title:**	Integrating directed and undirected annotations to build explanatory models of cis-eQTLs
**Article Type:**	Research Article
**Keywords:**	cis-eQTL analysis; Data integration
**Abstract:**	A longstanding goal of regulatory genetics is to understand how variants in genome sequences lead to changes in gene expression. Here we present a method named Bayesian Annotation Guided eQTL Analysis (BAGEA), a variational Bayes framework to model cis-eQTLs using directed and undirected genomic annotations. We used BAGEA to integrate directed genomic annotations with eQTL summary statistics from tissues of various origins. This analysis revealed epigenetic marks that are relevant for gene expression in different tissues and cell types. We estimated the predictive power of the models that were fitted based on directed genomic annotations. This analysis showed that, depending on the underlying eQTL data used, the directed genomic annotations could predict up to 1.5\% of the variance observed in the expression of genes with top nominal eQTL association p-values $<10^{-7}$. For genes with estimated effect sizes in the top 25\% quantile, up to 5\% of the expression variance could be predicted. Based on our results, we recommend the use of BAGEA for the analysis of cis-eQTL data to reveal annotations relevant to expression biology.
**Additional Information:**	
**Question**	**Response**
**Financial Disclosure**<br><br>Enter a financial disclosure statement that describes the sources of funding for the work included in this submission. Review the submission guidelines for detailed requirements. View published research articles from *PLOS Computational Biology* for specific examples.<br><br>This statement is required for submission and **will appear in the published article** if the submission is accepted. Please make sure it is accurate.	This research was supported by Verge Genomics, a venture funded drug discovery company (https://www.vergegenomics.com/).

**Unfunded studies**<br>Enter: *The author(s) received no specific funding for this work.*<br><br>**Funded studies**<br>Enter a statement with the following details:<br>• Initials of the authors who received each award<br>• Grant numbers awarded to each author<br>• The full name of each funder<br>• URL of each funder website<br>• Did the sponsors or funders play any role in the study design, data collection and analysis, decision to publish, or preparation of the manuscript?<br>• **NO** - Include this sentence at the end of your statement: *The funders had no role in study design, data collection and analysis, decision to publish, or preparation of the manuscript.*<br>• **YES** - Specify the role(s) played.<br><br>* typeset	
**Competing Interests**<br><br>Use the instructions below to enter a competing interest statement for this submission. On behalf of all authors, disclose any competing interests that could be perceived to bias this work—acknowledging all financial support and any other relevant financial or non-financial competing interests.<br><br>This statement **will appear in the published article** if the submission is accepted. Please make sure it is accurate. View published research articles from *PLOS Computational Biology* for specific examples.	The authors have declared that no competing interests exist.

**NO authors have competing interests**<br><br>Enter: *The authors have declared that no competing interests exist.*<br><br>**Authors with competing interests**<br><br>Enter competing interest details beginning with this statement:<br><br>*I have read the journal's policy and the authors of this manuscript have the following competing interests: [insert competing interests here]*<br><br>* typeset	
**Data Availability**<br><br>Authors are required to make all data underlying the findings described fully available, without restriction, and from the time of publication. PLOS allows rare exceptions to address legal and ethical concerns. See the PLOS Data Policy and FAQ for detailed information.<br><br>A Data Availability Statement describing where the data can be found is required at submission. Your answers to this question constitute the Data Availability Statement and **will be published in the article**, if accepted.<br><br>**Important:** Stating 'data available on request from the author' is not sufficient. If your data are only available upon request, select 'No' for the first question and explain your exceptional situation in the text box.<br><br>Do the authors confirm that all data underlying the findings described in their manuscript are fully available without restriction?	Yes - all data are fully available without restriction
**Describe where the data may be found in full sentences. If you are copying our sample text, replace any instances of XXX with the appropriate details.**	Software package is available on github (https://github.com/dlampart/bagea/)

• If the data are **held or will be held in a public repository**, include URLs, accession numbers or DOIs. If this information will only be available after acceptance, indicate this by ticking the box below. For example: *All XXX files are available from the XXX database (accession number(s) XXX, XXX.).*<br>• If the data are all contained **within the manuscript and/or Supporting Information files**, enter the following: *All relevant data are within the manuscript and its Supporting Information files.*<br>• If neither of these applies but you are able to provide **details of access elsewhere**, with or without limitations, please do so. For example:<br><br>*Data cannot be shared publicly because of [XXX]. Data are available from the XXX Institutional Data Access / Ethics Committee (contact via XXX) for researchers who meet the criteria for access to confidential data.*<br><br>*The data underlying the results presented in the study are available from (include the name of the third party and contact information or URL).*<br>• This text is appropriate if the data are owned by a third party and authors do not have permission to share the data.<br><br>* typeset	
Additional data availability information:	

Powered by Editorial Manager® and ProduXion Manager® from Aries Systems Corporation

Victor Hanson-Smith, Ph.D.
Head of Computational Biology
Verge Genomics
Two Tower Place, Suite 940
South San Francisco, CA 94080
e-mail: victor@vergegenomics
Tel: (916) 221-2222

Dear Editor,

I am submitting to *PLOS Computational Biology* our manuscript titled "A Framework for Integrating Directed and Undirected Annotations to Build Explanatory Models of cis-eQTL Data" By David Lamparter, Raj Bhatnagar, Katja Hebestreit, T. Grant Belgard, Alice Zhang, and myself, for your consideration (all email addresses below).

Brief Summary
Here we present Bayesian Annotation Guided eQTL Analysis (**BAGEA**), a variational Bayes framework to model cis-eQTLs using directed and undirected genomic annotations.

Background and Summary of Work
A longstanding challenge in the field of human genetics has been to predict the phenotypic consequences of genetic variants. Solving this problem would allow for a deeper mechanistic understanding of genomic architectures, and it would make it possible to probe for rare variants that have mild phenotypic consequences. Recently, progress has been made in building such models for epigenetic marks. An important question is how to build such models for more complex phenotypes.

Here we present a Bayesian framework to tackle this problem for expression genetics. Our method, BAGEA, integrates the results from expression quantitative trait locus (eQTL) studies with directed and undirected genomic annotations as well as interactions thereof. Our model identifies which annotations are predictive of causal variants. Importantly, because BAGEA integrates directed annotations, it goes beyond estimating enrichments of causal variants in genomic annotations; rather, BAGEA also predicts the direction of expression effect by building a predictive model of eQTL effects based on annotations. Overall, BAGEA is a significant methodological step towards predicting phenotypic consequences from sequence variants alone.

We used our method to integrate the results of eQTL studies with more than 1,000 directed annotations. We show that up to 1.5% of the total expression variance could be predicted in an out-of-sample fashion. Furthermore, the annotations selected by the method to build these predictive models were relevant to the interrogated eQTL studies as they identified the relevant cell and tissue types.

Suitability for *PLOS Computational Biology*
We think that addressing the twin challenges of 1) understanding the regulatory code of gene expression and 2) building models to predict changes of gene expression based on the genomic sequence alone is a timely endeavour of general interest to the readers of *PLOS Computational Biology.* Further, we think our results are especially interesting to the community of researchers studying genetics of molecular phenotypes. BAGEA's methodology can be widely applied to build

predictive and explanatory models in many different molecular systems of interest. For this purpose we provide a publicly available implementation.

Thank you for your attention to our manuscript. We look forward to hearing from you.

Sincerely,

Victor Hanson-Smith

Victor Hanson-Smith, Ph.D

Author Email Addresses

David Lamparter: lamparterd@gmail.com

Rajat Bhatnagar: raj@vergegenomics.com

Katja Hebestreit: katja@vergegenomics.com

T. Grant Belgard: grant@bioinformaticscro.com

Alice Zhang: alice@vergegenomics.com

Victor Hanson-Smith: victor@vergegenomics.com

Manuscript
Click here to download Manuscript plos_main.pdf

A framework for integrating directed and undirected annotations to build explanatory models of cis-eQTL data

David Lamparter[1,¤a], Rajat Bhatnagar[1], Katja Hebestreit[1], T. Grant Belgard[1,¤b], Alice Zhang[1], Victor Hanson-Smith[1*],

1 Verge Genomics, South San Francisco, CA 94080, USA

¤a Current Address: Health 2030 Genome Center, 1202 Geneva, Switzerland
¤b Current Address: The Bioinformatics CRO, Niceville, FL 32578, USA
* victor@vergegenomics.com

Abstract

A longstanding goal of regulatory genetics is to understand how variants in genome sequences lead to changes in gene expression. Here we present a method named Bayesian Annotation Guided eQTL Analysis (BAGEA), a variational Bayes framework to model cis-eQTLs using directed and undirected genomic annotations. We used BAGEA to integrate directed genomic annotations with eQTL summary statistics from tissues of various origins. This analysis revealed epigenetic marks that are relevant for gene expression in different tissues and cell types. We estimated the predictive power of the models that were fitted based on directed genomic annotations. This analysis showed that, depending on the underlying eQTL data used, the directed genomic annotations could predict up to 1.5% of the variance observed in the expression of genes with top nominal eQTL association p-values $< 10^{-7}$. For genes with estimated effect sizes in the top 25% quantile, up to 5% of the expression variance could be predicted. Based on our results, we recommend the use of BAGEA for the analysis of cis-eQTL data to reveal annotations relevant to expression biology.

Author summary

Many geneticists wish to map changes in DNA sequences to changes in human traits and to understand these processes mechanistically. Here we present BAGEA, a framework to study this question for gene expression. Specifically, BAGEA models a genome variant's impact on gene expression based on established genome annotations. BAGEA predicts not only whether a variant has an impact on gene expression, but also the sign of the effect. We applied BAGEA to data sets from different tissues and cell types and found that annotations most predictive of gene expression in a given tissue were typically derived from similar tissues. Based on our results, we recommend the use of BAGEA to reveal annotations relevant to expression biology and to build predictive models of gene expression.

Introduction

A longstanding goal in the field of genetics is to accurately predict the phenotypic consequences of any given variant from the genome sequence alone, i.e. to 'read the genome' [1]. This would help to reveal the phenotypic effects of very rare variants even

if their effect is weak. The effects of such variants are typically studied via whole genome sequencing studies. However these studies often have limited statistical power because, by definition, there are few carriers in any sampled population [2].

Recently, progress has been made in predicting epigenetic marks and transcription factor (TF) binding from genome sequence alone; these sequence-based models predict the effect of any given sequence variant on epigenetic marks (and TF binding) [3–7]. The question now is how to extend these models to predict effects on genetically complex phenotypes, such as common diseases. A mechanistic stepping stone between the regulation of epigenetic marks and the regulation of complex phenotypes is the regulation of gene expression, as suggested by the previous observation that disease-causing sequence variants are enriched in gene expression quantitative trait loci (eQTLs) [8, 9]. Thus, there is a need for sequence-based models to predict gene expression.

One strategy to build sequence-based models of gene expression is to leverage sequence-based models of epigenetic marks. Results of these sequence-based models can be interpreted as *directed genome annotations*. A genome annotation is defined as a collection of genome regions that have a shared property such as coverage by a particular epigenetic mark, or evolutionary conservation across species. Each region can potentially carry an intensity value to denote the annotation strength, such as the strength of conservation. For *directed annotations*, the sign of its intensity value depends on characteristics of the sequence in the region, such as the presence of a specific allele. A simple motivating example is that of a SNP in a TF binding site. In this situation, the TF can have higher binding affinity for one allele versus the other allele. This can cause consistent directional transcriptional effects: the allele inhibiting binding of an activating TF for instance should lead to decreased expression of the target gene. One strategy to express this effect as a directional annotation would be to use TF position weight matrices that calculate TF affinity for a given sequence, while computationally more sophisticated methods express the same relationship using deep neural networks [3–7].

Methods to evaluate the effect of directed genome annotations on gene expression have recently been proposed [7, 10]. Specifically, *Zhou et al.* predicted variant impact without exploiting eQTL data using models that predict expression from chromatin patterns directly [7]. *Reshef et al.* presented a fast method to determine which directed annotations are enriched in variants causal for a given phenotype. However, the method from *Reshef et al.* is geared towards screening and hypothesis testing rather than towards detailed predictive modeling. For instance, the Reshef model does not account for interactions between the effect of an annotation and the distance to the transcription start site (*TSS*).

Here we present a new predictive model of gene expression, named Bayesian Annotation Guided eQTL Analysis (*BAGEA*). *BAGEA* is a variational Bayes modeling framework to analyze eQTLs using both directed and undirected annotations. *BAGEA* can model interactions between these annotations by weighting the impact of the directed annotation based on the undirected annotations. Consequently, *BAGEA* can directly model phenomena relevant to genetic architecture, such as the relatively larger impact of SNPs close to the TSS on directed annotations compared to that of distal SNPS, making BAGEA mores useful for predictive modeling. *BAGEA*'s results are interpretable and highlight genome annotations that are particularly predictive for gene expression. Further, *BAGEA* can model multiple causal SNPs per region. Our software implementation of *BAGEA* can be run on summary statistics using external linkage disequilibrium (LD) information as well as on individual level genotype data directly. Optionally, using a low rank approximation of the LD information improves run-time and decreases *BAGEA*'s memory requirements.

We used *BAGEA* to analyze results from a *cis*-eQTL meta-analysis in human monocytes and from *cis*-eQTL summary statistics derived from tissues of various origins [9, 11]. As additional input, we gave the method regulatory impact predictions of common variants on epigenetic marks from a recent deep neural network model [7]. We specified these predictions as directed directional annotations in the method. We show that *BAGEA* highlighted biologically sensible annotations as particularly predictive of eQTLs. Further we estimated the predictive power of the directed annotations for various eQTL data sets. Overall, our results suggest that *BAGEA* is a useful framework to build predictive models of gene expression based on directed annotations, find biologically relevant annotations, and benchmark methods that produce such directed annotations.

Model Overview

BAGEA models gene expression as dependent on SNP genotypes in *cis*. In general, SNP effects on gene expression depend on both directed and undirected annotations (Fig 1A). *BAGEA* builds predictors of gene expression and ranks annotations by their impact on gene expression. For every gene j, *BAGEA* takes as input a genotype matrix $\boldsymbol{X}_j$, an expression vector $\boldsymbol{y_j}$, annotation matrices $\boldsymbol{V}_j$, $\boldsymbol{F}_j$ and $\boldsymbol{C}_j$. $\boldsymbol{X}_j$ has dimensions $(n \times m_j)$, where n is the number of individuals assayed, and m_j is the number of SNPs in *cis* of gene j's *TSS*. The matrices $\boldsymbol{V}_j$ $\boldsymbol{F}_j$ and $\boldsymbol{C}_j$ are of dimensions $(m_j \times s)$, $(m_j \times q)$, and $(m_j \times t)$ respectively, where s, q and t are the number of annotations used. *BAGEA* models gene expression as a linear combination of SNP genotypes:

$$\boldsymbol{y}_j = \boldsymbol{X}_j \boldsymbol{b}_j + \boldsymbol{\epsilon}_j, \tag{1}$$

where $\boldsymbol{\epsilon}_j$ is an $i.i.d$ normal noise vector and $\boldsymbol{b}_j$ is a vector of SNP effects. The vector $\boldsymbol{b}_j$ is modeled as:

$$\boldsymbol{b}_j \sim N_{m_j}((\boldsymbol{V^j \omega}) \cdot (\boldsymbol{F^j \nu})), diag(\boldsymbol{\alpha}_j)^{-1}), \tag{2}$$

where $\cdot$ is the pointwise product (i.e. Hadamard product) and the $diag(\boldsymbol{x})$ operator stands for a diagonal matrix with elements on the diagonal set to $\boldsymbol{x}$. Detailed descriptions of the terms are as follows:

- $\boldsymbol{V}^j$ encodes directed annotations. In our applications of *BAGEA*, $\boldsymbol{V}^j$ is previously computed from sequence-based models, where each column in $\boldsymbol{V}^j$ represents an epigenetic mark and each row represents a SNP. Each entry in $\boldsymbol{V}^j$ expresses the predicted effect of a genotype change on the epigenetic mark in question.

- $\boldsymbol{F}^j$ encodes undirected annotations. Each element in $\boldsymbol{F}^j$ expresses the presence or absence of the annotation at a SNP's location. In our applications of *BAGEA*, $\boldsymbol{F}^j$ is derived from the relative positions of a SNP and gene j's *TSS*, where each column represents a particular region around the *TSS*. For example, if a column in $\boldsymbol{F}^j$ encodes a region of 20 kilobases (KB) upstream from the *TSS*, all entries for rows corresponding to SNPs within 20 KB upstream of that *TSS* will be set to 1 and entries for all other rows will be set to 0.

- $\boldsymbol{\omega}$ and $\boldsymbol{\nu}$ are vectors that are estimated by *BAGEA*. Specifically, $\boldsymbol{\omega}$ and $\boldsymbol{\nu}$ are the effects of annotations in $\boldsymbol{F}^j$ and $\boldsymbol{V}^j$ on the SNP effects $\boldsymbol{b}_j$.

- $\boldsymbol{\alpha}_j^{-1}$ is a vector that is estimated by *BAGEA* and models the variances of elements of $\boldsymbol{b}_j$. Allowing different variances for the elements of $\boldsymbol{b}_j$ typically produces sparse estimates where most elements in $\boldsymbol{b}_j$ are close to zero [12]. Further, $\boldsymbol{\alpha}$ is modeled as dependent on the undirected annotation matrix $\boldsymbol{C}_j$. $\boldsymbol{C}_j$ can potentially be identical to $\boldsymbol{F}_j$ but can model different undirected annotations as well (see Method Details).

Fig 1. *Illustration of BAGEA model components.*
(A) The core components of the *BAGEA* model in the summary statistics formulation. Observed variables are in squares while estimated variables are circled. Given are $\boldsymbol{z}_j$, the eQTL z-scores for gene j, as well as the LD matrix $\boldsymbol{\Sigma}_j$, defining the correlation between summary statistics. Further, z-scores are influenced by the true eQTL effects $\boldsymbol{b}_j$. These effects in turn depend on directed and undirected annotations, $\boldsymbol{V}^j$ and $\boldsymbol{F}^j$ respectively. While undirected annotations can cover regions of any size, directed annotation have the same size as the genomic variants themselves. The impact of annotations on $\boldsymbol{b}_j$ is estimated from the data via $\boldsymbol{\omega}$ and $\boldsymbol{\nu}$. (B) An example of the modeling of different priors of elements of $\boldsymbol{\omega}$ using meta-annotations via $\boldsymbol{\upsilon}$ variable vectors. We assume that directed annotations are available for nine annotations, which were derived from tissues *Liver*, *Blood* and *Brain* via 3 assay types *DNase1*, *H3K27ac* and *H3K4me3*. It is reasonable to assume that for a given eQTL study, particular tissues or cell types are more relevant than others. We model this by introducing a variable υ for each tissue (or cell type) that affects the prior distribution of only those elements of $\boldsymbol{\omega}$ that are derived from this tissue, e.g. υ_{Liver} only affects elements of $\boldsymbol{\omega}$ tied to experiments performed in liver. Analogously to tissue, we model different priors for various for assay types. Shown is the resulting network of influences of the variable $\boldsymbol{\upsilon}_{tissue}$, $\boldsymbol{\upsilon}_{assay}$ on $\boldsymbol{\omega}$. (For visual clarity, we used different colours and linetypes to denote the dependence structure. Also, we used the actual group names as indices, while in the main text, elements of $\boldsymbol{\upsilon}$'s and $\boldsymbol{\omega}$ are indexed by natural numbers).

Typically, directed annotations are grouped by their cell type or assay type. *BAGEA* can use this grouping structure in order to select groups of annotations that are useful for predicting gene expression (Fig 1B). *BAGEA* selects annotation groups via a modeling strategy that yields sparsity on the annotation group level similar to the group lasso [13]. In *BAGEA*, this grouping strategy is implemented by partitioning annotations into multiple meta-annotations (such as different cell types, assay types etc.). When using this partitioning mechanism, *BAGEA* includes an extra random variable vector $\boldsymbol{\upsilon}$ of the same length as the number of elements in the partition structure (e.g. the number of cell types, or the number of assay types) (See Methods as well as Fig 1B for an illustrative example). The kth element of $\boldsymbol{\upsilon}$, υ_k, controls the variance of the effect sizes for annotations that fall into partitioning group k. Specifically, υ_k is proportional to the inverse of the variance of the respective elements of $\boldsymbol{\omega}$. $\boldsymbol{\upsilon}_k^{-1}$ is therefore called the *variance modifier* of annotation partition element k (see Methods).

Importantly, the model can be reformulated in terms of the summary statistics $\boldsymbol{z}_j = \boldsymbol{X}_j^T \boldsymbol{y}_j / \sqrt{n}$ and LD matrices $\boldsymbol{\Sigma}_j = \boldsymbol{X}_j^T \boldsymbol{X}_j / n$. The reformulation enables the application of *BAGEA* to studies for which only summary statistics are available, by estimating $\boldsymbol{\Sigma}_j$ from external sources (see Methods).

Evaluation Strategy for Model Fit

We developed an approach to evaluate the performance of *BAGEA* when fitting directed annotations to genotype and gene expression data. An important feature of *BAGEA* is that its results can be used to predict gene expression for a gene without using any expression data for that gene, but rather using genotypes and genome annotations whose weights are fitted from other genes. We can therefore validate *BAGEA* by training it on gene expression data for one set of genes, and then calculating the extent to which the trained model predicts gene expression for other genes.

We propose a so-called directed predictor $\hat{\boldsymbol{\mu}}_j$, which predicts gene expression for gene j based on knowledge of directed annotations and genotype for gene j. Using the

same notation as in equations (1) and (2), the predictor $\hat{\boldsymbol{\mu}}_j$ is computed by

$$\hat{\boldsymbol{\mu}}_j = \boldsymbol{X}_j((\boldsymbol{F^j}\hat{\boldsymbol{\nu}}) \cdot (\boldsymbol{V^j}\hat{\boldsymbol{\omega}})) \tag{3}$$

The squared magnitude $S_j = \hat{\boldsymbol{\mu}}_j^T \hat{\boldsymbol{\mu}}_j$ measures how much gene expression variance the model attempts to explain via the predictor $\hat{\boldsymbol{\mu}}_j$. To evaluate the predictor's accuracy and degree of overfitting, we use the *directed mean squared error* $MSE_j^{dir} = (\boldsymbol{y}_j - \hat{\boldsymbol{\mu}}_j)^T(\boldsymbol{y}_j - \hat{\boldsymbol{\mu}}_j)/n$. The evaluation of the predictor is performed on a set of genes independent of the ones used to estimate $\boldsymbol{\omega}$ and $\boldsymbol{\nu}$.

Inspection of equation (3) shows that we can reformulate the right hand side in terms summary statistics $\boldsymbol{z}_j = \boldsymbol{X}_j^T \boldsymbol{y}_j/\sqrt{n}$, LD matrices $\boldsymbol{\Sigma}_j = \boldsymbol{X}_j^T \boldsymbol{X}_j/n$, and estimated directed effect of SNPs $\hat{\boldsymbol{\eta}}_j = (\boldsymbol{F^j}\hat{\boldsymbol{\nu}}) \cdot (\boldsymbol{V^j}\hat{\boldsymbol{\omega}})$, i.e. we can write $MSE_j^{dir} = 1 - 2\hat{\boldsymbol{\eta}}_{\boldsymbol{j}}\boldsymbol{z}_j/\sqrt{n} + \hat{\boldsymbol{\eta}}_j^T \boldsymbol{\Sigma}_j \hat{\boldsymbol{\eta}}_j$ if we assume that $\boldsymbol{y}^T\boldsymbol{y} = n$. In principle, the reformulation allows us to calculate a predictor's directed mean squared error, even if only summary statistics are available, by approximating $\boldsymbol{\Sigma}_j$ from external sources.

Directed Annotations Derived from Blood can Partially Explain *cis*-eQTLs in Monocytes

We used *BAGEA* to determine the extent to which annotations can predict gene expression in *CD14* positive monocytes. To this end, we aggregated data from two eQTL studies on expression genetics in *CD14* positive monocytes [14, 15]. For directed annotations, we used predictions of genetic variant effects on epigenetic marks (12 different histone mark assays and DNase1 with 4 different peak calling strategies) in various blood-derived cell types from the pre-trained *Expecto* model. *Expecto* is a deep learning framework that predicts epigenetic marks based on sequence context and performs *in silico* mutagenesis to evaluate the consequences of sequence variants [7]. *Expecto* yielded 2002 directed annotations of which 253 were from blood related celltypes. These are referred to as the *Blood* annotation subset in this paper. We partitioned these directed annotations by cell type and assay type, respectively, and modeled separate prior variance terms for each partition (Fig 1A).

To train *BAGEA*, we used gene expression data from human chromosomes 1 through 15. Only genes that had a SNP in *cis* showing a signficant assocation with a p-value lower than 10^{-10} were included. To test model fit, we predicted expression for genes on chromosomes 16 through 22 with a top nominal *cis*-eQTL p-value below 10^{-10}. Specifically, we used the model fit on the training set to derive the estimates $\hat{\boldsymbol{\omega}}$ and $\hat{\boldsymbol{\nu}}$ (see Equation 2). We then used these estimates to calculate the directed predictors $\hat{\boldsymbol{\mu}}_j$ for genes on the test set (see Equation 3). To assess the predictive power of $\hat{\boldsymbol{\mu}}_j$, we calculated MSE_j^{dir} for every gene in the test set.

We observed that directed genome annotations can partially explain gene expression variance (Fig 2). The average MSE^{dir} across all genes was 99.5%, which was significantly smaller than 100% (as evaluated by bootstrap sampling genes; p-value smaller than 10^{-4}). MSE_j^{dir} showed a dependence on predictor size S_j (where $S_j = \hat{\boldsymbol{\mu}}_j^T \hat{\boldsymbol{\mu}}_j$), such that for the top quartile of genes when ranked by S_j, the directed component was estimated to predict 1% to 3% of expression variance (Fig 2A). For each gene, the variance explained is bounded by the additive genetic variance component in *cis* which is typically much lower than 100%. We estimated the variance of expression explained for each gene in *cis* in an unbiased way via Haseman-Elston (HE) regression [16]. This approach suggested that around 6.6% of the total genetic variance in *cis* was explained by the externally fitted directed component $\hat{\boldsymbol{\mu}}_j$ for genes in the top quartile w.r.t S_j (Fig 2B). We further tested the impact of the distance modifier by constraining all elements of $\hat{\boldsymbol{\nu}}$ (except the intercept element) to zero, showing that the

modeling the distance modifier leads to higher predictive power (S1 Fig). These results show that *BAGEA* can be used to model how sequence changes affect gene expression.

Fig 2. *Gene expression variance can be partially explained by directed genome annotations.*
The BAGEA model was fitted on genes in the training set (all genes on chromosomes 1 through 15) using monocyte eQTL data on genes with a top nominal p-value below 10^{-10}, and with Expecto-derived directed annotations. Expecto includes 2002 total annotations, of which one of two subsets were used: 253 annotations derived from histone and DNase1 assays in a blood related cell types (*Blood*), or, alternatively, 690 annotations derived from TF ChIP-Seq (*TF*). For each gene j in the test set (all genes on chromosomes 16 through 22 with a top nominal p-value below 10^{-10}), we calculated the directed predictor of expression $\hat{\boldsymbol{\mu}}_j$. As a measure of a predictor's size, we use its squared magnitude $S_j = \hat{\boldsymbol{\mu}}_j^T \hat{\boldsymbol{\mu}}_j$. To evaluate the predictor's performance, we calculated MSE_j^{dir}, the mean squared error (MSE) when predicting gene expression $\boldsymbol{y}_j$ from $\hat{\boldsymbol{\mu}}_j$. To estimate what the smallest attainable MSE_j^{dir} would be, we estimated $\sigma^2_{g_{cis}}$, the additive genetic variance in *cis* via Haseman-Elston regression per gene. (A) The relationship between the MSE of the predictor and its squared magnitude. We sorted results by predictor Size S_j and averaged MSE_j^{dir} within a sliding window containing 25% of genes and step size of 5% of data. **Averaged Directed Predictor Size** $\overline{S}$: The mean value of S_j per window on the horizontal axis; **Averaged Directed MSE** ($\overline{MSE^{dir}}$): The averaged MSE_j^{dir} of genes falling into the window on the vertical axis. The 95% confidence interval for each window was derived by bootstrapping. Most variance is explained by genes in the top quartile when ranked by S_j. (B) The relationship between MSE_j^{dir} and $\sigma^2_{g_{cis}}$ for genes in the top quartile when ranked by S_j. **Genetic Variance** ($\sigma^2_{g_{cis}}$): The estimated additive genetic variance in *cis* on the horizontal axis. **Directed MSE** (MSE_j^{dir}) on the vertical axis. 95% confidence intervals for the mean of both the MSE^{dir} and $\sigma^2_{g_{cis}}$ are represented as the corners of the red diamond (i.e. the confidence interval for the average MSE^{dir} is given by the upper and lower corner, whereas the confidence interval for the average $\sigma^2_{g_{cis}}$ is given by the right and left corner respectively). A linear regression is plotted as the blue line, with 95% confidence interval shown in grey.

Joint Modeling of cis-eQTLs and Directed Annotations Highlights Biologically Relevant Epigenetic Marks

We next evaluated if *BAGEA* can effectively be used to discover which annotations, or groups of annotations, are most predictive of gene expression. We grouped the directed annotations by cell type and assay type, and for each set of annotation groups, we modeled separate prior variance modifiers $\boldsymbol{\upsilon}^{-1}$ (Fig 1B). For each annotation group k we measured its contribution to gene expression as its estimated variance modifier υ_k^{-1} (See Model Overview). For the monocyte data, *BAGEA* estimated the largest variance modifiers for annotations from *DNase1* as well as *H3K27ac* and *H3K4me3* assays (Fig 3A). This observation is consistent with results from a previous method, using undirected annotations, suggesting that SNPs with an effect on gene expression are enriched in open chromatin (*DNase1*), activated enhancers and promoters (*H3K27Ac*, *H3K4me3*) [17]. Across cell type annotations, *BAGEA* estimated the largest variance modifiers for annotations from two blood cell types that were both *CD14* positive (Fig 3B). This observation matches our expectations because the cells in the underlying expression data were derived from *CD14* positive cells [14, 15]. Across all tested pairs of

assays and cell types, *BAGEA* estimated the largest positive effect sizes for annotations from *DNase1*, *H3K27ac*,*H3K4me3* assays in *CD14* positive cells (Fig 3C).

Fig 3. *BAGEA, fitted on monocyte eQTL data, selects relevant epigenetic marks and increases directional effect sizes for SNPs close to a TSS.*
Parameter estimates when applying *BAGEA* to monocyte eQTL data using as directed annotations histone and DNase1 *Expecto* predictions derived from blood-related cell types (i.e. *Blood* from Fig 2). (A) For each chromatin assay type, *BAGEA* models an **assay variance modifier** $\hat{\upsilon}^{-1}_{assay}$ that captures the extent to which that assay type is predictive of gene expression. Shown are the square roots for the assay types with the ten highest variance modifiers (from 17 assay types total). In the *BAGEA* model, *DNase1*, *H3K27Ac* and *H3K4me3* assays have largest modifiers. (B) For each cell type, *BAGEA* models a **celltype variance modifier** $\hat{\upsilon}^{-1}_{cell}$, similar to the assay variance modifier in panel a. Shown are the square roots for the cell types with the ten highest variance modifiers (out of 61 cell types). In the *BAGEA* model, *CD14* positive cells have the largest modifiers. (C) *BAGEA* reveals which experiments underlying the directed annotations that were most predictive of gene expression. **Assay Type x Cell Type**: Each experiment is a particular assay type performed in a particular cell type. **Effect Size** ($\hat{\omega}_i$, for experiment i): The BAGEA-estimated effect on gene expression. Shown are the ten largest directed annotation effect sizes. In the *BAGEA* model, the experiments using *DNase1*, *H3k27Ac* and *H3Kme4* with *CD14* positive cells have the largest effect sizes. We also see that most of the 253 annotations are estimated to have a close to zero effect. (D) Shown is the estimated **distance modifier** of the directed component, $\boldsymbol{F\hat{\nu}}$. We see a characteristic peak around the *TSS*, implying that the directed annotations are upweighted close to the *TSS*.

It is well known that eQTLs increase in intensity closer to the *TSS*. This suggests that the effects of directed annotations might also be bigger for SNPs close to the *TSS* than for SNPs that are distal. *BAGEA* models SNP distance dependence of directed annotation effects by weighting the directed annotation effect term $\boldsymbol{V^j\omega}$ across SNP's, with a distance modifier $\boldsymbol{F^j\nu}$ (see Model Overview). We next tested whether *BAGEA* estimated directed annotation effect sizes to be dependent on a SNP's distance to the *TSS*. We examined the value of a SNP's estimated distance modifier $\boldsymbol{F^j\hat{\nu}}$ against its position relative to the *TSS*. We observed a characteristic peak around the *TSS* (Fig 3D), suggesting that *BAGEA* can indeed produce a similar pattern of distance dependence for the effect sizes derived from directed annotations as for the eQTL effect sizes themselves.

We repeated this analysis with a different set of directed annotations, namely 690 *Expecto* annotations derived from transcription factor (*TF*) ChIP-Seq in any cell type. We estimated the *TF* annotation subset to be similarly predictive of gene expression as the *Blood* annotation subset (Fig 2A). Parameter estimates for $\boldsymbol{\omega}$ suggest that binding sites of the TF *MYC* in cell line *NB4* have the largest effect size on gene expression among all tested 690 annotations (S2 Fig). *NB4* is a promyelocytic leukemia cell line that can be differentiated into neutrophils or monocytes [18]. *NB4* is therefore expected to have similar expression genetics as *CD14* positive monocytes, and, given that no *TF* ChIP-Seq experiment was performed in monocyte cell lines directly, the large $\boldsymbol{\omega}$ values for *NB4* data are consistent with our expectations.

Modeling Directional Components is Robust to the Use of Summary Statistics

In many cases it is not feasible to compute LD for the population from which the summary statistics were derived (i.e., the study population), and LD has to be derived

from other sources (i.e., external genotypes) [19, 20]. The use of external genotypes allows publicly available summary statistics to be analyzed without access to restricted individual level genotype data [9]. However, LD computed on external genotypes can only approximate LD patterns of the study population. We therefore need to test the accuracy of methods when using external genotypes.

We evaluated if directed annotation effects were robust to the genetic source of LD information. We used 1000 Genomes data to compute LD [21]. We re-fit the $BAGEA$ model to monocyte data with the *Blood* annotation subset, using LD matrices derived from European 1000 Genomes data. We then compared $\boldsymbol{\omega}$ estimates when using LD from 1000 Genomes to $\boldsymbol{\omega}$ estimates when using LD from the monocyte data itself, for every annotation in the monocyte Blood data. We observed that the two approaches produced similar effect sizes with a linear regression R^2 of 97.5% and regression slope of 0.96 (S3 FigA). This suggests that directed annotation effect estimates are robust to the source of LD information. We then explored if the source of LD information affected our estimates of directed mean squared error (MSE^{dir}). To this end, we estimated MSE^{dir} on chromosomes 16 through 22 from summary statistics and external LD matrices derived from 1000 Genomes alone, and then compared these MSE^{dir} values to the original MSE^{dir} values computed with LD derived from monocyte data. We ensured that the same SNPs were included, by removing SNPs with low minor allele frequency (MAF) in either of the sets. We observed that the two sources of LD produced MSE^{dir} values that agree with each other, with a linear regression R^2 of 99.9% and regression slope of 1.002 (S3 FigB).

Predictive Models of GTEx Summary Statistics Make Use of Biologically Relevant Annotations

Having established that $BAGEA$ performs well when using summary statistics, we next determined if $BAGEA$ can identify relevant directed annotations for empirical data for which summary statistics are available but genotypes are not. Specifically, we fit $BAGEA$ on summary statistics for eQTL studies of 13 tissues produced by the GTEx consortium with a sample size of at least 300 for each study [9]. We additionally supplemented this set with results for Lymphoblastoid cell lines (LCL) derived from a meta-analysis of GTEx and GEAUVADIS [11]. Because GTEx gathered eQTLs in complex tissues and sampled fewer individuals than were sampled in the monocyte studies, we expected lower power to produce robust parameter estimates. We therefore used different parameter values than in our monocyte analysis, including genes with top nominal *cis*-eQTL p-value lower than 10^{-7}. We fitted models using *Expecto* derived annotation for all 1187 histone or DNase1 annotations derived from Roadmap consortium data [22].

We again split genes into training and test set, fitting $BAGEA$ on the training set and building directed expression predictors $\hat{\boldsymbol{\mu}}_j$ for all genes in the test set. We observed that the average MSE^{dir} per data set was variable across GTEx data sets ranging from 100% to below 98.5%(Fig 4A). We again saw that increased directed predictor magnitude tended to decrease MSE^{dir}. For instance in fibroblast, the quarter of the genes with the highest directed predictor magnitude had an average MSE^{dir} of 97.2%, whereas the quarter with the lowest directed predictor magnitude had an average MSE^{dir} close to 100% (Fig 4B).

To mitigate the impact of limited power during variable selection, we additionally fit models without splitting chromosomes into test and validation sets. The distribution of effect sizes of the directional annotations revealed a bias towards positive values (S4 Fig). Focusing on the largest positive effect sizes(top ten or $\hat{\omega}_i > 0.06$), we saw many biologically consistent pairings between the tissue assayed by GTEx via eQTL and the

Fig 4. *Directed annotations parially explain gene expression variance in GTEx.* The BAGEA model was fit using various GTEx eQTL data (supplemented with GEAUVADIS eQTL data) and with Expecto-derived directed annotations on genes in the trainig set (chr1,..,chr15) with a top nominal p-value$< 10^{-7}$. Expecto includes 2002 total annotations, of which histone and DNase1 annotations from Roadmap were used (1187 annotations in total). For each gene j in the test set (chr16,..,chr22 and top nominal p-value$< 10^{-7}$), we calculated an approximate version of S_j, the squared magnitude of the directed predictor $\hat{\boldsymbol{\mu}}_j$, where the approximation uses external LD information. Further, we calculated an approximate version of MSE_j^{dir}, the mean squared error (MSE) when predicting gene expression $\boldsymbol{y}_j$ from $\hat{\boldsymbol{\mu}}_j$. (A) Displayed is the average (approximated) MSE_j^{dir} across all genes for each GTEx experiment. 95% Confidence intervals are computed by bootstrap sampling. (B) Displayed is the relationship between the MSE of the predictor and its squared magnitude for the four GTEx experiments with the lowest average MSE_j^{dir}. We sorted results by predictor size S_j and averaged MSE_j^{dir} within a sliding window containing 25% of genes within the window and step size of 5% of data. **Averaged Directed Predictor Size** $\overline{S}$: The mean value of S_j per window on the horizontal axis; **Averaged Directed MSE** ($\overline{MSE^{dir}}$): The averaged MSE_j^{dir} of genes falling into the window on the vertical axis. The 95% confidence interval for each window was derived by bootstrap sampling. We see that most variance is explained by genes in the top quartile w.r.t. S_j.

tissue assayed by roadmap for epigenetic marks (Fig 5A). While some of the pairings are obvious from the annotation names themselves (such as correct pairings for lymphoblastoid cells, lung and adipose tissues) others were suprising yet on closer inspection, turned out to be consistent with the biological literature. For instance bone marrow derived mesenchymal stem cells (*BMD MSC*) are paired with fibroblast. A recent study found no functional differences between the two cell types leading the authors to support a longstanding opinion in the field that these two cell types should be classified as the same [23, 24]. The pairing between Esophagus Mucosa and keratinocytes can be explained by the fact that the Esophagus Mucosa is mainly composed of squamous cells, i.e. keratinocytes [25, 26]. The pairing between tibial artery and *BMD MSC* can be explained by the fact that fibroblasts are the main component of vascular adventitia [27]. Our model also paired tibial nerve and muscle, which seems physiologically the least biologically consistent among the ten pairings. When looking at the largest negative values, we saw some of the same tissue pairings repeated, with only one pairing with effect size $\hat{\omega}_i$ smaller than -0.06 for the pairing between fibroblasts and *BMD MSC*) (S5 Fig). When looking at the variance modifier estimates for the different assay types, we saw that *DNAse1* and *H3K27ac* epigenetic marks were ranked consistently highly (Fig 5B). Interestingly, among various annotations derived from the same *DNase1* experiments, some performed consistently better than others: *DNase1* peak call annotations outperformed *DNase1* hotspots calls. These two annotation types make use of the same underlying DNAse-seq data, but use different data processing pipelines [28]. *BAGEA* makes a clear choice which should be preferred to model sequence impacts on gene expression.

Discussion

Here we introduced a new method, named *Bayesian Annotation Guided eQTL Analysis* (*BAGEA*). *BAGEA* integrates directed and undirected genome annotations with eQTL data in a variational Bayesian framework to build predictive models of gene expression.

Fig 5. *Model fit for GTEx summary statistics selects directional annotations mainly from biologically consistent cell types.*
Shown here are various parameter estimates from fitting 13 different GTEx eQTL summary statistics data (supplemented with GEAUVADIS eQTL data) using histone and DNase1 *Expecto* predictions derived from Roadmap (1187 annotations). (A) *BAGEA* reveals the experiments underlying the directed annotations that are most predictive of gene expression. **GTEx x Roadmap(Rm)**: Each GTEx eQTL data set highlights particular Roadmap annotations . Shown here are the 10 largest positive effect sizes across all eQTL and annotation pairings. **Effect Size**: The estimate of $\hat{\omega}_i$ for experiment i. (B) For each chromatin assay type, BAGEA models an assay variance modifier $\hat{v}_{assay}^{-1}$ that expresses the extent to which that assay type is predictive of gene expression. Shown here is the distribution of the square roots of the assay variance modifier for any given assay type across all 13 GTEx eQTL data sets. Results are sorted by the maximal value achieved for each assay type and only the 10 highest scoring assay types are shown.

include imputed?

We applied this method to eQTL results from *CD14* positive monocytes as follows: First, we derived directed annotations by predicting functional impacts on epigenetic marks for all common SNPs using the pre-trained *Expecto* deep neural net [7]. Second, from these *Expecto* results, we extracted two annotation subsets of particular interest: histone ChIP-Seq and DNase1 in blood-derived cell types (the *Blood* annotation subset), and TF ChIP-Seq in any cell type (the *TF* annotation subset). We then ran *BAGEA* on both annotation subsets separately, while allowing the effect of the directed annotations to depend on the distance to the *TSS*. We tested whether the model had explanatory power with a training and test protocol (i.e. explanatory power was estimated on genes that were excluded from training). We saw that the directed component $\boldsymbol{\mu}$ of the model explained part of the gene expression variance in a statistically significant manner (Figure 2A). For genes with a strong cis-eQTL (p-value$< 10^{-10}$) and in the top quartile for $\boldsymbol{\mu}^{\boldsymbol{T}}\boldsymbol{\mu}$, we estimated that the *Blood* derived directed component explained 6.6% of total additive genetic variance in *cis* (Figure 2B). Importantly, *BAGEA* prioritized annotations that cohere with widely accepted biological knowledge and are supported by existing literature (Figure 3).

Next, we investigated to which extent the model fit was affected when the LD information was approximated via reference genomes. We observed agreement between the results in terms of the directed component, suggesting that the use of eQTL summary statistics together with external LD data is justified (S3 Fig). We therefore used *BAGEA* to analyze eQTL summary statistics results from GTEx. To accommodate the wide range of tissues explored in GTEx, we expanded the number of directed annotations used in the fitting process to over a thousand. While for some tissues, the analysis strategy was underpowered to derive a predictive model of gene expression from directed annotations, others had a significant fraction of gene expression explained by directed annotations (Figure 4). Many of the directed annotations *BAGEA* selected, were derived from tissues that were biologically related to the original tissue of the eQTL studies (Figure 5A). Additionally, we observed that *DNAse1* and *H3K27ac* epigenetic marks were selected across many different eQTL studies (Figure 5B).

BAGEA belongs to a class of models that allow the prior probability distribution of a SNP's effect size to vary based on the genome annotations with which it overlaps [17, 29, 30]. These prior models explored the impact of undirected annotations. While *BAGEA* can model undirected annotations, the main novelty comes from the concomitant modeling of directed and undirected annotations as well as interactions thereof. Using directed annotations to explain natural variation in phenotypes was also recently proposed by both *Zou et al.* and *Reshef et al.*, albeit with different modeling

philosophies [7, 10]. *Zou et al.* use a model that predicts expression from chromatin patterns directly. This has the advantage that genotype data is not needed. However, this method does not model the causal impact of epigenetic marks on expression levels but rather correlations, potentially negatively impacting modeling accuracy. *Reshef et al.*'s LD profile regression method has more similarities to *BAGEA* as it can also be used to analyze directed annotations and eQTL summary statistics. However, the method is geared towards multiple hypothesis testing rather than high predictive accuracy. Compared to *BAGEA*, the fitted model is simpler allowing for fast analysis of large collections of data. The increased speed comes at the cost of not being able to model certain features like interactions of directed and undirected annotations (such as distance to *TSS*). *BAGEA* uses a modeling approach that has both prediction and interpretability in mind. It allows for more complex model features while it is still useful for revealing relevant biology. Indeed, when using BAGEA on various eQTL datasets, *BAGEA* highlighted many relevant cell types. Further, allowing the directed component to depend on the distance to the *TSS* improved the model fit.

There are at least two drawbacks to *BAGEA*'s model complexity. First, there is a substantial computational cost to fit the model. To mitigate this issue, we used various computational strategies such as fast matrix inversion of approximated LD matrices and parallelization. Second, variational model fitting approach does not provide confidence intervals. While it does provide credibility intervals, the approximative nature of mean field variational inference makes these credibility intervals often unreliable [31]. In our analysis, we opted for evaluating statistical significance of the model results by using a training and test protocol.

Future research could investigate whether using a different variational approximation rather than the mean field approximation provides better estimates of the true credibility intervals. We estimated the extent to which epigenetic marks are able to predict the genetic component of gene expression in *cis*. Our results show that while the current generation of directed annotations can partially explain the genetic *cis* component of gene expression, most of the genetic *cis* component remains unexplained, indicating that there is still room for improvement. Future gains in this space will likely come from both improved directed annotations as well as improved modeling.

Methods

Model Details

We assume individual level genotype and expression data for n individuals. For gene j, we model its $n \times 1$ expression vector $\boldsymbol{y}_j$ as

$$\boldsymbol{y}_j = \boldsymbol{X}_j \boldsymbol{b}_j + \boldsymbol{\epsilon}_j, \tag{4}$$

where $\boldsymbol{X}_j$ is the $n \times m_j$ genotype matrix for the m_j SNPs surrounding gene j's *TSS*. $\boldsymbol{b}_j$ is the $m_j \times 1$ vector of SNP effect sizes and $\boldsymbol{\epsilon}_j$ the expression noise unexplained by the genotype.

$$\boldsymbol{\epsilon}_j \sim N_n(\boldsymbol{0}, (\lambda_j)^{-1} \boldsymbol{I}_n).$$

The noise term precision λ_j is modeled in a hierarchical fashion:

$$\lambda_j \sim \Gamma(\lambda_1, \lambda_2),$$

$$\lambda_2 \sim \Gamma(\rho_1, \rho_2).$$

with hyperparameters λ_1, ρ_1 and ρ_2 (while this notation is overloaded, we expect it is clear from context which parameter is meant). We model the vector of effect sizes $\boldsymbol{b}_j$ as

a multivariate normal, whose mean and covariance is affected by annotation matrices. For gene j we assume undirected $0-1$ coded annotation matrix $\boldsymbol{F^j}$ and a directed continuous annotation matrix $\boldsymbol{V^j}$, with dimensions $m_j \times q$ and $m_j \times s$ respectively. Then,

$$\boldsymbol{b}_j \sim N_{m_j}((\boldsymbol{F^j}\boldsymbol{\nu}) \cdot (\boldsymbol{V^j}\boldsymbol{\omega}), diag(\boldsymbol{\alpha}_j)^{-1}),$$

with $\boldsymbol{\alpha_j}$ being a vector of independently drawn gamma distributed random variables (the modeling is described further down). $\boldsymbol{\omega}$ and $\boldsymbol{\nu}$ are s and q dimensional multivariate normal distributed random variables respectively. $\boldsymbol{\omega}$ denotes the vector of activities of directed annotations, whereas $\boldsymbol{\nu}$ allows the overall weight that the directed annotations contribute to the effect size vary based on undirected annotations. This allows, for instance, the impact of the directed annotations to vary dependent on the distance to the *TSS*. $\boldsymbol{\omega}$ is modeled in a hierarchical fashion

$$\boldsymbol{\omega} \sim N_s(\boldsymbol{0}, diag(\boldsymbol{\delta}^{-1})),$$

where $\boldsymbol{\delta}$ is again modeled as a random variable. The choice of model for $\boldsymbol{\delta}$ enables the implementation of a grouping structure on the directional annotations (in our application, these groupings are the assay used to derive the annotation and the cell type in which the assay was performed). We allow the model to fit differences in prior variances based on group membership. Thereby, entire groups of directional annotation effects are shrunk to zero (akin to the group lasso [13]). Let $\boldsymbol{d^j}$ be a positive integer vector of length s taking h_j different values, i.e $\boldsymbol{d^j}$ partitions the vector of directed annotations into h_j groups (in this context, $j = 1, .., w$ runs over the meta-annotations, e.g. if the modeled meta-annotations are cell type and assay type, j can either take the value one or two). Let $\boldsymbol{\upsilon^j}$ be a random vector of length h_j (i.e. these are the group specific weights). Then,

$$\delta_i = \prod_{j=1}^{w} \upsilon^j_{\boldsymbol{d}^j_i},$$

$$\upsilon^j_k = \Gamma(\chi_{1j}, \chi_{2j}),$$

with hyperparameter χ_{1j}. χ_{2j} is modeled as

$$\chi_{2j} \sim \Gamma(\zeta_1, \zeta_2),$$

with hyperparameters ζ_1 and ζ_2.

$\boldsymbol{\nu}$ is modeled as

$$\boldsymbol{\nu} \sim N_q(\boldsymbol{c}, diag(\boldsymbol{p})^{-1}),$$

where $\boldsymbol{p}$ and $\boldsymbol{c}$ are hyperparameter vectors of length q.

The vector of precisions of the effect size vector $\boldsymbol{\alpha}$ is modeled as

$$\alpha_{ij} \sim \Gamma(\gamma_1, \kappa_j \gamma_{ij}),$$

where γ_1 is a hyperparameter. Note that letting the precision for each SNP vary leads to sparse estimates for $\boldsymbol{b}_j$; this is akin to automatic relevance determination (ARD) regression [12]. κ_j is a genewise parameter modeled in a hierarchical fashion

$$\kappa_j \sim \Gamma(\tau_1, \tau_2),$$

$$\tau_2 \sim \Gamma(\xi_1, \xi_2),$$

where τ_1, ξ_1 and ξ_2 are hyperparameters. To model γ_{ij}, we again make use of annotation matrices. For gene j assume undirected $0-1$ coded annotation matrix $\boldsymbol{C^j}$ of dimension $m \times t$. Then the SNP-wise precision modifier γ_{ij} is modeled as

$$\gamma_{ij} = \prod_{k:\boldsymbol{C}^{\boldsymbol{j}}_{ik}=1} a_k$$

where $C_{ik}^{j} = 1$ if annotation k is active at index i in gene region j. Further,

$$a_k = \Gamma(\phi_1, \phi_2),$$

where ϕ_1 and ϕ_2 are hyperparameters.

Summary statistics adaptation.

Instead of using individual level genotype and expression data, we can reformulate the model for the use of summary statistics. Multiplying equation 4 with $\frac{1}{\sqrt{n}}\boldsymbol{X^T}$ gives

$$\frac{1}{\sqrt{n}}\boldsymbol{X^T y}_j = \frac{1}{\sqrt{n}}\boldsymbol{X^T X}_j \boldsymbol{b}_j + \frac{1}{\sqrt{n}}\boldsymbol{X^T \epsilon}_j.$$

A natural model to use with summary statistics is therefore,

$$\boldsymbol{z}_j = \sqrt{n}\boldsymbol{\Sigma}_j \boldsymbol{b}_j + \boldsymbol{\epsilon}'_j,$$

where $\boldsymbol{z}_j$ is the vector of summary statistics, $\boldsymbol{\Sigma}_j$ is the LD matrix and $\boldsymbol{\epsilon}'_j \sim N_m(\boldsymbol{0}, \lambda_j^{-1}\boldsymbol{\Sigma}_j)$. $\boldsymbol{\Sigma}_j$ can be approximated from external sources such as 1KG [21]. Alternatively, we can use an approximate and regularized version of the empirical LD matrix (see below).

Model fitting

The model was fit using variational bayes approach [31]. As the model is in the conjugate exponential family, we can use the variational message passing strategy [32]. For detailed updating steps see S1 Appendix. Naive updates can be prohibitively expensive due to the requirement to invert many large matrices of the form $(c\boldsymbol{X^T X} + \boldsymbol{D_\alpha})$, where c is a constant and $\boldsymbol{D_\alpha}$ is a diagonal matrix. To speed up computation, we can approximate the LD matrix $c\boldsymbol{X^T X}$ with a low rank approximation $\boldsymbol{A_t^T A_t}$, where $\boldsymbol{A_t}$ is a $t \times m$ matrix with $t < m$. This allows us to speed up a time critical matrix inversion step.

$$(c\boldsymbol{X}^T\boldsymbol{X} + \boldsymbol{D_\alpha})^{-1} \approx \boldsymbol{D_\alpha}^{-1} - \boldsymbol{D_\alpha}^{-1}\boldsymbol{A_t}(\boldsymbol{I_t} + \boldsymbol{A_t^T}\boldsymbol{D_\alpha}^{-1}\boldsymbol{A_t})^{-1}\boldsymbol{A_t^T}\boldsymbol{D_\alpha}^{-1}.$$

If $\boldsymbol{X}$ is already low rank, it is computationally advantageous to use an $\boldsymbol{A_t}$ s.t. $c\boldsymbol{X^T X} = \boldsymbol{A_t^T A_t}$. If $\boldsymbol{A_t^T A_t}$ deviates from $c\boldsymbol{X^T X}$, we need to use the summary statistics formulation to avoid convergence issues. For more detail, see S1 Appendix.

Deriving Annotations

For common SNPs (minor allele frequency (MAF) above 2.5% in the 1000 Genomes European population [21]), we ran the *Expecto* model to predict the effect of the variant on epigenetic marks [7]. For each SNP we predicted the epigenetic effects within the 200 bp region encompassing it. For most SNPs the effects are very close to zero, allowing us to sparsify the results. Absolute effects smaller than 0.008 were set to zero and all other effects were shrunk towards zero by 0.008 via $x_{new} = x - 0.008 \cdot sgn(x)$. Next, results for both strands were averaged and the shrinking procedure repeated with a threshold of 0.008. This yielded matrix with 98.4% of entries zero. The directed annotations were then scaled to have all the same 2-norm. The magnitude of the 2-norm was set to the average of the unscaled 2-norms. These were the directed annotations used in *BAGEA*.

For undirected annotations, we used upstream and downstream distances to the *TSS*. Distance to *TSS* annotations as well as SNP positional annotations were downloaded from the UCSC genome annotation database with SNP and gene annotations taken from the *refGene* and *snp147Common* tables respectively (see link below) [33].

cis-eQTL data sets

For monocyte eQTL data, we used two preprocessed monocyte datasets with a combined sample size of 1176 (418 from *Fairfax et al.* and 758 from *Rotival et al.* respectively) [14, 15]. Expression matrices were quantile normalized and 10 PEER factors as well as 5 genotype PCs removed [34]. Genotype data was quality control filtered (4% SNP level missingness; 5% individual level missingness; Hardy-Weinberg p-value above 10^{-13} relatedness below 0.1875) and imputed using the human genome reference panel [35].

We further downloaded eQTL summary statistics for various tissues produced by the *GTEx* project if the number of samples was above 300 individuals [9]. Additionally, for LCL, we meta-analyzed eQTL summary statistics released for 117 samples by *GTEx* with summary statistics derived from 358 European PEER-controlled samples collected as part of the *GEUVADIS* study [11].

Running *BAGEA*

For the monocyte eQTL analysis, *BAGEA* was run with default hyperparameter settings (see S1 Appendix). Genotypes within a window of 150KB around a gene's *TSS* were used to construct a genewise LD matrix. Each Genewise LD matrix was approximated via singular value decompostion with a low rank symmetric matrix of equal top eigenvalues and eigenvectors, such that the trace of the approximation matrix was at least 99% of the trace of the original LD matrix. Then, a scaled identity matrix was added such that the trace of the resulting matrix was equal to the trace of the original LD matrix. As undirected annotations, distance windows around the TSS (50KB, 20KB, 10KB, 5KB, 2KB, 1KB, 0.5KB, 0.25KB) split into upstream and downstream windows were used. To analyse summary statistics with *BAGEA*, LD was approximated via 1KG European samples. Variant, where the reference allele in 1KG did not agree with the reference allele in the UCSC SNP annotation table, were removed. Reference 1KG LD matrices were calculated and replaced with low rank approximation with 95% of the matrix trace kept, anlagously to the above procedure. For all GTEx summary statistics analysis, default hyperparameter settings where used except for c which was set to *0.3* instead of *0* to yield consistently positive signs for ν estimates. BAGEA was run for 300 iterations in each analysis.

1 URLs

Code to run *BAGEA* can be found at https://github.com/dlampart/bagea

Links to publicly available external data sources are as follows:

- UCSC: http://hgdownload.cse.ucsc.edu/goldenpath/hg19/database/
- EXPECTO: https://github.com/FunctionLab/ExPecto/
- 1KG: ftp://ftp-trace.ncbi.nih.gov/1000genomes/ftp/release/20130502/
- GTEX: https://gtexportal.org/home/datasets
- GEUVADIS: ftp://ftp.ebi.ac.uk/pub/databases/microarray/data/experiment/GEUV/E-GEUV-3/analysis_results/
- First monocyte study: https://www.ebi.ac.uk/ega/studies/EGAS00001000411
- Second monocyte study: https://www.ebi.ac.uk/ega/studies/EGAS00000000109

Author Contributions

- **Conceptualization: D.L, R.B., K.H., T.G.B. V.H.S.**
- **Methodology: D.L.**
- **Formal Analysis: D.L.**
- **Software: D.L.**
- **Project Administration: V.H.S.**
- **Funding Acquisition: A.Z.**
- **Writing: D.L, R.B., K.H., T.G.B. A.Z. V.H.S.**

Supporting information

S1 Appendix. Supporting methods.

S1 Fig. Removing distance modifier leads to lower predictive power. We used *BAGEA* to predict gene expresion for *CD14* positive monocytes using the *Blood* annotation set, analogously to Figure 2 but removing the distance modifier by constraining all elements of $\hat{\boldsymbol{\nu}}$ except the intercept element to zero (*Without Distance Modifier*). For comparison, we additionally plotted results achieved with the same data and settings except using the default prior for $\boldsymbol{\nu}$ (*With Distance Modifier*). We see substantial decrease in power when the distance dependence of the effect sizes is not modeled.

S2 Fig. Parameter estimates for the directed annotation TF subset when using BAGEA on Monocyte eQTL Data. Shown are parameter estimates from fitting monocyte eQTL data using *TF Expecto* predictions in all cell types. (A) *BAGEA* reveals the experiments underlying the directed annotations that are most predictive of gene expression. **Assay Type x Cell Type**: Each experiment is a particular assay type performed in a particular cell type. **Effect Size** ($\hat{\omega}_i$, for experiment i): The BAGEA-estimated effect on gene expression. Shown here the ten largest directed annotation effect sizes. We see *c-Myc* annotation in *NB4* dominates. (B) Shown is the estimated **distance modifier** of the directed component, $\boldsymbol{F}\hat{\boldsymbol{\nu}}$. We see a characteristic peak around the *TSS*, implying that the directed annotations are upweighted close to the *TSS*.

S3 Fig. Directed annotation effect estimates and modeling error are robust to source of LD information. (A) Shown is a comparison of estimates of the directed annotation effect vector $\boldsymbol{\omega}$ when using external reference LD information or individual level genotypes. We retrained BAGEA with the blood monocyte summary statistics using reference LD matrices from the 1000 Genomes Project (1KG). $\hat{\omega}_i$ **(1KG)**: Directed annotation effect, measured as $\boldsymbol{\omega}$ estimates from BAGEA using 1KG reference LD information. $\hat{\omega}_i$ **(Monocyte LD)**: Directed annotation effect, measured as $\boldsymbol{\omega}$ estimates from BAGEA using individual-level genotypes from the monocyte data itself (i.e. using the same genotypes as for the deriving the summary statistics). (B) To investigate the extent to which MSE_j^{dir} can be approximated using summary statistics and reference 1KG LD matrices, we calculated MSE_j^{dir} on chromosomes 16 to 22 from summary statistics of monocyte cis-eQTLs (see formula in main text). We then compared these to the original MSE_j^{dir} values that were computed using genotypes of

the monocyte data sets. The same SNPs were used in both calculations. R^2: The coefficient of determination, measuring goodness-of-fit, from a linear regression of the data shown.

S4 Fig. Histogram of directed effect sizes $\hat{\omega}$ across all 14 GTEx data sets. Displayed are estimated directed annotation effect sizes $\hat{\omega}$ for all GTEx (and GEAUVADIS) data sets, with values with absolute value below 10^{-3} removed. Shown are results when fitting on data from all autosomes.

S5 Fig. Largest negative directed annotation effect sizes for GTEx summary statistics repeat some of the same tissue pairings as large positive effect sizes. Shown are the largest negative directed annotation effect from fitting 14 different GTEx (and GEAUVADIS) eQTL summary statistics data sets using Histone and DNase1 Expecto predictions derived from Roadmap, complementing results in Fig 6.

Acknowledgements

Special thanks to Prof. Zoltan Kutalik for helpful discussions.

References

1. Pai AA, Pritchard JK, Gilad Y. The genetic and mechanistic basis for variation in gene regulation. PLoS Genet. 2015;11(1):e1004857. doi:10.1371/journal.pgen.1004857.

2. Timpson NJ, Greenwood CMT, Soranzo N, Lawson DJ, Richards JB. Genetic architecture: the shape of the genetic contribution to human traits and disease. Nat Rev Genet. 2018;19(2):110–124. doi:10.1038/nrg.2017.101.

3. Moyerbrailean GA, Kalita CA, Harvey CT, Wen X, Luca F, Pique-Regi R. Which Genetics Variants in DNase-Seq Footprints Are More Likely to Alter Binding? PLoS Genet. 2016;12(2):e1005875. doi:10.1371/journal.pgen.1005875.

4. Zhou J, Troyanskaya OG. Predicting effects of noncoding variants with deep learning-based sequence model. Nat Methods. 2015;12(10):931–4. doi:10.1038/nmeth.3547.

5. Lee D, Gorkin DU, Baker M, Strober BJ, Asoni AL, McCallion AS, et al. A method to predict the impact of regulatory variants from DNA sequence. Nat Genet. 2015;47(8):955–61. doi:10.1038/ng.3331.

6. Kelley DR, Snoek J, Rinn JL. Basset: learning the regulatory code of the accessible genome with deep convolutional neural networks. Genome Res. 2016;26(7):990–9. doi:10.1101/gr.200535.115.

7. Zhou J, Theesfeld CL, Yao K, Chen KM, Wong AK, Troyanskaya OG. Deep learning sequence-based ab initio prediction of variant effects on expression and disease risk. Nat Genet. 2018;50(8):1171–1179. doi:10.1038/s41588-018-0160-6.

8. Gusev A, Mancuso N, Won H, Kousi M, Finucane HK, Reshef Y, et al. Transcriptome-wide association study of schizophrenia and chromatin activity yields mechanistic disease insights. Nat Genet. 2018;50(4):538–548. doi:10.1038/s41588-018-0092-1.

9. Gamazon ER, Segrè AV, van de Bunt M, Wen X, Xi HS, Hormozdiari F, et al. Using an atlas of gene regulation across 44 human tissues to inform complex disease- and trait-associated variation. Nat Genet. 2018;50(7):956–967. doi:10.1038/s41588-018-0154-4.

10. Reshef YA, Finucane HK, Kelley DR, Gusev A, Kotliar D, Ulirsch JC, et al. Detecting genome-wide directional effects of transcription factor binding on polygenic disease risk. Nat Genet. 2018;50(10):1483–1493. doi:10.1038/s41588-018-0196-7.

11. Lappalainen T, Sammeth M, Friedländer MR, 't Hoen PAC, Monlong J, Rivas MA, et al. Transcriptome and genome sequencing uncovers functional variation in humans. Nature. 2013;501(7468):506–11. doi:10.1038/nature12531.

12. Bishop CM. Pattern Recognition and Machine Learning. Springer; 2006.

13. Yuan M, Lin Y. Model selection and estimation in regression with grouped variables. Journal of the Royal Statistical Society: Series B (Statistical Methodology). 2006;68(1):49–67.

14. Fairfax BP, Humburg P, Makino S, Naranbhai V, Wong D, Lau E, et al. Innate immune activity conditions the effect of regulatory variants upon monocyte gene expression. Science. 2014;343(6175):1246949. doi:10.1126/science.1246949.

15. Rotival M, Zeller T, Wild PS, Maouche S, Szymczak S, Schillert A, et al. Integrating genome-wide genetic variations and monocyte expression data reveals trans-regulated gene modules in humans. PLoS Genet. 2011;7(12):e1002367. doi:10.1371/journal.pgen.1002367.

16. Haseman JK, Elston RC. The investigation of linkage between a quantitative trait and a marker locus. Behav Genet. 1972;2(1):3–19.

17. Veyrieras JB, Kudaravalli S, Kim SY, Dermitzakis ET, Gilad Y, Stephens M, et al. High-resolution mapping of expression-QTLs yields insight into human gene regulation. PLoS Genet. 2008;4(10):e1000214. doi:10.1371/journal.pgen.1000214.

18. Clark CS, Konyer JE, Meckling KA. 1alpha,25-dihydroxyvitamin D3 and bryostatin-1 synergize to induce monocytic differentiation of NB4 acute promyelocytic leukemia cells by modulating cell cycle progression. Exp Cell Res. 2004;294(1):301–11. doi:10.1016/j.yexcr.2003.11.013.

19. Yang J, Ferreira T, Morris AP, Medland SE, Genetic Investigation of ANthropometric Traits (GIANT) Consortium, DIAbetes Genetics Replication And Meta-analysis (DIAGRAM) Consortium, et al. Conditional and joint multiple-SNP analysis of GWAS summary statistics identifies additional variants influencing complex traits. Nat Genet. 2012;44(4):369–75, S1–3. doi:10.1038/ng.2213.

20. Ehret GB, Lamparter D, Hoggart CJ, Genetic Investigation of Anthropometric Traits Consortium, Whittaker JC, Beckmann JS, et al. A multi-SNP locus-association method reveals a substantial fraction of the missing heritability. Am J Hum Genet. 2012;91(5):863–71. doi:10.1016/j.ajhg.2012.09.013.

21. 1000 Genomes Project Consortium, Auton A, Brooks LD, Durbin RM, Garrison EP, Kang HM, et al. A global reference for human genetic variation. Nature. 2015;526(7571):68–74. doi:10.1038/nature15393.

22. Roadmap Epigenomics Consortium, Kundaje A, Meuleman W, Ernst J, Bilenky M, Yen A, et al. Integrative analysis of 111 reference human epigenomes. Nature. 2015;518(7539):317–30. doi:10.1038/nature14248.

23. Hematti P. Mesenchymal stromal cells and fibroblasts: a case of mistaken identity? Cytotherapy. 2012;14(5):516–21. doi:10.3109/14653249.2012.677822.

24. Denu RA, Nemcek S, Bloom DD, Goodrich AD, Kim J, Mosher DF, et al. Fibroblasts and Mesenchymal Stromal/Stem Cells Are Phenotypically Indistinguishable. Acta Haematol. 2016;136(2):85–97. doi:10.1159/000445096.

25. Whelan KA, Muir AB, Nakagawa H. Esophageal 3D Culture Systems as Modeling Tools in Esophageal Epithelial Pathobiology and Personalized Medicine. Cell Mol Gastroenterol Hepatol. 2018;5(4):461–478. doi:10.1016/j.jcmgh.2018.01.011.

26. Doupé DP, Alcolea MP, Roshan A, Zhang G, Klein AM, Simons BD, et al. A single progenitor population switches behavior to maintain and repair esophageal epithelium. Science. 2012;337(6098):1091–3. doi:10.1126/science.1218835.

27. Di Wang H, Rätsep MT, Chapman A, Boyd R. Adventitial fibroblasts in vascular structure and function: the role of oxidative stress and beyond. Can J Physiol Pharmacol. 2010;88(3):177–86. doi:10.1139/Y10-015.

28. Thurman RE, Rynes E, Humbert R, Vierstra J, Maurano MT, Haugen E, et al. The accessible chromatin landscape of the human genome. Nature. 2012;489(7414):75–82. doi:10.1038/nature11232.

29. Gaffney DJ, Veyrieras JB, Degner JF, Pique-Regi R, Pai AA, Crawford GE, et al. Dissecting the regulatory architecture of gene expression QTLs. Genome Biol. 2012;13(1):R7. doi:10.1186/gb-2012-13-1-r7.

30. Das A, Morley M, Moravec CS, Tang WHW, Hakonarson H, MAGNet Consortium, et al. Bayesian integration of genetics and epigenetics detects causal regulatory SNPs underlying expression variability. Nat Commun. 2015;6:8555. doi:10.1038/ncomms9555.

31. Blei DM, Kucukelbir A, McAuliffe JD. Variational inference: A review for statisticians. Journal of the American Statistical Association. 2017;112(518):859–877.

32. Winn J, Bishop CM. Variational message passing. Journal of Machine Learning Research. 2005;6(Apr):661–694.

33. Haeussler M, Zweig AS, Tyner C, Speir ML, Rosenbloom KR, Raney BJ, et al. The UCSC Genome Browser database: 2019 update. Nucleic Acids Res. 2019;47(D1):D853–D858. doi:10.1093/nar/gky1095.

34. Stegle O, Parts L, Piipari M, Winn J, Durbin R. Using probabilistic estimation of expression residuals (PEER) to obtain increased power and interpretability of gene expression analyses. Nat Protoc. 2012;7(3):500–7. doi:10.1038/nprot.2011.457.

35. McCarthy S, Das S, Kretzschmar W, Delaneau O, Wood AR, Teumer A, et al. A reference panel of 64,976 haplotypes for genotype imputation. Nat Genet. 2016;48(10):1279–83. doi:10.1038/ng.3643.

Figure1

Click here to download Figure Fig1.tiff

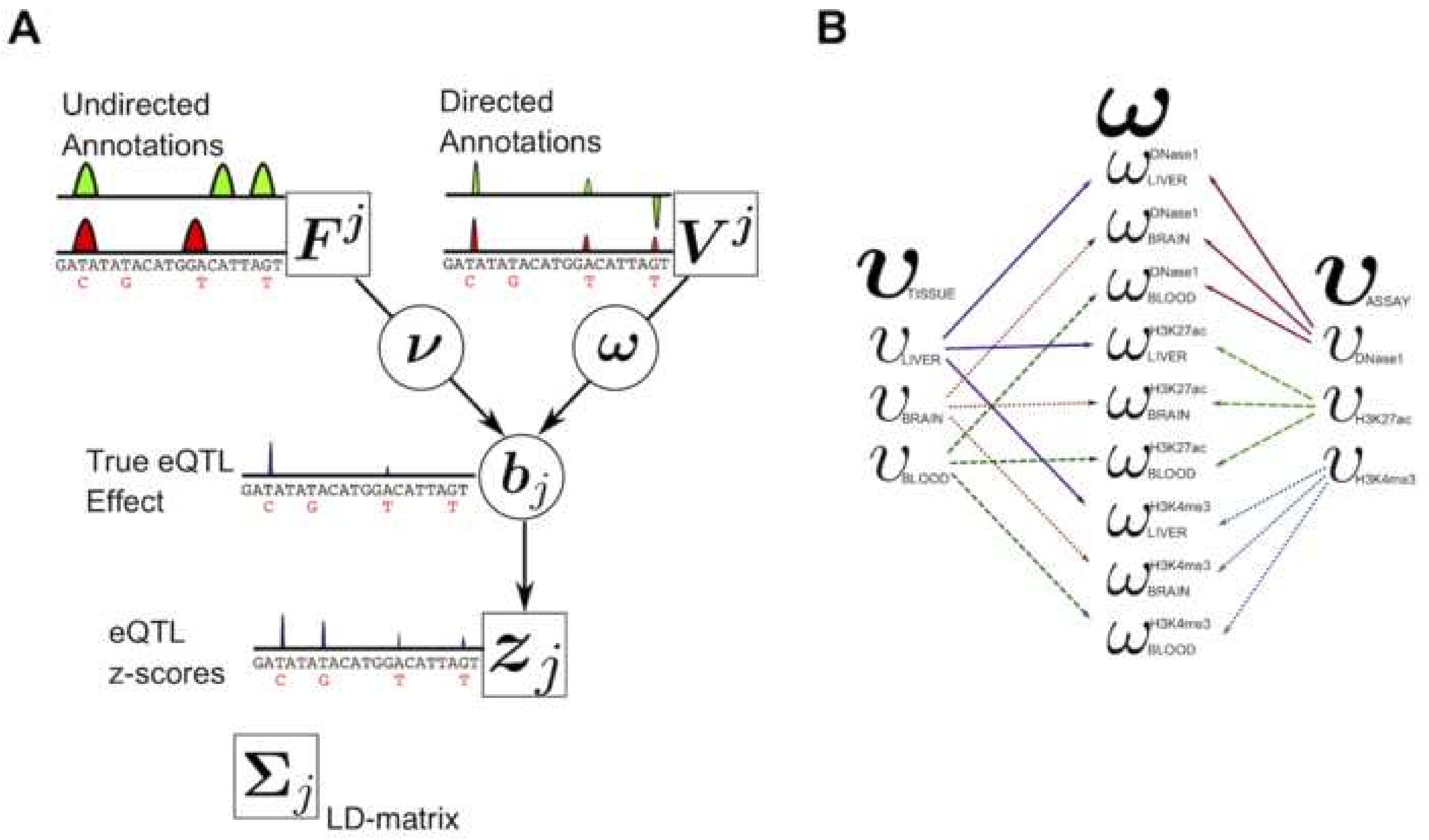

Figure2

Click here to download Figure Fig2.tiff

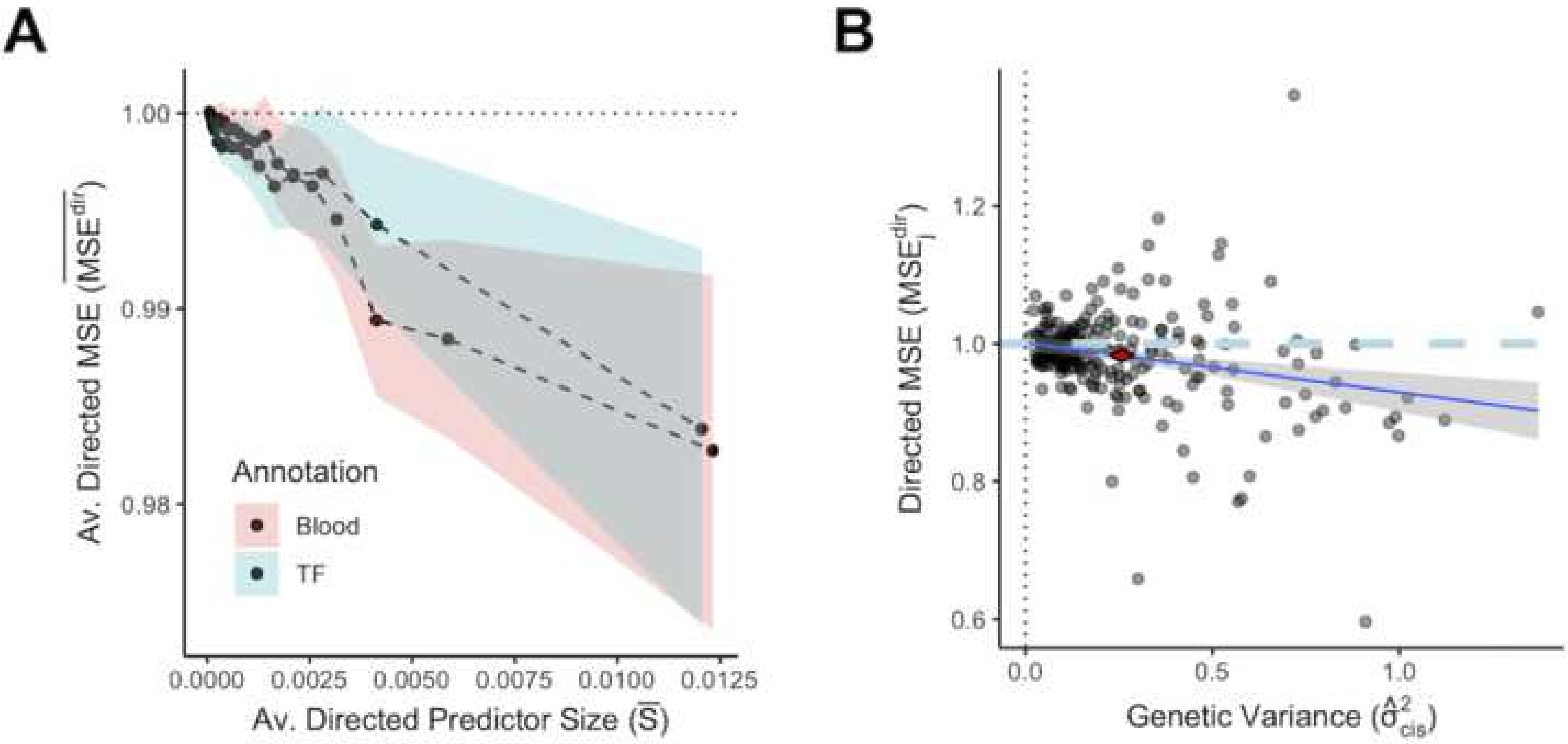

Figure3 Click here to download Figure Fig3.tiff

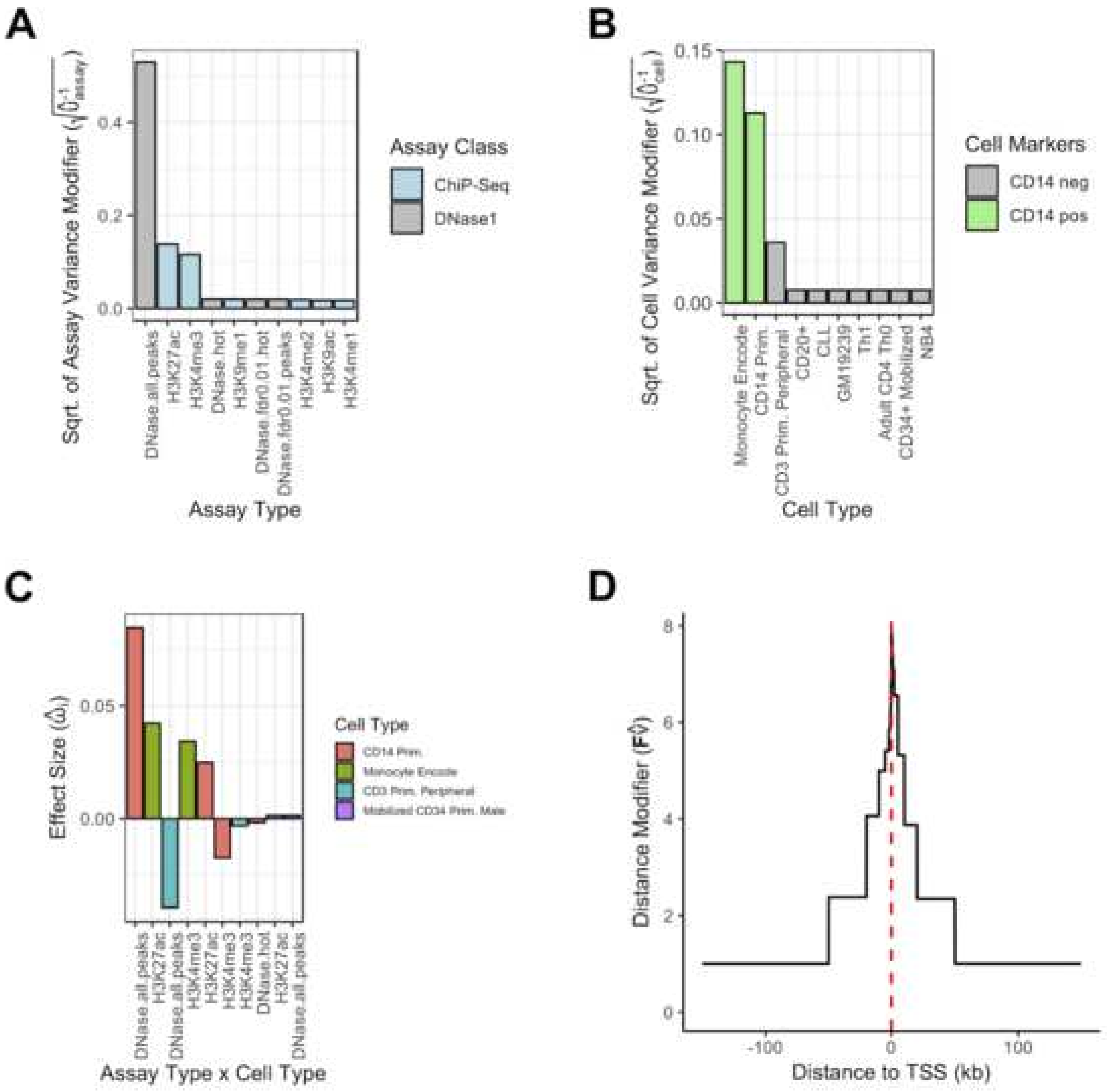

Figure4 Click here to download Figure Fig4.tiff

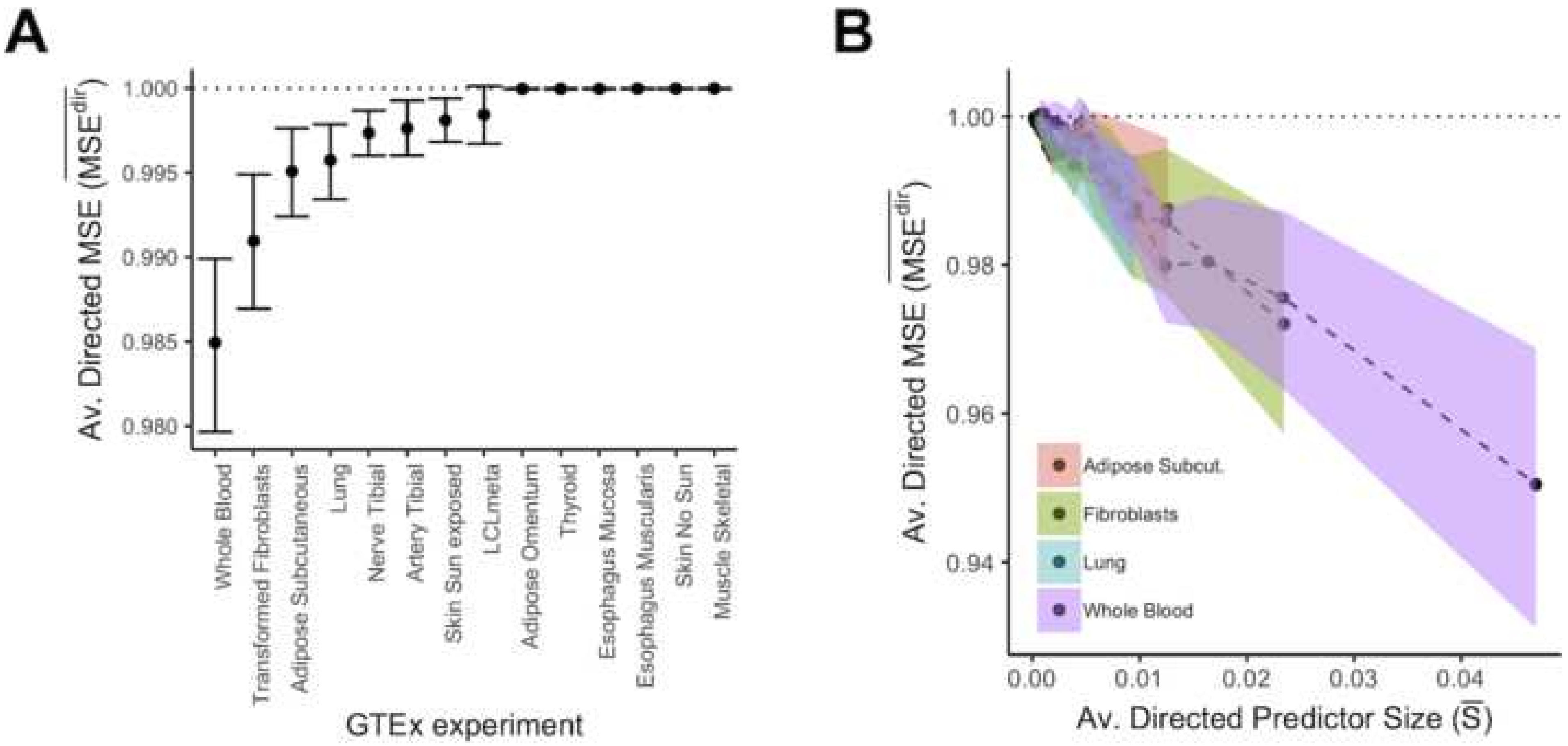

Figure5

Click here to download Figure Fig5.tiff

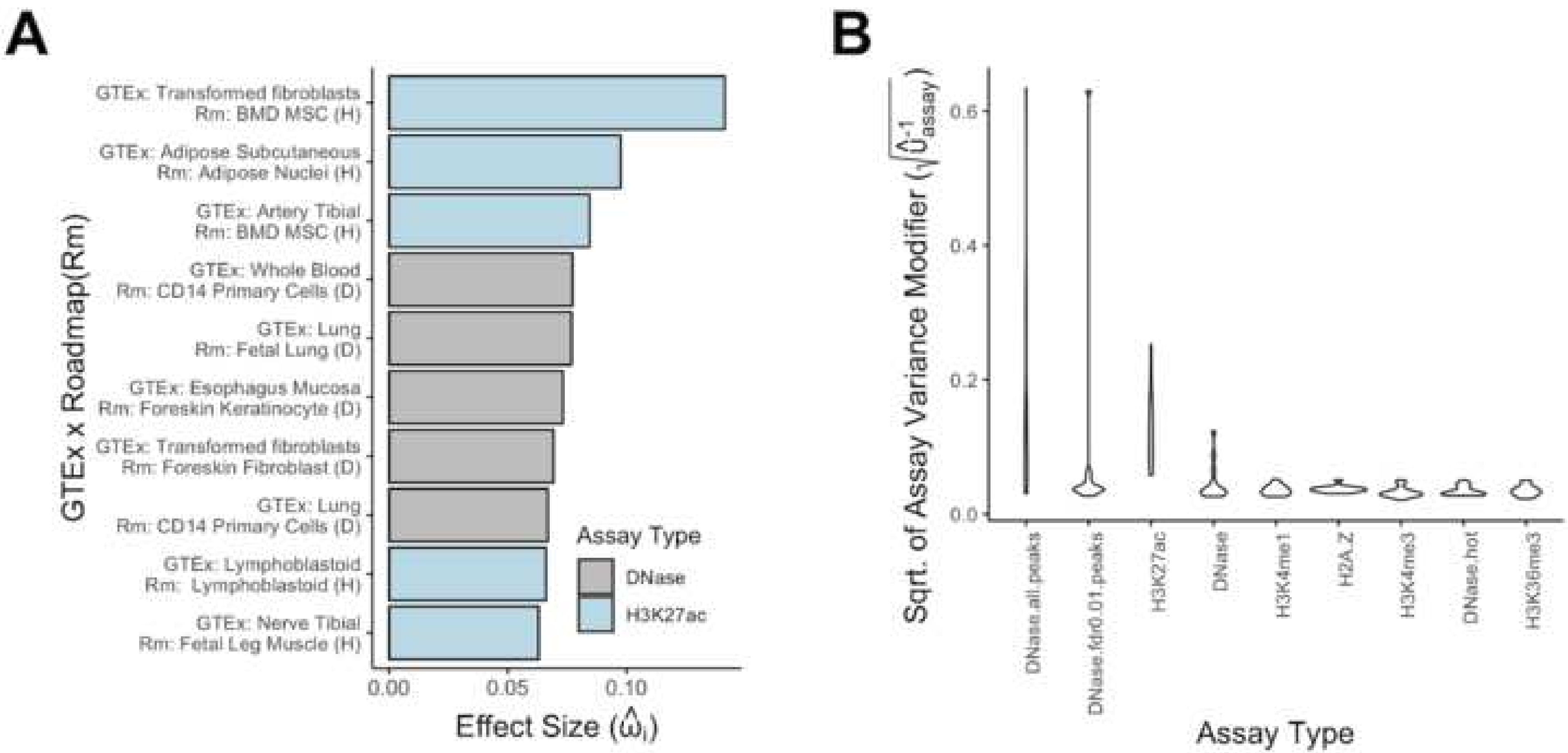

Supporting Methods

Click here to access/download
Supporting Information
supporting_methods.pdf

SupportingFigure1

Click here to access/download
Supporting Information
SupFig1.tiff

SupportingFigure2

Click here to access/download
Supporting Information
SupFig2.tiff

SupportingFigure3

Click here to access/download
Supporting Information
SupFig3.tiff

SupportingFigure4

Click here to access/download
Supporting Information
SupFig4.tiff

SupportingFigure5

Click here to access/download
Supporting Information
SupFig5.tiff
