# Supplementary material for: A framework for integrating directed and undirected annotations to build explanatory models of cis-eQTL data
Source: PLoS Comput Biol. 2020 Jun 9;16(6):e1007770. doi: 10.1371/journal.pcbi.1007770 (PMC7332077; doi:10.1371/journal.pcbi.1007770)
Supplement: Attachment — Submitted filename: cover letter _revisions.pdf [file pcbi.1007770.s023.pdf]

November 20, 2019

Roger Pique-Regi, Ph.D.
Guest Editor
PLOS Computational Biology

Weixiong Zhang, Ph.D.
Deputy Editor
PLOS Computational Biology

Dear Dr. Pique-Regi and Dr. Zhang,

We thank you for reviewing our manuscript and for your insightful comments. We found them very useful to improve the manuscript. We have incorporated the reviewer's comments, updated the manuscript accordingly, and uploaded the revisions using PLoS Editorial Manager web portal.

Some of the larger changes that were prompted by the reviewers' request are summarized here:

- We ran our BAGEA method over directed annotations derived from ENCODE Non-Histone ChiP-Seq data (mainly consisting of transcription factors). We updated Figure 5 to include results from the above analysis. We then used the results to identify transcription factors acting as repressors or activators *de novo*, an application that cannot readily be replicated by methods using undirected annotations alone.

- We added a simulation study, investigating how well we could successfully identify active annotations.

- We added comparison to results from ExPecto as well as Torus.

Additionally, with the guiding help of the reviewers comments, we added analyses and made changes throughout the manuscript to improve clarity, add context, and deepen discussions of our findings. We have listed all our changes in the subsequent pages of this letter.

Best Regards,
Victor Hanson-Smith, PhD
Head of Computational Biology
Verge Genomics

Reviewer #1:

Major issues

1. I can imagine the utility of using directed annotations to interpret gene expression, but I don't think that utility has been shown. The method prioritizes certain genomic annotations that make biological sense. But there's no comparison to the annotations that are prioritized by existing tools that use undirected annotations only. There's no comparison to a simple enrichment test of genomic annotations of eQTL variants, or a more sophisticated enrichment test using e.g. DAP/Torus. The introduction also does not include a discussion of current approaches that use undirected annotations.

We added a section to the introduction outlining the work that has been done over the years on integration of undirected annotations with eQTL data (see paragraph starting: *Approaches using directed annotations ..*).
Further, we added a comparison of BAGEA with Torus (a tool which allows to model SNP effect priors) with l_2 penalty; we ran this comparison on an additional analysis where the encode-TF annotations were used. We compared effect sizes between BAGEA and Torus (See Figure S9 as well as supplementary methods section: Torus comparison). BAGEA, employing a penalty closer in kind to l_1, had more values very close to zero distribution. We saw that distribution of annotation effect sizes estimated via Torus, was substantially shifted for non-zero bagea effects, showing that the two methods tended to prioritize similar annotations.
Additionally, to strengthen the case that BAEGA selects causal annotations, we added results from simulation experiments (See Results section titled *Exploring BAGEA's ability to identify causal marks through simulation*).

2. This paper appears to rely heavily on the ExPecto algorithm (ref 7). ExPecto is used to generate the directed annotations by calculating the difference in predicted epigenetic signals [e.g. DNase, histone marks] between the reference and alternative alleles of a SNP. These values are then entered into the BAGEA directed annotation matrix. ExPecto can already use those values to predict the effect of a given mutation on gene expression (although I don't think it takes TSS distance into account). The current method's utility is that using the ExPecto annotations, it can learn which of the annotations are relevant for genetic effects in a given dataset on a genome-wide basis. While this seems like it could be useful and interesting, it's difficult to judge without a metric for comparison (major issue #1).

While ExPecto does take TSS distance into account, its modeling approach connecting annotations to expression does not rely on genetics. Associations between chromatin marks and expression are therefore correlative. Because of this, the modeling approach assumes *a priori* that causality flows from chromatin marks to gene expression. However, recent integrative analysis, modeling causality between expression and chromatin marks, suggest that this is not always the case (see for instance Delaneau et al. 2019, showing that

expression can causally change chromatin organization close to the promoter). We expanded the section outlining this reasoning in the discussion (See Discussion paragraph starting with *Using directed annotations to explain..*)
Further, we added an explicit comparisons to results from ExPecto using model fits for the GTEx data sets that were released as part of the original ExPecto publication. (See Figure S7). This showed that BAGEA had favourable performance for genes with large directed predictors Sj. Taken together, these various comparisons strongly suggest to us that eQTL data can play a significant role in modeling of gene expression via directed annotations.

3. The prioritized annotations per dataset do not appear to offer major biological insight. The findings provided are a good "sanity check" that the expected annotations are prioritized, and there are a couple minor biological insights (e.g. bone-marrow-derived mesenchymal stem cells vs. fibroblasts, DNase1 peaks vs. hotspots). It would add to the paper if the methodology could be used to create new hypotheses about a new or unknown dataset or biological question.

We added an analysis where GTEx data was fit using directed annotations derived from ENCODE Non-HistoneChiP-Seq data, mainly consisting of transcription factors TF (see updated Figure 4). This allowed us to address the biological question: should TFs be classified as transcriptional activators or repressors (See Figure S13). We systematically checked whether our *de novo* predictions agreed with prior knowledge (as formalized in the TRRUST-db). We saw statistically significant agreement in terms of derived repressor activity measures (p-value below 10^{-4}, R^2 above 0.4). This highlights one of the benefits for using directed annotations, as such an analysis is not readily possible, using undirected annotations alone.

Minor issues

1. The authors should provide more context for Sj/MSEdir and highlight the expected values possible based on the cis genetic varition. What is the maximum expression variance that their method could have been expected to explain? The finding that "6.6% of the total genetic variance in cis was explained by the externally-fitted directed component mu_j for genes in the top quartile" helped me understand the usefulness and biological relevance of the method. I'm also curious how much genetic variance was explained by only the variants that end up being used for prediction, as 98.4% of the entries in the directed matrix were set to zero.

We added the numbers for all genes in the test set (with p-value smaller than 1E-10). On average, we saw 2.5% of the total genetic variance in cis was explained by the externally-fitted directed component mu_j (average MSE_dir 0.9945; total genetic variance in cis: 21.5%). We added those numbers to the text. We also checked the fraction of variants that did not have any active directed annotations in the (Blood-annotation subset) only 60%

were set to zero, making it unlikely that an overwhelming proportion of the genetic variance in cis explained by these variants.

2. The method introduces an intuitive way to learn the pattern of the importance of distance to TSS. However, I wonder how much this pattern varies between genes and between datasets, and I don't see any data on this. If the pattern doesn't vary much, as I imagine it might not, why not just keep Fv fixed instead of learned?

We added a plot showing variation across different dataset (See Figure S14). While there was some variability for individual fits, the general shape was always respected. While we think the general shape has to be learned initially, and fixing the shape completely might be somewhat restrictive, it might be beneficial to proceed in a stepwise fashion, and taking advantage of the Bayesian setup. In a first step, data is fit to derive the average shape across all datasets. Then, data is fit again in a second step with priors adjusted to follow the average line in a relatively narrow band. The same reasoning can be applied to other priors, for instance, priors for *H3K27Ac* or *Pol2* could be adjusted based on results from the first round. We make a short mention of this strategy in the Discussion.

3. It would be interesting to know if there are differences between genes with low MSE and high MSE. Is there anything about the high MSE genes that makes it easier to predict their gene expression using this model?

This is an interesting albeit challenging question. At this point, all we can say is that based on the distance modifier term these are genes with strong *variant effect predictors* close to the TSS, as is suggested by the correlation with low MSE and Sj.

4. There are no comments about the finding that some traditionally activating annotations (e.g. Fig 3C CD3 DNase peaks) have negative effect sizes for certain datasets. I can speculate myself, but I think it would be worth discussing.

We explicitly mention this fact and suggest a potential explanation, namely that DHS exclusive to CD14 positive cells might have bigger effect sizes (See Results subsection *Joint Modeling of cis-eQTLs and Directed Annotations Highlights Biologically Relevant Epigenetic Marks*).

5. The introduction begins by explaining the importance of understanding the effects of rare variants, but the method as implemented only uses common variants (MAF > 2.5% in EUR).

We added an outlook section to the Discussion mentioning this (See the paragraph starting "*An avenue also not explored here"*). Predicting effects for very rare variants will become interesting once more WGS eQTL studies become available. Of course this necessitates derivation of directed annotations at many more variants and given that most of the eQTL

data available does not contain calls at very rare variants, we did not pursue this line of inquiry at this point.

6. It would be useful to provide a list of all the Roadmap annotation used (or at least the cell types), so that readers know which annotations were not prioritized. Perhaps a full list of Figure 5A could be included in the supplement.

We added tables containing the effect size estimates from various model fits. We added a table that maps expecto annotations to the roadmap celltypes.

7. Figure 1B seems more complicated than necessary. Maybe a grid would be a better tool for representation.

We thank the reviewer for this much simpler solution. We changed the figure according to the suggestion.

8. It is difficult to follow the trend lines and see the different layers in Figures 2A and 4B, especially in Figure 4B.

Figure 4B was changed to only plot the top quartile. This allowed us to plot results for many model fits together.

9. Including the tissue sample size or number of eQTLs per tissue might add more meaning to Figure 5A.

The fitted eQTL experiments are all relatively close in sample size ranging from 300 to 491 (see GTEx project page). We suspect that the different qualities of the various fits are driven by the adequacy of the annotations and the complexity of the tissue rather than difference in sample size. We therefore fear that it could be potentially confusing to add the sample size to the figure.

10. Since it looks like there are a couple outliers, it might be more informative to sort the x-axis of Figure 5B by median instead of by maximum. And it would be interesting to know which tissues those outliers came from.

We would like to argue that outliers are informative in this case, as they imply that a particular variable was selected. Meanwhile, we think the ranks in medians are not meaningful if they are too close to zero. When looking at the outlier for *DNase.fdr0.01.peaks*, we found Lung tissue, which incidentally, had the lowest value among *DNase.all.peaks*. (The *DNase.all.peaks* outlier was observed in transformed fibroblasts, in agreement with the low relative MSE_{dir} achieved in that experiment. We added a short mention of this to the figure text.

11. It appears the GTEx data were analyzed using a EUR-only LD matrix, when the sample is ~85% EUR.

We made a mention in the outlook that handling LD matrices from other cohorts could be a future improvement to BAGEA (See Discussion paragraph starting *An avenue also not explored here*)

12. The method does not use INDEL information (I believe).

We made a mention in the outlook that handling INDELS could be a future improvement to BAGEA (See Discussion paragraph starting *An avenue also not explored here*)

13. I tried to download and test the software myself, but the install script would not work; it failed on "devtools::use_rcpp()" with the message "Error: 'use_rcpp' is not an exported object from 'namespace:devtools'". I did not investigate further.

This seems to be due to a backward compatibility issue of devtools, and should be fixed now.

Reviewer #2

The underlying model is a "soft sparse" Bayesian regression with ARD prior on coefficients (this gives a student-t distribution on each coefficient, the authors should check that and mention it i so).

This is indeed the case and this fact is now mentioned.

I think it might be helpful to give this explanation in the text since the matrix version is a bit more obtuse.

We adjusted the text accordingly.

There are small number of typos I've annotated on the attached manuscript

We thank the reviewer warmly for this helpful addition. We corrected the typos (one exception was the concern about the overloaded notation of lambda. We apologize that we left this as is as this change would also affect the code base).

The model itself is very reasonable. The main aspect one could argue about is whether this approach of "soft sparsity" is competitive with spike-and-slab priors as used in Bayesian fine-mapping approaches such as enloc or Matt Stephen's recent SuSiE method.

Indeed, the model could be used for fine mapping and it would be interesting to compare relative performance to other fine mapping tools. As the framework is relatively flexible, one could assume that performance also varies with different hyperparameter settings. However, we decided to focus in this manuscript rather on directed annotation selection and gene expression prediction as this is where the main novelty lie in our estimation.

I would be curious to know what are the reason(s) driving this: variant effector predictors aren't good enough? insufficient sample size for training BAGEA (seems unlikely)? Chromatin state is in only proxy cell types?

This is indeed a very important question. One could argue that the newly added simulation study might suggest that insufficient sample size is not the problem as recovery of the true underlying signal was quite feasible when the true underlying model explained about as much variance as was seen empirically (See Results Subsection *Exploring BAGEA's ability to identify causal marks through simulation*). If there were an underlying model that would explain substantially more variation, it stands to reason that it would be even easier to fit. The fact that chromatin state is only measured in proxy cell types, might explain some of the poor fits, as the highest effect sizes are reached in well covered cell types (i.e. Fibroblast, Whole Blood). Finally, variant effector predictors are relatively novel, so there might still be large improvements possible.

While the small pve is what it is, I would appreciate some attempt to benchmark vs existing eQTL/annotation approaches like eQTNMiner, enloc or stratified LD score regression. I realize this is non-trivial since existing approaches can't predict gene expression, and there is no ground truth for what annotations should be important for eQTLs in a given cell type. That leaves simulations, which are likely to bias towards which model is most similar to the simulation, and fine mapping. Fine mapping might be a reasonable way to compare (although I realize the method is not specifically tailored to this). You could think of downsampling individuals and see how well you recover the credible set of variants from the full data.

We opted for two comparisons, one against Torus (part of the enloc pipeline) and ExPecto. For ExPecto, we used model fits for the GTEx data sets that were released as part of the original ExPecto publication. (See Figure S7). This showed that BAGEA had favourable performance for genes with large directed predictors. We also fleshed out some reasoning in the Discussion (see paragraph starting *Using directed annotations to ..*), outlining why we think that the general modeling approach pursued in the ExPecto publication might not yield optimal results in the long run.
Further, we added a comparison of BAGEA with Torus with l_2 penalty (We ran this comparison on an additional analysis where the encode-TF annotations were used). While BAGEA did show improvement over Torus w.r.t. Predicting causal SNPs, the improvement was marginal (See Figure S8 as well as Supplementary Methods). One plausible

explanation for this is that distance to TSS is already a very strong predictor of causal SNP location.

One thing I would emphasize is that this model could be applied to very rare, even de novo, variants. This distinguishes the approach from both existing eQTL and eQTL+annotation methods.

We added an outlook section to the Discussion mentioning this(See paragraph starting '*An avenue also not explored here'*).

Maybe I missed it but do you have a run time analysis? This is relevant for practitioners.

We added a run-time analysis (See Figure S15) for the main analyses included in the paper, in terms of algorithm update cycles. This shows that on a sixteen core machine, one fit (with 300 update cycles) took between 50 minutes to 6 hours depending on the annotation set used.

Is there a good reason the undirected annotations are binary? The math looks like it would all go through just fine with non-negative annotations.

BAGEA currently only implements binary undirected annotations, even though mathematically, this would not be required. It was done with performance in mind, as it allows to work with sparse boolean matrices. We mention this now explicitly when 0-1 coding is mentioned.

Reviewer #3

Major issues

*1. Model assumptions. There is generally a lack of discussions on the intuition and motivation of modeling decisions. First, a unique perspective of the proposed method is its ability to model *directed genomic annotations*. However, it is unclear how important are the *directions* of the annotations enforced in the model. In particular, are the omega parameters constrained (e.g., in the motivating example of binding affinity?). If yes, how? If not, I fail to understand how the concept of directed genomic annotations differs from a quantitative annotation.*

The 'directionality' implies that the annotation (once it is fitted) does not only allow to predict the presence or absence of an eQTL, but also in which direction a particular allele at that variant will shift expression. The directionality is therefore allele specific. In the motivating example of binding affinities this can potentially allow to deduce whether a TF is an activator or repressor. In fact, we added an additional analysis to attempt exactly that, allowing us to

define activators and repressors *de novo (*See Figure S13*).* Additionally, we added text to the example preparing the reader for this analysis. We hope that by connecting the motivational example with our actual analysis result, the reader will more easily see the difference to other approaches that use undirected annotations.

1. (cont) Relating to the same point, should the variance term alpha in Eqn (2) depend on the mean term too? Because a potentially large independent variance term can make the directional effect meaningless.

The BAGEA tries to model the observed eQTL effects with a mean shift component that is derived from directed annotations and residual effect unexplained by the directed annotations. Separating the two clearly, allows us to derive clear metrics of how much of the expression variance is explained by the directed components.

*1. (cont) Second, the joint modeling of directed and undirected annotations in Eqn (2) is not intuitive. It is unclear to me why it is a reasonable strategy that the model *only* consider the interactions between the two distinct categories. The particular model formulation also causes confusions: consider a single directed annotation and a single undirected annotation, would the absence of the undirected annotation (i.e., 0) completely remove the effect of the quantitative information from the directed annotation?*

In practice, BAGEA automatically adds an intercept term by default, i.e. the first column of F^j is always a vector of ones and the priors are adjusted to constrain the estimated effect size for this intercept terms (i.e. nu_1) very close to 1 too. While this is implicitly visible in Figure 3D (Fnu is fixed to 1 outside of a window of 50KB) and the intercept is sometimes mentioned in figure captions (and the BAGEA help functions), we have not explicitly mentioned this in the model setup. We have remedied that now (See Results section: Model Overview, bullets describing F^j and nu). We thank the reviewer to make us aware of this oversight.

2. Evaluation of the model. The authors chose to evaluate the proposed model based on its predictive performance. However, their results show extremely inaccurate predictions at the absolute scales (1.5% variance explained). It is well known that gene expressions are generally difficult to predict, but other available genotype predictive approaches, e.g., the simple elastic net algorithm implemented in PrediXcan method, show much higher heritability based on similar datasets (See https://www.ncbi.nlm.nih.gov/pubmed/27835642). Can the authors explain the discrepancy? On the surface, this seems to undermine the main point of the paper: by introducing an annotation-informed prior, I'd expect at least similar, if not much better, predictive performance (an annotation-uninformed model should be included as a special case of the proposed model by setting appropriate nu, omega and alpha parameters).

The way that the model was evaluated, was in terms of predicting gene expression values for genes on which the model wasn't trained on, simply using the undirected annotations available (i.e. the gene to predict is not in the training set; only global variables are used to predict). This is a more challenging problem than predicting in a new sample the expression of a gene that the model was trained on (i.e. the gene to predict is in the training set; local variables are used to predict). To differentiate the two problems more clearly, we added an analysis, where we split the monocyte expression data into the two underlying datasets, fit BAGEA on one dataset to derive effect size estimates (i.e. b_j vectors in BAGEA's notation) for each gene with a substantial eQTL on chromosome 1 to 15 and then used those estimates to predict gene expression in the other dataset See Figure S2 and Results paragraph starting with *These results show..*). When comparing the average variance explained to the average total genetic variance in *cis* (as estimated by Haseman-Elston regression), we saw that about 2 thirds of it was explained. When restricting BAGEA priors to an annotation uninformed model, we saw the variance explained drop significantly. We regard this analysis to serve mainly illustrative purposes though, as the main novelty lies within the use of the directed annotations allowing us to predict gene expression from global parameters (i.e. omega and nu) only.

3.The relevance of genomic annotations. More details should be provided on this topic. Although some computational difficulty is acknowledged in the discussion, the adopted training and testing protocol should be evaluated in a simulation setting.
We added a simulation study to investigate how well BAGEA was able to select causal genomic annotations and compared the derived predictive models with the optimal models (i.e. if the truth were known). In general, BAGEA correctly isolated causal genomic annotations if the models were sufficiently sparse, and predictive performance was good (See Results subsection *Exploring BAGEA's Ability to Identify Causal Marks through Simulation and accompanying supplementary figures).*

3 (cont.) It is also vague from the paper if an individual annotation is assessed marginally or under the control of other competing annotations.

All fits were performed in a multivariate way i.e. non-marginally. This is now prominently mentioned (See the beginning of the Discussion and first mention of BAGEA in the introduction).

Minor comments:

1. undirected annotations are not formally defined as directed annotations.

We added a short definition in the introduction (see Introduction paragraph: *One strategy to build sequence-based models..*)

2. The GitHub page for the software needs to be improved. It does not have proper documentation or example datasets. I strongly encourage the authors to provide the datasets (i.e., the summary statistics from eQTL data and genomic annotations) analyzed in this paper online for the reproducibility purpose.

We added more details to the GitHub page. There should be enough guidance to: 1) install the software 2) run an example. The example uses all of the main functionality of BAGEA. Further details are available via the R help function. We prepared an S3 bucket with auxiliary data as well as the directed annotation (See Links in manuscripts), BAGEA offers automatic installation from this bucket. Further, BAGEA has helper function for downloading and preprocessing GTEx summary statistics data in accordance with the manuscript.
