# Supplementary material for: A framework for integrating directed and undirected annotations to build explanatory models of cis-eQTL data
Source: PLoS Comput Biol. 2020 Jun 9;16(6):e1007770. doi: 10.1371/journal.pcbi.1007770 (PMC7332077; doi:10.1371/journal.pcbi.1007770)
Supplement: Attachment — Submitted filename: cover letter _rebuttal_second_round_pretty.pdf [file pcbi.1007770.s024.pdf]

February 19, 2019

Roger Pique-Regi, Ph.D.
Guest Editor
PLOS Computational Biology

Weixiong Zhang, Ph.D.
Deputy Editor
PLOS Computational Biology

Dear Dr. Pique-Regi and Dr. Zhang,

Thank you for examining our revised manuscript and the favourable reviews.
In our view, the reviewers' suggestions have helped to improve the manuscript. With the help of the Reviewer #1's comments, we made edits to the manuscript to add clarifications and further discussions of our findings. **Our specific changes are described below (attached to this letter).** We have uploaded the revised manuscript using the PLoS Editorial Manager web portal.

Best Regards,
Victor Hanson-Smith, PhD
Head of Computational Biology
Verge Genomics

Verge Genomics · 2 Tower Place, Suite 950 · South San Francisco, CA, 94080 · vergegenomics.com

Reviewer #1:

Minor issues

1. In "Joint modeling of cis-eQTLs and Directed Annotations..." you mentioned a cell line rationale for the prioritized TF annotations. Were the expected TFs also prioritized, or did the effects appear to be mostly driven by cell lines?

We added a detailed discussion of the prioritized TFs and their respective direction in light of available literature (See end of the section in question, last two paragraphs). An effort was made to not overstate our findings by highlighting potential biases in the dataset, that could complicate interpretation. We found that the selected TFs seemed overall reasonable candidates based on the available literature. This is of course a subjective statement and we tried to allow the reader to draw their own conclusions.

2. & 5. Line 352, "annotation" sp.
Fig 4. caption partially sp.

We thank the reviewer warmly for the attention to detail on behalf of our manuscript!

3. Was torus run using all eQTLs (all p-values)? Or were the same variants used for both methods (p<10e-7)?

Torus was run on the same SNP-gene pairs as BAGEA i.e. with the same filters applied. This was somewhat buried in the supplemental methods. We mention this now in the main text and specifically reference the supplement (see section '*Building Predictive Expression Models..*' paragraph starting '*We further compared BAGEA to Torus ..*').

4. Could comment #3 possibly explain why BAGEA showed no effect for many non-zero torus effects (Fig S9)? It's also possible that I'm misunderstanding the text -- I'm not entirely sure that I understand the last sentence of the paragraph (lines 360/361).

We tried to clarify our effect size comparison approach (See end of last paragraph in Section '*Building Predictive Expression Models ..*'). We believe that the many non-zero effects were due to the regularization chosen (l_2, i.e. *ridge*). We chose this regularization for practical reasons as there were fewer convergence issues (potentially due to the high number of annotations evaluated together, but we do not explicitly mention this because we have not evaluated that question systematically, as we felt this to be out of scope for the manuscript).

6. Fig 4A: What's going on with Adipose Omentum -- why is the RMSE at 1? Apologies if I missed this discussion in the text.

BAGEA does not necessarily select any variables if none have a sufficiently good fit. We added a short discussion of this fact.
(See section ‘*Building Predictive Expression Models from GTEx Summary Statistics’.* Sentence starts with: *‘We saw that the ENCODE TF annotation set tended to outperform..’*

7. When discussing DNase1 data processing, it might be helpful to mention that DNase1 hotspots are usually wider than DNase1 peaks calls (I believe).

This is indeed the case and we mention this fact now. (See section ‘GTEx Expression Models Make Use of Biologically Relevant Annotations’ , End of second paragraph)

Again, we would like to thank all reviewers for the constructive comments which, we believe, helped us to improve the manuscript substantially.
